# Evolutionary History and Phylodynamics of Influenza A and B Neuraminidase (NA) Genes Inferred from Large-Scale Sequence Analyses

**DOI:** 10.1371/journal.pone.0038665

**Published:** 2012-07-11

**Authors:** Jianpeng Xu, C. Todd Davis, Mary C. Christman, Pierre Rivailler, Haizhen Zhong, Ruben O. Donis, Guoqing Lu

**Affiliations:** 1 Department of Biology, University of Nebraska at Omaha, Omaha, Nebraska, United States of America; 2 Influenza Division, Molecular Virology and Vaccines Branch, Centers for Disease Control and Prevention, Atlanta, Georgia, United States of America; 3 Department of Chemistry, University of Nebraska at Omaha, Omaha, Nebraska, United States of America; University of Hong Kong, Hong Kong

## Abstract

**Background:**

Influenza neuraminidase (NA) is an important surface glycoprotein and plays a vital role in viral replication and drug development. The NA is found in influenza A and B viruses, with nine subtypes classified in influenza A. The complete knowledge of influenza NA evolutionary history and phylodynamics, although critical for the prevention and control of influenza epidemics and pandemics, remains lacking.

**Methodology/Principal findings:**

Evolutionary and phylogenetic analyses of influenza NA sequences using Maximum Likelihood and Bayesian MCMC methods demonstrated that the divergence of influenza viruses into types A and B occurred earlier than the divergence of influenza A NA subtypes. Twenty-three lineages were identified within influenza A, two lineages were classified within influenza B, and most lineages were specific to host, subtype or geographical location. Interestingly, evolutionary rates vary not only among lineages but also among branches within lineages. The estimated tMRCAs of influenza lineages suggest that the viruses of different lineages emerge several months or even years before their initial detection. The *d*_N_ */d*_S_ ratios ranged from 0.062 to 0.313 for influenza A lineages, and 0.257 to 0.259 for influenza B lineages. Structural analyses revealed that all positively selected sites are at the surface of the NA protein, with a number of sites found to be important for host antibody and drug binding.

**Conclusions/Significance:**

The divergence into influenza type A and B from a putative ancestral NA was followed by the divergence of type A into nine NA subtypes, of which 23 lineages subsequently diverged. This study provides a better understanding of influenza NA lineages and their evolutionary dynamics, which may facilitate early detection of newly emerging influenza viruses and thus improve influenza surveillance.

## Introduction

Influenza virus belongs to the viral family Orthomyxoviridae and has a segmented negative-sense RNA genome in an enveloped virion [1]. According to the antigenic properties of nucleoproteins (NP) and matrix proteins (MP), influenza viruses are classified into three types - A, B and C. The microscopic structural features and genome organization of influenza A, B and C viruses suggest that they descended from a common ancestor [2]. The influenza A virus infects a wide variety of bird and mammalian species and can cause moderate to severe epidemics annually and catastrophic pandemics sporadically [2], [3]. The influenza B and C viruses are considered less pathogenic compared with influenza A and are found mainly in humans, although there is increasing evidence that B and C viruses can also infect other species [4].

Genetic mutation is considered one of the most important molecular mechanisms in the evolution of influenza virus [5]. Like most RNA viruses, the influenza virus has low fidelity RNA synthesis, which results in a high mutation rate - around one mutation per genome per replication [6], several orders of magnitude higher than those in most DNA-based organisms [7]. Evolutionary forces such as natural selection acting upon rapidly mutating viral populations could shape the genetic structure of influenza viruses in different hosts, geographic regions and periods of time [8], [9]. Importantly, rapid evolution could partially facilitate the ability of influenza viruses to cross host species barriers and successfully emerge in new hosts with often important public health and/or veterinary health implications. One such example is the Eurasian avian-like H1N1 swine virus, which was first detected in pigs in Belgium in 1979, with all of the eight segments found to be derived from a Eurasian avian H1N1 virus, presumably following adaptive mutation [10].

Influenza virus has also shown the propensity to escape immunity because of continuous antigenic drift, i.e., mutation at the epitope positions of HA and NA segments [11], [12]. Antigenic drift may often result in structural changes in antigenic sites, which must be recognized by the host immune system in order to suppress viral infection [13]. This antigenic drift often requires the update of annual influenza vaccines to assure a match between the vaccine and currently circulating viral strains [14]. Additionally, influenza viruses undergo more dramatic antigenic changes, known as antigenic shifts, which occur following reassortment between different subtypes of influenza viruses within a single host [2].

Each of the influenza viral genes is thought to be important in viral replication and interaction with host cells; therefore, understanding the evolutionary tempo and mode of each viral gene can provide new insight into the epidemiology of influenza viruses [15], [16]. Among the eight segments, neuraminidase (NA) is of particular significance. NA is a major surface glycoprotein of influenza A and B, but does not occur in influenza C [17]. It plays a key role in virus replication by removing sialic acids from the host cell surface and thus releasing newly formed virions [18]. Drugs that inhibit this NA activity, known as neuraminidase inhibitors, are often used for the treatment of influenza [19]. However, drug resistance mutations (e.g., H275Y) have been broadly observed in epidemic viruses [20].

Influenza A viral neuraminidases are classified into nine subtypes (N1–N9) according to their antigenic properties, whereas influenza B neuraminidases are classified into two lineages [21]. Previous phylogenetic analyses of influenza viral NA sequences have provided important insight into understanding the evolution of influenza viruses; however, these studies mainly focused on either specific types or subtypes [15], [22], [23], [24]. A global perspective of the evolutionary history of influenza NA genes and their spatial, temporal, and host associations remain lacking. In addition, evolutionary rates of influenza viral genes were estimated (∼10^−3^ substitution/site/year) in previous studies [23], [25], [26]; however, only the average values across all branches were presented. It is unlikely the evolutionary rates are the same in all branches within a phylogenetic tree. The investigation of rate variations among different branches is thus of significant importance in understanding the interior evolutionary behavior of the influenza virus. Moreover, selection pressure and positive/negative selection sites were described in previous studies [23], [26], but only a small number of representative sequences were selected for the estimations. Finally, the structural analysis of positively selected amino acid sites, although essential for the development of antiviral drugs and vaccines, has largely been neglected in previous studies. In this study, we employed all influenza NA sequences available in public repositories and conducted large-scale evolutionary, phylodynamic and structural analyses to address the above issues.

## Results

### Global Picture of Evolutionary Relationships of Influenza A and B Neuraminidase (NA) Genes

**Figure 1:**
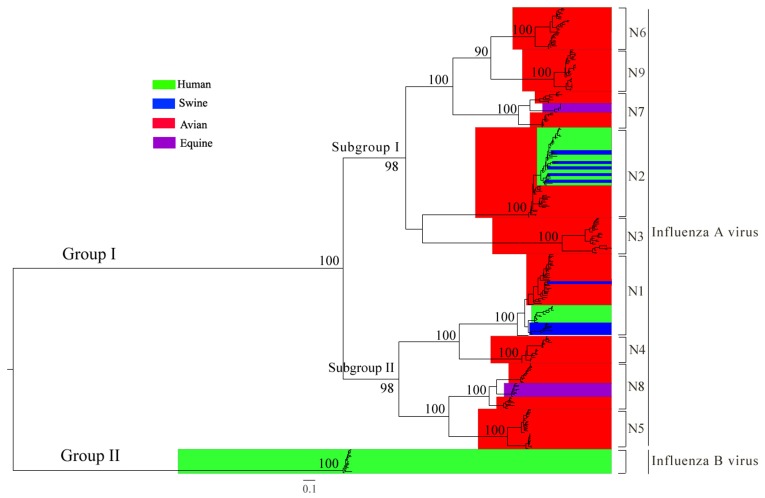
Phylogeny of influenza A and B neuraminidase (NA) genes. Influenza NA genes form two groups (Group I and Group II), which correspond to influenza A and B, respectively. Influenza A NA is further classified into two subgroups (Subgroup I and Subgroup II). The viral strains are colored for different hosts: human in green, swine in blue, avian in red and equine in purple. The bootstrap support values are indicated at major nodes. The scale bar at the bottom indicates the numbers of nucleotide substitutions per site.

The Maximum Likelihood (ML) and MCMC Bayesian analyses demonstrate that the influenza NA gene diverged first into A and B (Group I and Group II), followed by the division of influenza A subtypes (Figure 1, File S1). The monophylic origin of influenza A and influenza B was strongly supported by the bootstrap values (100%). Within influenza A, two subgroups were found, one consisting of subtype N2, N3, N6, N7 and N9 (Subgroup I) and the other consisting of the remaining four subtypes, N1, N4, N5 and N8 (Subgroup II) (Figure 1). Each subgroup consists of viruses independently adapted to the avian, human, equine and swine hosts, indicating that parallel evolution occurred in these two subgroups (Figure 1). In addition, each of the nine influenza A NA subtypes was found to form a distinct cluster with a high bootstrap support value (>90%), indicating a monophyletic origin for each subtype.

### Phylogeny of Neuraminidase (NA) Genes within Influenza A and B Viruses

A total of 23 lineages, two to three lineages for each subtype, were identified within influenza A viruses, while two lineages were classified within influenza B (Table 1). Lineages 1A and 2A were further divided into five and three sublineages, respectively. Human lineages were found in influenza A N1 and N2 subtypes and influenza B, swine lineages in N1 and N2, equine lineages in N7 and N8, and avian lineages in all influenza A subtypes. In addition, avian lineages were found to have more combinations of HA and NA compared with mammalian lineages.

**Table 1:** The annotations, isolation periods, representative sequences and subtypes for the NA lineages.

Influenza	Subtype	Lineage/Sublineage	Annotation	Isolationperiod	Representative sequence	Main virus subtypes
**A**	N1	1A.1	H5N1	1996–2010	A/Goose/Guangdong/1/96(H5N1)	H5N1
		1A.2	Eurasian avian	1934–2009	A/fowl/Rostock/45/1934(H7N1)	H1N1, H3N1, H5N1, H6N1, H7N1,H9N1, H11N1
		1A.3	Pandemic H1N1 2009	2009–2010	A/Texas/05/2009(H1N1)	H1N1
		1A.4	Eurasian (avian-like) swine	1979–2010	A/swine/Belgium/WVL1/1979	H1N1
		1A.5	North American avian	1969–2008	A/duck/PA/486/1969(H6N1)	H1N1, H3N1,H4N1, H5N1, H6N1,H10N1, H12N1
		1B	North American swine	1930–2009	A/swine/Iowa/15/1930(H1N1)	H1N1, H3N1
		1C	Major human	1918–2009	A/Brevig_Mission/1/18(H1N1)	H1N1
	N2	2A.1	H9N2	1994–2009	A/chicken/Guangdong/SS/94	H9N2
		2A.2	Eurasian avian	1977–2008	A/duck/Hokkaido/5/1977	H3N2, H5N2, H6N2, H7N2, H9N2, H11N2
		2A.3	North American avian	1966–2008	A/turkey/Wisconsin/1/1966(H9N2)	H3N2, H4N2,H5N2, H6N2, H7N2, H9N2, H11N2, H10N2, H13N2
		2B	Major human and swine	1957–2009	A/Japan/305/1957	H3N2, H2N2, H1N2
	N3	3A	North American avian	1971–2010	A/turkey/Oregon/1971(H7N3)	H7N3, H4N3, H1N3, H10N3, H11N3,H6N3, H5N3, H3N3, H2N3
		3B	Eurasian/Oceanian avian	1959–2009	A/shearwater/Australia/751/1975(H5N3)	H1N3, H5N3, H4N3, H3N3, H8N3, H12N3, H7N3, H2N3, H10N3, H11N3, H9N3
		3C	Other avian	1975–2009	A/sabines gull/Alaska/296/1975(H5N3)	H7N3, H16N3, H3N3, H13N3, H5N3
	N4	4A	North American avian	1967–2010	A/turkey/Ontario/6118/1967(H8N4)	H3N4, H8N4, H12N4, H4N4, H2N4
		4B	Eurasian/Oceanian avian	1979–2008	A/gray teal/Australia/2/1979(H4N4)	H4N4, H8N4, H9N4,H10N4
	N5	5A	North American avian	1976–2009	A/mallard duck/ALB/60/1976(H12N5)	H12N5, H1N5, H11N5, H3N5, H6N5,H4N5, H5N5, H2N5, H9N5, H10N5, H7N5
		5B	Eurasian/Oceanian avian	1972–2009	A/shearwater/Australia/1/1972(H6N5)	H6N5, H1N5, H3N5, H8N5, H10N5,H12N5, H4N5, H14N5
	N6	6A	North American avian	1976–2010	A/mallard duck/ALB/20/1976(H4N6)	H3N6, H4N6, H10N6, H6N6, H1N6
		6B	Eurasian/Oceanian avian	1956–2010	A/duck/Czech Republic/1/1956(H4N6)	H4N6, H3N6, H5N6, H9N6
	N7	7A	North American avian	1977–2010	A/mallard duck/ALB/302/1977 (H10N7)	H4N7, H10N7, H3N7, H2N7, H7N7,H5N7, H8N7, H13N7
		7B	Eurasian/Oceanian avian	1902–2008	A/chicken/Brescia/1902(H7N7)	H7N7, H10N7, H5N7, H11N7
		7C	Equine	1956–1977	A/equine/Prague/1/1956(H7N7)	H7N7
	N8	8A	North American avian	1963–2010	A/turkey/Canada/1963(H6N8)	H3N8, H4N8, H6N8, H7N8, H2N8, H10N8
		8B	Equine	1963–2010	A/equine/Miami/1/1963(H3N8)	H3N8
		8C	Eurasian/Oceanian avian	1963–2010	A/duck/Ukraine/1/1963(H3N8)	H3N8, H10N8, H11N8, H6N8, H7N8,H2N8,H4N8
	N9	9A	North American avian	1966–2008	A/turkey/Ontario/7732/1966 (H5N9)	H11N9, H13N9, H12N9, H5N9, H10N9,H3N9, H2N9, H1N9, H7N9, H4N9
		9B	Eurasian/Oceanian avian I	1996–2010	A/duck/Siberia/700/1996(H11N9)	H11N9, H5N9, H7N9, H6N9, H2N9, H1N9
		9C	Eurasian/Oceanian avian II	1978–2004	A/duck/Hong Kong/278/1978(H2N9)	H11N9, H5N9, H15N9, H10N9, H2N9
**B**		Yam88	B/Yamagata/16/88-like	1988–2009	B/Yamagata/16/1988	
		Vic77	B/Victoria/2/87-like	1987–2002	B/Victoria/2/1987	

#### Lineage analyses of influenza A N1 genes

**Figure 2:**
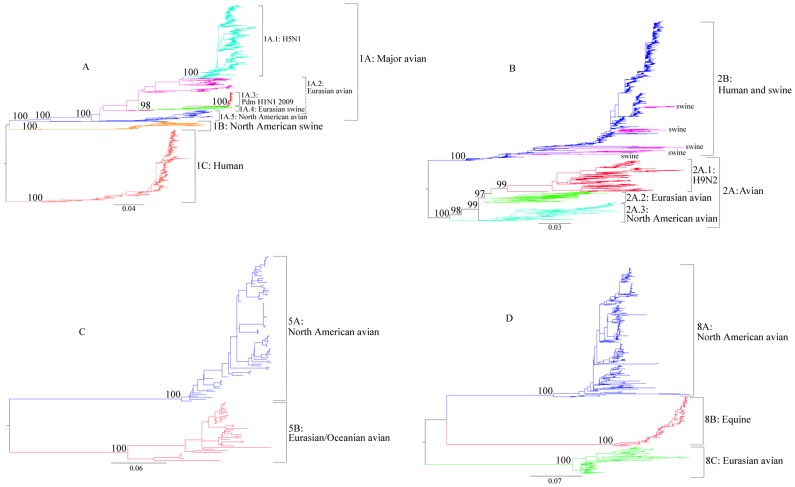
Maximum-likelihood (ML) tree of influenza A NA subtypes. A: N1; B: N2; C: N5; D: N8. The annotation for each lineage was labeled on the trees. Three lineages in N1 (1A, 1B and 1C), two lineages in N2 (2A and 2B), two lineages in N5 (5A and 5B), and two lineages in N8 (8A and 8B) were classified. The bootstrap values supporting the corresponding lineages are shown to the left of the major nodes. Scale bars indicate the numbers of nucleotide substitutions per site.

Three lineages, 1A, 1B and 1C, were identified based upon strong bootstrap support values (100%) of the phylogenetic tree, which was generated from 4,146 sequences (Figure 2-A, Table 1). The genetic distances between lineages ranged from 0.191 to 0.238. Lineage 1A is a major avian lineage, which is further divided into five sublineages: 1A.1 (H5N1), 1A.2 (Eurasian avian), 1A.3 (Pandemic H1N1 2009), 1A.4 (Eurasian avian-like swine) and 1A.5 (North American avian).

Sublineage 1A.1 originated from the recent highly pathogenic H5N1 avian influenza epizootic that started in Asia around 1996 and has spread throughout the Eastern Hemisphere. The viruses in 1A.1 are mostly from birds (n = 1,031), but some are from humans (n = 164), swine (n = 8), tigers (n = 2) and mink (n = 1). Sublineage 1A.2 is composed of mostly Eurasian avian influenza viruses (n = 230), whereas some human highly pathogenic H5N1 influenza viruses (n = 24) sampled in 1997 in Hong Kong were also found in 1A.2. Sublineage 1A.4 consists of Eurasian swine influenza viruses which were originally derived from Eurasian avian viruses and first detected in Belgium in 1979. Not surprisingly, 1A.3 (Pandemic H1N1 2009) is grouped together with Eurasian swine, which confirms previous findings that the NA segment of pandemic H1N1 2009 viruses originated from the Eurasian swine influenza viruses. Sublineage 1A.5 is composed of viruses mainly from North American avian species (n = 162), with a few exceptions: 1 viral sequence from human and 3 from environmental samples.

Lineage 1B consists of mainly North American swine influenza viruses, while 1C is a human lineage, consisting mainly of H1N1 human influenza viruses. The viruses in 1B correspond mostly to the classical H1N1 isolates from swine (n = 126), but include 9 isolates from humans and 9 from birds, indicating sporadic interspecies transmissions of influenza viruses from swine to humans or birds. Lineage 1C consists predominantly of human viruses (n = 1204), with a few exceptions, namely, swine (4 isolates) and birds (2 isolates). Within the influenza A N1 subtype, avian influenza viruses include sequences from multiple HA subtypes (e.g., H1N1, H3N1, H5N1, H6N1, H7N1, H9N1, and H11N1), whereas human and swine viruses have limited HA subtypes (human: H1N1; swine: H1N1, H3N1).

#### Lineage analyses of influenza A N2 genes

The N2 sequences (3,754 in total) were classified into two major lineages, 2A and 2B (Figure 2-B, Table 1). The genetic distance between lineages 2A and 2B was estimated to be 0.204. Lineage 2A is a major avian lineage whereas 2B consists of mainly mammalian (i.e., human and swine) influenza viruses. Three sublineages were further classified in 2A, 2A.1 for H9N2, 2A.2 for Eurasian avian, and 2A.3 for North American avian.

The 2A.1 is a subtype-specific sublineage consisting of mainly H9N2 avian influenza viruses, with the majority from birds (n = 412), but with 24 sequences from swine and 4 from humans, which indicates the occurrence of interspecies transmissions. The 2A.2 and 2A.3 correspond to Eurasian and North American avian viruses, respectively. The viruses of 2A.2 are mainly from birds (n = 342), but a few are from swine (n = 7) and humans (n = 2). A similar result was also found in 2A.3, which includes 291 avian viruses, 1 H7N2 human virus, and 29 viruses isolated from environmental samples.

Within 2B, most of the influenza viruses are from human H2N2 and H3N2 influenza viruses (n = 2,340) and swine H3N2 and H1N2 viruses (n = 214). However, avian influenza H3N2 viruses (n = 11) were also found in this lineage. Interestingly, there were five major clades of swine influenza viruses scattered within lineage 2B, suggesting these viruses originate from human viruses through either genome reassortment or direct transmission events. It is also noted that the branch lengths of the swine clusters are much longer as compared to those of the closely related human viruses, indicating extensive evolution of the N2 gene in swine viruses after transmission from humans to swine.

#### Lineage analyses of influenza A N3–N9 genes

Three lineages, 3A, 3B, and 3C, were found in N3, with genetic distances between lineages ranging from 0.173 to 0.349 (Table 1, Figure S1). Lineage 3A consists mainly of North American avian viruses (n = 173), but includes several avian strains from South America (n = 8). In addition, within lineage 3A, 166 sequences were isolated from avian, 4 from swine, 1 from human, and 9 from environmental samples. Lineage 3B is a Eurasian/Oceanian avian lineage, while 3C is also an avian lineage, but does not show any geographical pattern. Lineage 3B and 3C were all composed of avian influenza viruses.

The N4, N5 and N6 subtypes were each classified into two lineages, one corresponding to North American avian (4A, 5A and 6A) and the other Eurasian/Oceanian avian (4B, 5B and 6B) (Table 1, Figure S2, Figure 2-C, Figure S3). The genetic distance between lineages was estimated to be 0.198 for N4, 0.254 for N5, and 0.250 for N6 viruses, respectively. All N4 and N5 viruses are from avian species. Lineage 6A is composed mainly of North American avian viruses (n = 336), with a few exceptions (n = 2) from Asia avian viruses. Lineage 6B consists mainly of Eurasian/Oceanian avian viruses (n = 121), but contains 6 avian viruses from North America.

Three lineages were identified in N7 and N8, which correspond to North American avian (7A, 8A), equine (7C, 8B) and Eurasian/Oceanian avian (7B, 8C), respectively (Table 1, Figure S4 and Figure 2-D). For N9, 3 lineages were identified: 9A, 9B and 9C, which correspond to North American avian, Eurasian/Oceanian avian I and Eurasian/Oceanian avian II, respectively (Table 1, Figure S5). The genetic distances between lineages were found in the range from 0.297 to 0.320 for N7, from 0.269 to 0.298 for N8, and from 0.117 to 0.224 for N9, respectively.

#### Lineage analyses of influenza B neuraminidase (NA) genes

**Figure 3:**
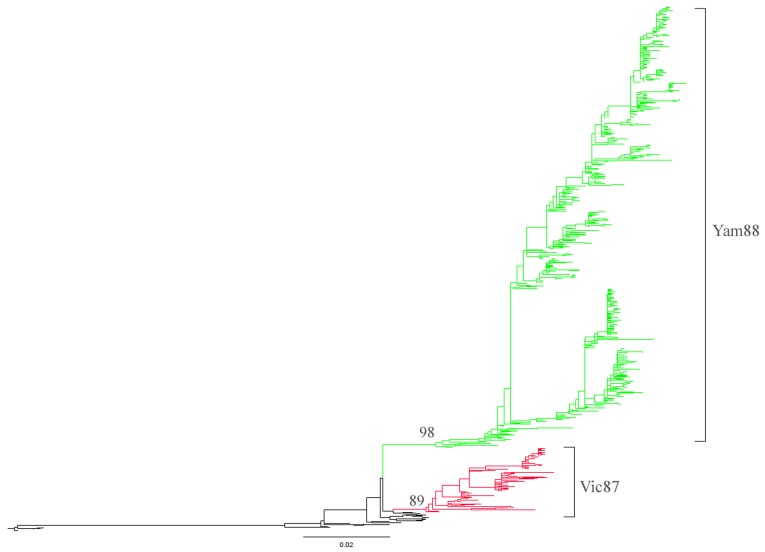
Maximum-likelihood (ML) tree of influenza B NA genes. Two lineages, Yam88 and Vic87, were classified. The bootstrap values supporting the corresponding lineages are shown on the major nodes. The scale bars indicate the numbers of nucleotide substitutions per site.

The NA genes of influenza B viruses were divided into two distinct lineages, B/Victoria/2/87-like (Vic87) and B/Yamagata/16/88-like (Yam88) (Figure 3). All influenza B viruses were found from humans, with no obvious geographical separation in either lineage. The genetic distance between Vic87 and Yam88 lineages was estimated to be 0.06.

### Substitution Rates and Times of Most Recent Common Ancestor (tMRCAs) of Influenza A and B NA Lineages

Outliers were identified and removed before the estimation of substitution rate and tMRCA for each lineage (Table S1). The mean substitution rate and 95% HPD range for each lineage are summarized in Table 2. Our results demonstrated that the mean substitution rates estimated under random local clock (RLC) model were generally lower than the corresponding rates estimated under uncorrelated exponential relaxed clock (UCED) model (Table 2). In the following, we present the results based upon the RLC model, a new model that can reveal the rate heterogeneity among branches.

**Table 2:** Substitutions rates and tMRCAs of different lineages for influenza A and B NA genes*.

Influenza	Subtype	Lineage/Sublineage	Substitution rate (×10^−3^ subs/site/year)			tMRCA (calendar year)		
			Mean	95%HPD lower	95% HPD upper	Mean	95% HPD lower	95% HPD upper
**A**	N1	1A.1	3.06/3.73	2.63/3.16	3.48/4.32	1988/1992	1984/1987	1992/1996
		1A.2	3.42/4.07	3.03/3.43	3.79/4.74	1927/1931	1922/1923	1931/1934
		1A.3	2.83/3.58	1.63/2.52	3.96/4.67	19-Nov-08/	7-June-08/	16-Mar-09/
						7-Dec-08	12-Jun-08	30-Mar-09
		1A.4	3.62/3.96	3.23/3.40	3.99/4.58	1978/1977	1977/1974	1979/1979
		1A.5	3.00/4.05	2.69/3.04	3.36/4.99	1921/1950	1911/1920	1934/1967
		1B	2.55/2.97	2.25/2.58	2.83/3.37	1929/1927	1928/1923	1930/1930
		1C	1.79/2.44	1.42/2.02	2.14/2.89	1898/1910	1882/1896	1909/1918
	N2	2A.1	4.45/4.61	4.07/3.98	4.89/5.24	1990/1989	1989/1984	1991/1993
		2A.2	2.53/2.81	2.25/2.38	2.81/3.26	1974/1972	1971/1963	1976/1977
		2A.3	2.96/3.19	2.66/2.73	3.26/3.68	1951/1954	1945/1937	1957/1965
		2B	3.05/3.31	2.74/2.91	3.89/3.75	1956/1956	1955/1954	1957/1957
	N3	3A	2.92/3.23	2.6/2.61	3.27/3.83	1954/1959	1944/1941	1963/1971
		3B	2.67/2.96	2.39/2.43	3.04/3.47	1955/1950	1950/1933	1957/1959
		3C	3.22/3.91	2.63/1.78	3.85/5.96	1955/1956	1949/1926	1961/1975
	N4	4A	3.37/4.30	2.82/3.39	3.93/5.27	1964/1966	1962/1962	1967/1967
		4B	3.78/4.42	3.09/2.75	4.5/5.98	1970/1970	1966/1956	1973/1978
	N5	5A	2.88/3.63	2.47/2.92	3.27/4.32	1971/1972	1968/1965	1975/1976
		5B	2.68/3.61	2.07/2.21	3.34/4.81	1953/1964	1941/1945	1963/1972
	N6	6A	2.1/2.32	1.88/1.86	2.3/2.79	1960/1955	1956/1934	1963/1970
		6B	2.69/3.08	2.39/2.55	2.97/3.63	1943/1940	1940/1920	1946/1952
	N7	7A	3.8/4.87	3.33/4.00	4.33/5.73	1975/1975	1974/1972	1976/1977
		7B	2.99/3.97	2.52/2.94	3.46/4.91	1892/1899	1882/1892	1901/1901
		7C	2.65/3.13	1.08/1.90	3.88/4.43	1952/1955	1940/1952	1956/1956
	N8	8A	1.54/2.31	1.36/1.93	1.73/2.71	1930/1956	1915/1941	1940/1963
		8B	−/1.68	−/1.37	−/2.02	−/1954	−/1945	−/1961
		8C	1.1/2.13	0.86/1.52	1.35/2.71	1921/1946	1904/1923	1937/1961
	N9	9A	2.8/3.36	2.49/2.77	3.13/3.92	1960/1961	1957/1952	1962/1966
		9B	2.75/3.32	2.19/2.41	3.39/4.21	1994/1995	1992/1992	1996/1996
		9C	−/2.16	−/0.24	−/3.95	−/1948	−/1890	−/1977
**B**		Yam88	2.30/2.47	1.99/2.08	2.62/2.85	1986/1986	1985/1982	1987/1988
		Vic87	1.90/2.14	1.50/1.65	2.3/2.62	1985/1985	1983/1982	1987/1987

* Values calculated based upon the random local clock model/values calculated based upon the uncorrelated exponential relaxed clock model; Dash signs (-) indicate missing data.

**Figure 4:**
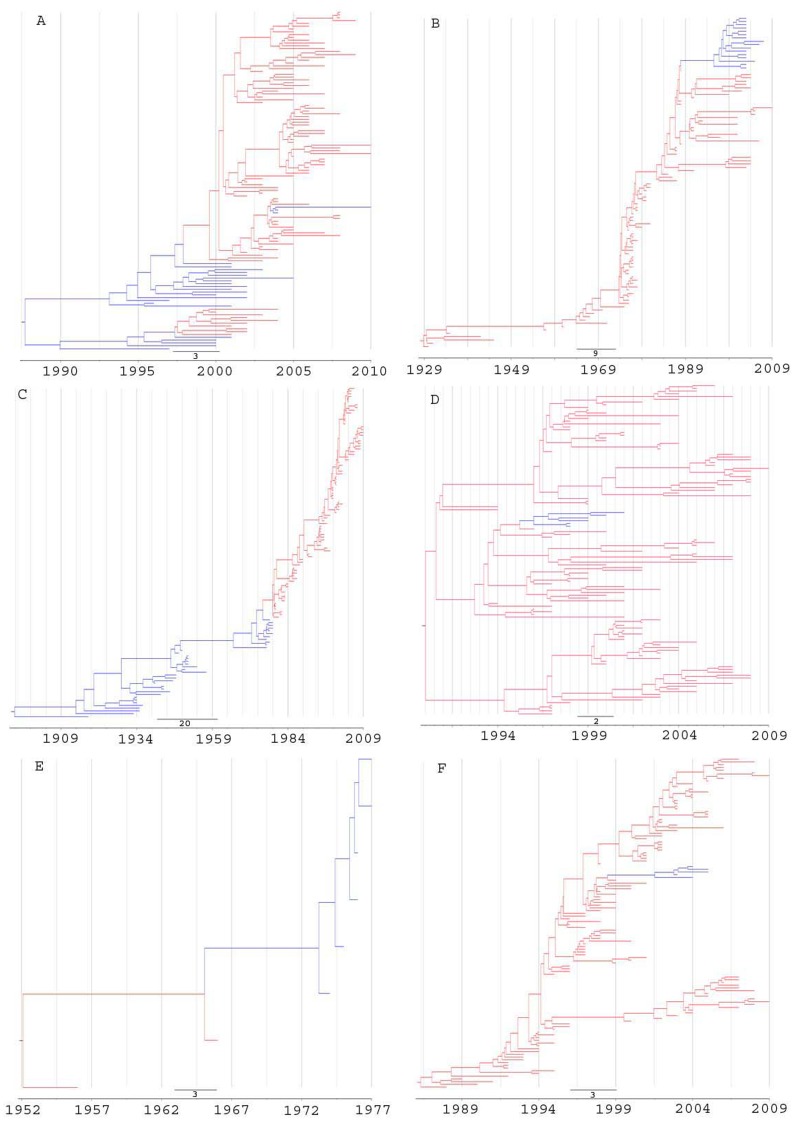
Bayesian inferences of random local clocks on influenza NA genes. A: H5N1 (1A.1), B: North American swine N1 (1B), C: Human H1N1 (1C), D: H9N2 (2A.1), E: Equine N7 (7C), F: Yama88 influenza B NA (Yama88). Branch coloring indicates inferred rates of nucleotide substitution from blue (slow) to red (fast). The scale bar indicates the number of years before the present.

The Bayesian consensus tree for each lineage, along with posterior mean branch lengths scaled in real time, is depicted in Figure 4. To reflect the rate variation, we colored branches by their posterior mean relative rate of nucleotide substitution. Blue branches reflect a slow substitution rate, whereas red branches indicate rapid change. For H5N1, the mean substitution rate was estimated to be 3.06×10^−3^ subs/site/year (Table 2), with a low rate (1.5×10^−3^) found in earlier branches (blue) and a high rate (4.20×10^−3^) in later branches (red) (Figure 4-A). In contrast, N1 genes of North American swine viruses have a mean rate of 2.55×10^−3^, with a decrease in rates during evolution: a high rate (3.2×10^−3^) in earlier branches (red) and a low rate (0.9×10^−3^) in later branches (blue) (Figure 4-B). It is noted that human H1N1 viruses were found to evolve at two different rates in two circulation periods, with a low rate (1.3×10^−3^) during 1918–1957 (blue) and a high rate (2.9×10^−3^) after 1977 (red) (Figure 4-C).

The H9N2 lineage was found to have a mean substitution rate of 4.45×10^−3^ (Table 2), with a constant rate of 4.9×10^−3^ in the majority of branches (red) and a low rate (2.6×10^−3^) in a small number of branches (blue) (Figure 4-D). The substitution rates with the equine N7 lineage decreased from earlier branches (red) (3.4×10^−3^) to late branches (blue) (1.6×10^−3^) and averaged at 2.65×10^−3^ (Figure 4-E). The influenza B Yama88 viruses has a mean substitution rate of 2.3×10^−3^ (Table 2), with a consistent rate of 2.4×10^−3^ in the majority of branches (red) and a rate of 1.5×10^−3^ in a small number of branches (blue) (Figure 4-F). Different rate heterogeneity patterns were also found in other lineages (Data available from authors on request).

The time of most recent common ancestor (tMRCA) varies from lineage to lineage (Table 2). The tMRCA for human H1N1 (1C), which includes viruses causing the 1918 Spanish Flu, was dated to 1898 and the 95% HPD interval was between 1882 and 1909. The tMRCA of H5N1viruses (1A.1) was estimated to be at 1988 (95% HPD: 1984–1992), eight years before the outbreak of H5N1 avian virus in 1996 in Asia. For 1A.2 (Eurasian avian in N1), the tMRCA was estimated to be at 1927 (95% HPD: 1922–1931), with the earliest sampling time being 1934. For the pandemic H1N1 2009 (1A.3), it can be dated back to Nov 19, 2008 (95% HPD: June 7, 2008– Mar 16, 2009). The most recent common ancestor of the Eurasian (avian-like) swine (1A.4) can be dated back to 1978 (95% HPD: 1977–1979), one year earlier than the first detection of this lineage in 1979. For lineage 2B, the tMRCA was dated to 1956 (95% HPD: 1955–1957), one year before the occurrence of human H2N2 in 1957. The tMRCAs for other lineages are shown in Table 2 and the MCC trees are available from the authors upon request. The above results suggest that pandemic or epidemic viruses emerged several months or several years before their initial detection, indicating the crucial role for enhanced surveillance of newly emerging viruses.

### Selection of Influenza A and B Neuraminidase Lineages

Different selection pressures were revealed in different lineages as indicated by the ratio of non-synonymous (*d*_N_) to synonymous (*d*_S_) substitutions per site (*d*_N_ */d*_S_) (Table 3). Within influenza A, the highest *d_N_/d_S_* ratio was observed in 2B - human N2 lineage (0.313), which was slightly higher than that of 8B - equine N8 lineage (0.281), 1C - human N1 lineage (0.261), 1A.1 - H5N1 (0.274) and 2A.1- H9N2 (0.252), most likely reflecting host immune selection pressure, as a result of continuous circulation within the respective hosts and/or vaccination. The lineages under the most purifying selection were lineage 9C (0.068), 4B (0.062) and 5B (0.078). In comparison, the *d_N_/d_S_* ratios for influenza B lineages were comparable: 0.259 for Yam88 and 0.257 for Vic87.

**Table 3:** Evidence of positive selection using the SLAC, FEL and IFEL methods with a significance level of 0.05.

Influenza	Subtype	Lineages/Sublineage	No. ofsequences	SLAC	FEL	IFEL	*d*_N_ */d*_S_(95% CI)
**A**	N1	1A.1	1241	16, 46, 83, 313, 340, 365	8, 339, 434	8, 16,46, 76, 339	0.274 (0.262–0.286)
		1A.2	263	460	20, 105, 460	20, 105, 454	0.202 (0.186–0.219)
		1A.3	794	None	53	53, 388, 452	0.227 (0.206–0.249)
		1A.4	80	None	None	210	0.180 (0.163–0.197)
		1A.5	228	None	449	95, 449	0.148 (0.135–0.162)
		1B	139	46	46, 53, 75, 81, 339	46, 53, 339, 453	0.174 (0.158–0.192)
		1C	1210	84, 222, 248	19, 84, 151, 222, 248, 319, 365	59, 222, 248, 344, 365	0.261 (0.249–0.274)
	N2	2A.1	586	9, 43, 50, 141, 199, 356	20, 43, 141, 199, 356	20, 43, 141, 199, 356	0.252 (0.240–0.264)
		2A.2	210	30	None	43	0.174 (0.162–0.186)
		2A.3	328	356, 416	113, 356, 414, 416	356, 414, 416	0.218 (0.204–0.233)
		2B	2169	5, 43, 56, 120, 126,148, 151, 370, 434	5, 43, 44, 56, 120, 126, 147,148, 151, 370, 434	43, 56, 127, 147, 267, 332,358, 370, 392, 455	0.313 (0.301–0.326)
	N3	3A	113	None	413, 432, 457	413	0.130 (0.115–0.146)
		3B	120	None	413	52, 413	0.161 (0.145–0.178)
		3C	9	None	None	None	0.092 (0.074–0.113)
	N4	4A	39	None	74	None	0.081 (0.065–0.100)
		4B	11	None	None	78	0.062 (0.047–0.080)
	N5	5A	68	None	30, 282	30, 282	0.140 (0.122–0.160)
		5B	17	None	None	30	0.078 (0.061–0.097)
	N6	6A	206	None	None	172	0.111 (0.100–0.123)
		6B	45	None	None	None	0.114 (0.100–0.129)
	N7	7A	90	None	None	None	0.153 (0.132–0.176)
		7B	42	None	42	None	0.092 (0.079–0.107)
		7C	10	None	None	None	0.135 (0.091–0.191)
	N8	8A	253	265	265	265, 376	0.128 (0.118–0.138)
		8B	95	None	None	None	0.281 (0.242–0.323)
		8C	61	None	35, 41	None	0.129 (0.114–0.145)
	N9	9A	76	None	None	None	0.095 (0.082–0.109)
		9B	25	None	None	None	0.106 (0.081–0.136)
		9C	9	None	None	None	0.068 (0.047–0.095)
**B**		Yam88	565	42, 65, 248, 373	65, 248, 345, 373, 395	42, 65, 248, 373, 389, 436	0.259 (0.238–0.281)
		Vic87	83	None	345	106, 345	0.257 (0.215–0.305)

Position relative to the start codon.

Human lineages were found to have the largest numbers of positively selected sites, with 16 sites for the human N2 lineage (2B), 9 sites for human H1N1 lineage (1C), and 8 sites for Yam88 lineage (Table 3). In addition, H5N1 (1A.1) and H9N2 (2A.1), have 10 and 7 positively selected sites, respectively. No positive selection sites were detected in lineages 3C, 6B, 7A, 7C, 8B, and 9A–9C. Other lineages were found to have one to six sites under positive selection.

**Figure 5:**
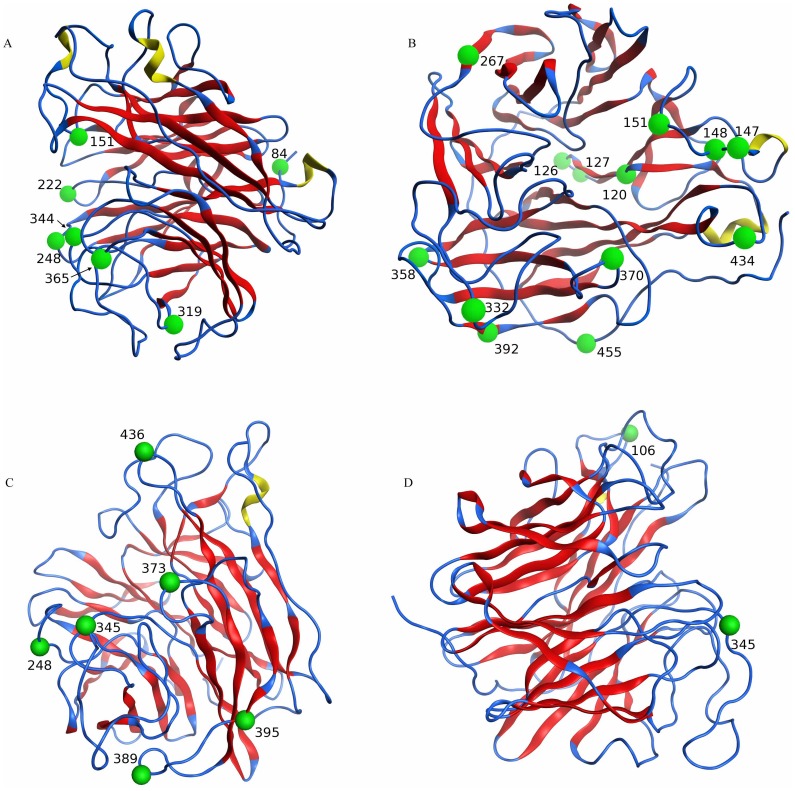
The structures and positive selection sites of human influenza neuraminidase. A: Influenza A human N1 neuraminidase (1C) (A/Brevig Mission/1/18 H1N1, 1918 “Spanish flu”, PDB ID: 3B7E); B: Influenza A human N2 neuraminidase (2B) (A/Tokyo/3/67 H2N2, 1967, PDB ID: 1IVG); C: Influenza B viral neuraminidase for Yam88 (B/Perth/211/2001, PDB ID: 3K36); D: Influenza B viral neuraminidase for Vic87 (B/Perth/211/2001, PDB ID: 3K36). The positive selection sites are denoted as green balls. Structural regions are denoted in different colors: yellow for alpha-helices, red for beta sheets, and blue for loops.

Protein structure analyses revealed all the positively selected sites were located at the surface of the NA protein and pertained to antibody binding and/or interactions with the sugar molecules of host cells (Figure 5, Figures S6, S7, S8, S9, S10, S11, S12, S13, S14, S15, S16). In addition, a number of positively selected sites reside in regions of the NA protein where neuraminidase inhibitors have been known to bind, indicating strong selection in influenza viruses with molecular markers predictive of antiviral resistance.

In the human H1N1 lineage (1C), amino acid positions 151, 222 and 344 were found to be under a strong positive selection, and the amino acids in these appear to interact with the NA inhibitor – zanamivir, a drug molecule according to the NA structure (Figure 5-A). In addition, positively selected sites 344 and 365 are located in the B-cell antigenic regions. The amino acid position 319 in human H1N1 lineage, identified to be under positive selection, forms a hydrogen bond with position 379, whose backbone carbonyl is involved in interactions with calcium ions (Figure 5-A). This Ca^2+^ ion interacts with positions 379, 389, 387, 382, and 381, forming H-bonds with position 385 and position 383. These interactions are crucial in protein folding to create the appropriate tertiary structure for sialic acid binding (which allows the NA to cleave the sialic acid) or for NA inhibitor binding.

With regard to another human lineage (2B), positions 126 and 127 were found to be within the binding pocket of influenza A virus (Figure 5-B). These two residues, along with residues 120 and 151 were found to be under positive selection. All these sites fold in close proximity to each other, providing a hydrogen-bond network that is essential for NA inhibitor binding. Specifically, position 151 forms a hydrogen bond to position 75, which itself is predicted to bind to zanamivir.

For human influenza B, positions 42, 65, 248, 345, 373, 389, 395, and 436 were found to be under positive selection (Table 3). The crystal structure of the B/Perth/211/2011 virus NA region with zanamivir, oseltamivir, or peramivir showed that residues 373 and 374 participated in drug binding, while residue 345 is involved in calcium binding and dimerization of two NA monomers (Figure 5-C, D).

## Discussion

### Evolution of Influenza Viral NA Genes - Types, Subtypes and Lineages

**Figure 6:**
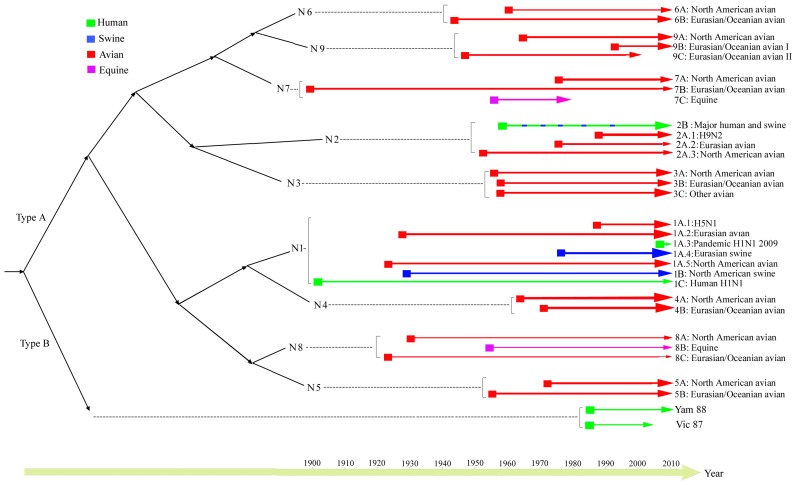
The evolutionary dynamics of influenza neuraminidase (NA) over time. The lineages from different hosts are colored, with the emergence times of the lineages represented by the horizontal positions of squared boxes and the mean substitution rates depicted by the degree of line thickness. Note that within 2A there are five swine clusters.

The ML and Bayesian MCMC analyses revealed that the divergence of influenza A and B NA genes occurred earlier than the divergence of influenza A NA subtypes. Similar findings were reported for the hemagglutinin (HA) genes [27], in which influenza A and B HA genes were found to diverge first, followed by the division of influenza A HA subtypes. Interestingly, within influenza A, both subgroups (I and II) consist mainly of human, swine, avian, and equine viruses and show similar patterns of host-specific lineage composition (Figure 6). This strongly supports the hypothesis that subgroup I and II viruses experienced parallel evolution due to similar rates of genetic mutation and adaption to host environments [2], [7].

In this study, 23 NA lineages were determined within influenza A based upon both theoretical (e.g., phylogenetic tree topology) and empirical criteria (e.g., pandemic events). The majority of lineages were found to be specific in hosts, or geographical locations, with a genetic distance around 0.2, ranging from 0.117 to 0.349. These results are generally consistent with previous findings [2], [28], [29], but our study relies on a much larger dataset (focusing on the NA segment) and illustrates more detailed evolutionary dynamics of the influenza A NA lineages.

Classification and designation of the lineages and sublineages within the influenza A virus are essential for studies of viral evolution, ecology and epidemiology. However, how to accurately identify an evolutionary lineage of influenza A viruses is challenging. Whether the naming system will be accepted and used by influenza researchers is even more challenging. To trace the evolutionary change of highly pathogenic avian influenza (HPAI) viruses, a hierarchical nomenclature system for HPAI hemagglutinin clades and sub-clades has been implemented by the WHO/OIE/FAO H5N1 Evolution Working Group and widely adapted by the research community [30]. The work presented here is one of the first steps toward the development of a nomenclature system for influenza A virus lineages (at the segment level) and genotypes (at the genome level). We will incorporate the findings of lineages and genotypes into our FluGenome database for the detection of newly emerging viral lineages and genome reassortment, which will improve influenza surveillance [31].

### Substitution Rate Heterogeneity within Influenza NA Lineages

It is notable that substitution rates are not the same across all branches within a phylogenetic tree. The relaxed clock model was developed to cope with this issue. An average rate across all branches in the tree is estimated under relaxed clock model in BEAST with 95% HPDs summarized from average rates, which are estimated from the sampled trees [32]. The 95% HPDs thus reflect the topological uncertainty among the sampled trees, but do not show the rate variation across different branches within a tree. In previous studies, the relaxed clock model was used to estimate the substitution rate and 95% HPDs and the resulting values were used for comparison [15], [23], [25], [26]. In fact, such comparison is less accurate. For example, if a phylogenetic tree is mixed with branches of very high and low rates, it might result in an average rate that is similar to that from another tree with branches of a constant rate. We cannot simply conclude the two virus lineages evolve at the same rate. Using the random local clock (RLC), we not only computed the mean substitution rate and 95% HPDs but also estimated rate heterogeneity among different branches of a phylogenetic tree, which reflects evolutionary dynamics of influenza viruses with a given lineage.

### Evolutionary Dynamics of Human Influenza NA Lineages

This study demonstrated that human influenza viruses were shown to have little geographical restriction, indicating that human viruses were transmitted globally and probably rapidly as well [29]. In addition, this study provides new insights into the emergence time of the human pandemic influenza virus because of the employment of the new random local clock model. This model takes into account the rate variation among different branches within lineage and has been considered superior to other models. For the H1N1 human lineage (1C), which includes the sequences from the 1918 Spanish Flu, the tMRCA under the random local clock model was estimated to be 1898 (95% HPD: 1882–1909), which is earlier than the years previously reported. Using the uncorrelated exponential relaxed clock model, the tMRCA of pandemic 1918 H1N1 viruses was estimated to be 1905–1918 [33] and 1910–1915 [22].

Lineage 2B includes human influenza viruses isolated from two different subtypes, H2N2 between 1957 and 1968 and H3N2 after 1968, which share the same N2 gene maintained in human influenza virus after the antigenic shift from H2 to H3 occurred in 1968 [34]. The tMRCA of lineage 2B was estimated to be 1956, which is six years later than the tMRCA of 1950 estimated by Smith et al. (2009a), but further supporting the hypothesis that emerging lineages circulate years prior to their initial detection in humans. Furthermore, the human lineages have relatively high non-synonymous to synonymous (*d*_N_/*d*_S_) ratios (1C: 0.263, 2B: 0.313, Yam88: 0.259, and Vic87: 0.257), suggesting strong immune selection in viruses persistently circulating in humans [9].

In addition to the above discussed human lineages, pandemic H1N1 2009 influenza viruses are believed to have arisen from a reassortment between North American and Eurasian swine lineages, and as expected, the pandemic H1N1 2009 viruses grouped with the Eurasian swine lineage [35], [36], [37]. The substitution rate for NA genes of pandemic H1N1 2009, estimated using sequences from the entire pandemic period (as of March 2011), was found to be very close to the substitution rate estimated in the early outbreak period [35]. In addition, the *d*_N_ */d*_S_ ratio of the NA genes of pandemic H1N1 2009 was 0.226, which was higher than the ratio of the closely related swine NA genes (0.100) [35]. This increase could be attributed either to the adaptations of pandemic H1N1 2009 viruses to humans or to intensive sampling (more frequent mutations detected) [35]. Immune pressure from a previously infected and/or vaccinated population may also account for the differences observed.

### Evolutionary Dynamics of Avian Influenza NA Lineages

Influenza viruses circulating in non-human species have evolved in association with their various hosts on different continents for extended periods of time [38]. Avian influenza viruses were usually classified into Eurasian and North American lineages in the past, which was attributed to confinement of birds to distinct flyways in each hemisphere [39], [40].This phylo-geographic pattern is evident in the lineages designated for N4–N9 subtypes (Figure 6). We also found that the viruses isolated from Oceania were grouped together with Eurasian viruses. Therefore, we expanded our geographic designation and used Eurasian/Oceanian to define theses lineages. Avian lineages were found in all nine subtypes, whereas the mammalian lineages occurred in only four subtypes, providing support for the hypothesis that wild aquatic avian viruses are considered the natural reservoir of all influenza viruses (Figure 6) [2], [41]. In addition, the avian lineages from N3 to N9 appeared to be under strong purifying selection pressures as suggested by the low *d*_N_ */d*_S_ ratios. Similar observations were also described in a previous study [42].

The two subtype-specific avian sublineages, 1A.1 for H5N1 and 2A.1 for H9N2, are considered to have pandemic potential and were found to evolve relatively faster compared with other avian lineages from multiple subtypes (Table 2). In addition, these two sublineages were found to be relatively young (23 years for H9N2 and 17 years for H5N1), indicating a more recent emergence likely indicative of their adaptation from a wild bird reservoir to domestic poultry and their ensuing establishment in poultry populations. Together, the higher substitution rates and contemporary divergence of sublineages, 1A.1 (H5N1) and 2A.1 (H9N2) may be attributable to the rapid geographical dissemination of these viruses in wild birds and poultry, followed by the establishment of endemicity in poultry-dense regions and consistent transmission and outbreaks [25], [43]. It will be interesting in future studies to determine if sampling biases (over-sampling in the case of H5N1 and H9N2) may also play a role in the higher substitution rates and recent divergence times observed compared to other avian influenza virus lineages. Nonetheless, it is important to continue systematic surveillance of these high-risk viruses in both birds and humans to better understand these processes.

### Evolutionary Dynamics of Equine Influenza NA Lineages

Two lineages, H7N7 (7C) and H3N8 (8B), were revealed in equine influenza viruses. The H7N7 equine influenza viruses have not been detected since the late 1970s [44], whereas H3N8 was first isolated in 1963 [45] and is still circulating in equine populations throughout most of the world. Lineage 8B (H3N8) is composed predominantly of equine viruses, but canine influenza viruses are also found in this lineage. This observation is consistent with the fact that equine influenza virus has crossed the species barrier and become established as a respiratory pathogen of dogs [46]. The equine influenza viruses share ancestors with avian viruses in the same subtype, indicating their possible avian origin.

### Evolutionary Dynamics of Swine Influenza NA Lineages

Two major swine virus groups, Eurasian (avian-like) swine (1A.4) and North American swine (1B), were found within N1 (Figure 6). Our observation of geographic separation of swine lineages agrees with previous findings [15]. Our tMRCA analysis of the Eurasian (avian-like) swine lineage revealed that an avian virus crossed the species boundary from birds to pigs in 1978, which is seven years later than previously described [15] but just one year earlier than the first detection of these viruses in 1979. Similar *d*_N_ */d*_S_ ratios were observed in both swine lineages, suggesting comparable selection pressures occurred in both lineages [35]. Interestingly, amino acid positions 46, 53, and 453 in North American swine (1B), which were found to be under positive selection, are located in the T-cell antigenic regions, while position 339 lies in the B-cell antigenic region [47], indicating a strong immune selection occurred on these positions.

Complicated evolutionary dynamics were observed in lineage 2B. Within this major human lineage, five separate sub-clusters of swine viruses occurred in North America and Eurasia, suggesting that human-origin N2 genes were transmitted to swine in at least five separate instances (Figures 2-B and 6). Between the 1970s and 1980s, human-origin H3N2 influenza viruses circulated in Eurasia [48], [49]. Reassortment events between human-origin H3N2 and avian-like H1N1 swine influenza virus resulted in the emergence of H3N2 viruses, with HA and NA from human viruses and all six internal genes originally from birds [50]. These viruses eventually superseded the former human-origin H3N2 viruses in swine around 1984. In 1994, a further swine reassortant H1N2 virus was identified in the United Kingdom [51]. Phylogenetic analyses revealed that the HA gene of this virus was derived from a human-like H1N1 virus, whereas the NA and internal genes were derived from the European swine reassortant H3N2 [28].

In addition to the complexity found in Eurasian swine N2 viruses, similarly, in North America in 1998 there were outbreaks of influenza observed in swine herds in Minnesota, Iowa, and Texas. The outbreaks were caused by a triple-reassortant H3N2 virus which contained genes from human (HA, NA, and PB1), swine (NS, NP, and M), and avian (PB2 and PA) influenza viruses [52]. An additional important reassortment event in North American swine resulted in an H1N2 reassortant between classical H1N1 swine (contributing only HA) and H3N2 swine (contributing the other seven segments) [37]. These reassortment events, coupled with interspecies transmission between swine and humans, have led to the complexity seen within lineage 2A.

**In summary**, we analyzed 14,328 influenza A and B NA sequences and studied the evolutionary history and phylodynamics of the influenza NA gene. The divergence of influenza NA into influenza A and B NA occurred first, and nine NA subtypes further diverged within influenza A, with two to three lineages identified within each NA subtype. The analyses of substitution rates, *d*_N_ */d*_S_ ratio, selection sites and protein structures revealed important associations between mutations and antiviral drug resistance/vaccine escape. Further analyses of other influenza segments are needed in order to obtain a comprehensive understanding of influenza virus evolution, which will facilitate influenza surveillance and control.

## Materials and Methods

### Sequence Data

A total of 14,328 neuraminidase (NA) nucleotide sequences longer than 1330 nts, excluding laboratory recombinant sequences, were downloaded from the Influenza Virus Resource at NCBI [53]. Their host distributions are detailed in Table 4. A Perl script (http://sysbio.harvard.edu) was used to remove identical sequences, which resulted in 10,679 NA sequences, including 10,001 influenza A and 678 influenza B sequences. The influenza A NA sequences were divided into nine datasets (one for each subtype) that consist of 4146, 3754, 351, 85, 128, 488, 189, 684, and 176 sequences respectively for N1–N9.

**Table 4:** Host distribution of neuraminidase (NA) sequences in influenza A and B viruses.

Influenza	Subtype	Human	Avian	Swine	Equine	Others	Total
**A**	N1	3810	1853	243	0	50	5956
	N2	3378	1215	258	0	81	4932
	N3	1	412	4	0	26	443
	N4	0	121	0	0	2	123
	N5	0	141	0	0	0	141
	N6	0	583	3	0	24	610
	N7	0	219	1	11	4	235
	N8	0	568	2	118	95	783
	N9	0	192	0	0	2	194
**B**		911	0	0	0	0	911

### Recombination Test

Homologous gene recombination was identified using the 3SEQ algorithm under RDP3 [54]. Ideally, all influenza sequences are analyzed in a single run. However, because of computational limitations to the program when a large data set is used, we examined our dataset for gene recombination within each influenza A subtype and within influenza B. Sequences with mosaic recombination signals were identified using a cutoff p-value 0.05 [55].

The SeqMat program was used to collapse similar sequences from the same location and the same year, which results in ∼1500 representative sequences, respectively, for N1 and N2 [56]. For other subtypes, we used all available sequences to detect recombination. Fourteen influenza A N1, 14 N2, two N3, one N4, five N6, three N8, and one influenza B NA sequences were identified to have mosaic recombination signals and thus were excluded from the analyses. No mosaic recombination signals were found in N5, N7 or N9.

### Sequence Alignment and Phylogenetic Analysis

Influenza A and B NA sequences are remotely related with around 40% nucleotide sequence similarity. We thus conducted both protein and nucleotide sequence alignments using Expresso - a program based upon protein structural information for alignment and TranslatorX - a program referring to the corresponding protein sequence alignment to align nucleotide sequences, respectively [57]–[58]. The resulting alignment between influenza A and B sequences was considered to be of good quality, which assured the reliability of the downstream analysis (File S2). MAFFT and MUSCLE were used to align sequences from each of the nine influenza A NA subtypes and the influenza B NA sequences, respectively [59], [60]. The alignment results from MAFFT and MUSCLE were compared and adjusted accordingly.

Phylogenetic analysis was conducted using the Maximum-likelihood (ML) method in RAxML [61]. RAxML uses rapid algorithms for bootstrap and maximum likelihood searches and is considered one of the fastest and most accurate phylogeny programs. Two hundred independent inferences starting from random MP trees were performed, and the tree with the highest likelihood score was selected as the representative. The GTRGAMMA model was employed to correct the biases of multiple substitution and rate heterogeneity in sequences. All the analyses were conducted on the supercomputing clusters at the Holland Computing Center (http://hcc.unl.edu/main/index.php). The trees were visualized and color-coded using FigTree (version 1.3.1) (http://tree.bio.ed.ac.uk/software/figtree/) to demonstrate tree topologies and corresponding hosts, subtypes and geographic locations.

### Identification of Lineage and Sublineage

Lineages were determined based upon the topology of phylogenetic trees and strong bootstrap support values (100 for influenza A and approximately 90 for influenza B). The genetics distances between lineages were calculated using the Kimura-2-Parameter (K2P) distance matric under MEGA 5.0 [62]. Additional information such as the distribution of viruses in hosts and geographic regions were also considered in the classification. The aim was to identify the lineages of clearly related sequences, which might interest the virology-epidemiology community and could be used for further evolutionary dynamics analyses. The lineage and sublineage were named according to the following notations: a single digit is used to represent one of the nine influenza A NA subtypes and a letter is used to represent a lineage. A sublineage is also represented using a digit. A dot is used to separate a lineage and a sublineage. For example, 1A.2 means N1 subtype, lineage A, and sublineage 2. For influenza B, two lineages were assigned and named following the conventions well-accepted by the influenza research community. To make our lineage assignment scheme justifiable and extensible, we use alphabetic letters to represent lineages in the order of avian, swine, human, and equine for hosts and in the order of the North America followed by Eurasian/Oceanian in geography.

### Substitution Rate and Time of Most Recent Common Ancestor (tMRCA)

The substitution rate and the time of most recent common ancestor (tMRCA) were estimated for each lineage/sublineage using the Bayesian Markov Chain Monte Carlo (MCMC) method available in the BEAST package [32]. Prior to the MCMC analysis, the linear regression and residual analyses for each lineage were performed using Path-O-Gen [63]; significant outliers identified were then removed. To reduce excessive computational load, we followed the common strategy that achieves computer tractability while preserving the accuracy of the estimates [64]. We wrote a Java program to select around 120 sequences from each lineage or sublineage, which represent viruses sampled from different locations and at different time points. In all cases, the data were analyzed under the GTR (General Time Reversible) + 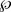 nucleotide substitution model, as this model was consistently found to be the best by Modeltest [65].

Three clock models were compared statistically for each dataset using a Bayes factor test in the Tracer program [66]: a strict clock, an uncorrelated lognormal relaxed clock (UCLD) and an uncorrelated exponential relaxed clock (UCED) [67]. The UCED model was found to provide the best fit for all lineages. In addition, we used the newly developed random local clock model (RLC) that takes into account the rate variation among different branches within lineage by applying a series of local molecular clocks, each extending over a subregion of the overall phylogeny. All estimates also incorporated a different substitution rate for each codon position and a Bayesian skyline coalescent prior [68]. For each dataset, two independent Bayesian MCMC runs were conducted for 30 million generations to achieve convergence, with uncertainty in parameter estimates reflected in the 95% highest probability density (HPD). The Maximum Clade Credibility (MCC) tree across all plausible trees was then computed from the BEAST trees using the TreeAnnotator program, with the first 10% of trees removed as burn-in.

### Measurement of Selection Pressures

The ratio of non-synonymous (*d*_N_) to synonymous (*d*_S_) substitutions per site (ratio *d*_N_ */d*_S_) were estimated using the single likelihood ancestor counting (SLAC) method available in the HYPHY package [69]. Positively selected codons were detected using the single likelihood ancestor counting (SLAC), fixed effects likelihood (FEL) and internal fixed effects likelihood (IFEL) methods with a significance level of 0.05. In the SLAC method, the nucleotide and codon model parameter estimates are used to reconstruct the ancestral codon sequences at the internal nodes of the tree. The single most likely ancestral sequences are then fixed as known variables, and applied to infer the expected number of non-synonymous or synonymous substitutions that have occurred along each branch, for each codon position. The FEL method is based on maximum-likelihood estimates. The FEL method estimates the ratio of non-synonymous to synonymous substitutions on a site-by-site basis for the entire tree or only the interior branches (IFEL). In all cases, *d*_N_/d_S_ estimates were based on Maximum-likelihood trees under the GTR + Г substitution model. Protein structures of template NAs used in structural analyses were downloaded from the Protein Data Bank (www.pdb.org). Positively selected sites were mapped on the structure of the protein using Molecular Operating Environment (MOE) [70].

## Supporting Information

Figure S1 **Maximum-likelihood (ML) tree of influenza A N3 genes.** Three lineages, denoted 3A, 3B and 3C, were classified. The bootstrap values supporting the corresponding lineages are shown on the major nodes. The scale bars indicate the numbers of nucleotide substitutions per site.
(TIF)

Figure S2 **Maximum-likelihood (ML) tree of influenza A N4 genes.** Two lineages, denoted 4A and 4B, were classified. The bootstrap values supporting the corresponding lineages are shown on the major nodes. The scale bars indicate the numbers of nucleotide substitutions per site.
(TIF)

Figure S3 **Maximum-likelihood (ML) tree of influenza A N6 genes.** Two lineages, denoted 6A and 6B, were classified. The bootstrap values supporting the corresponding lineages are shown on the major nodes. The scale bars indicate the number of nucleotide substitutions per site.
(TIF)

Figure S4 **Maximum-likelihood (ML) tree of influenza A N7 genes.** Three lineages, denoted 7A, 7B and 7C, were classified. The bootstrap values supporting the corresponding lineages are shown on the major nodes. The scale bars indicate the number of nucleotide substitutions per site.
(TIF)

Figure S5 **Maximum-likelihood (ML) tree of influenza A N9 genes.** Three lineages, denoted 9A, 9B and 9C, were classified. The bootstrap values supporting the corresponding lineages are shown on the major nodes. The scale bars indicate the number of nucleotide substitutions per site.
(TIF)

Figure S6 **The structure of 1A.1 influenza neuraminidase, with positive selection sites denoted as green balls.**
(TIF)

Figure S7 **The structure of 1A.2** **influenza neuraminidase, with positive selection sites denoted as green balls.**
(TIF)

Figure S8 **The structure of 1A.3 influenza neuraminidase, with positive selection sites denoted as green balls.**
(TIF)

Figure S9 **The structure of 1A.4 influenza neuraminidase, with positive selection sites denoted as green balls.**
(TIF)

Figure S10 **The structure of 1A.5 influenza neuraminidase, with positive selection sites denoted as green balls.**
(TIF)

Figure S11 **The structure of 1B influenza neuraminidase, with positive selection sites denoted as green balls.**
(TIF)

Figure S12 **The structure of 2A.1 influenza neuraminidase, with positive selection sites denoted as green balls.**
(TIF)

Figure S13 **The structure of 2A.3 influenza neuraminidase, with positive selection sites denoted as green balls.**
(TIF)

Figure S14 **The structure of 5A influenza neuraminidase, with positive selection sites denoted as green balls.**
(TIF)

Figure S15 **The structure of 6A influenza neuraminidase, with positive selection sites denoted as green balls.**
(TIF)

Figure S16 **The structure of 8A influenza neuraminidase, with positive selection sites denoted as green balls.**
(TIF)

Table S1 **The number of sequences of each lineage and the number of outliers identified by residual analysis.**
(DOCX)

File S1 **The phylogenetic tree of influenza A and B neuraminidase sequences.**
(TREE)

File S2 **The alignment of influenza A and B neuraminidase sequences.** The quality of the alignment is indicated by different colors.
(DOCX)
