# Supplementary figures and images for: Evolutionary History and Phylodynamics of Influenza A and B Neuraminidase (NA) Genes Inferred from Large-Scale Sequence Analyses
Source: PLoS One. 2012 Jul 11;7(7):e38665. doi: 10.1371/journal.pone.0038665 (PMC3394769; doi:10.1371/journal.pone.0038665)

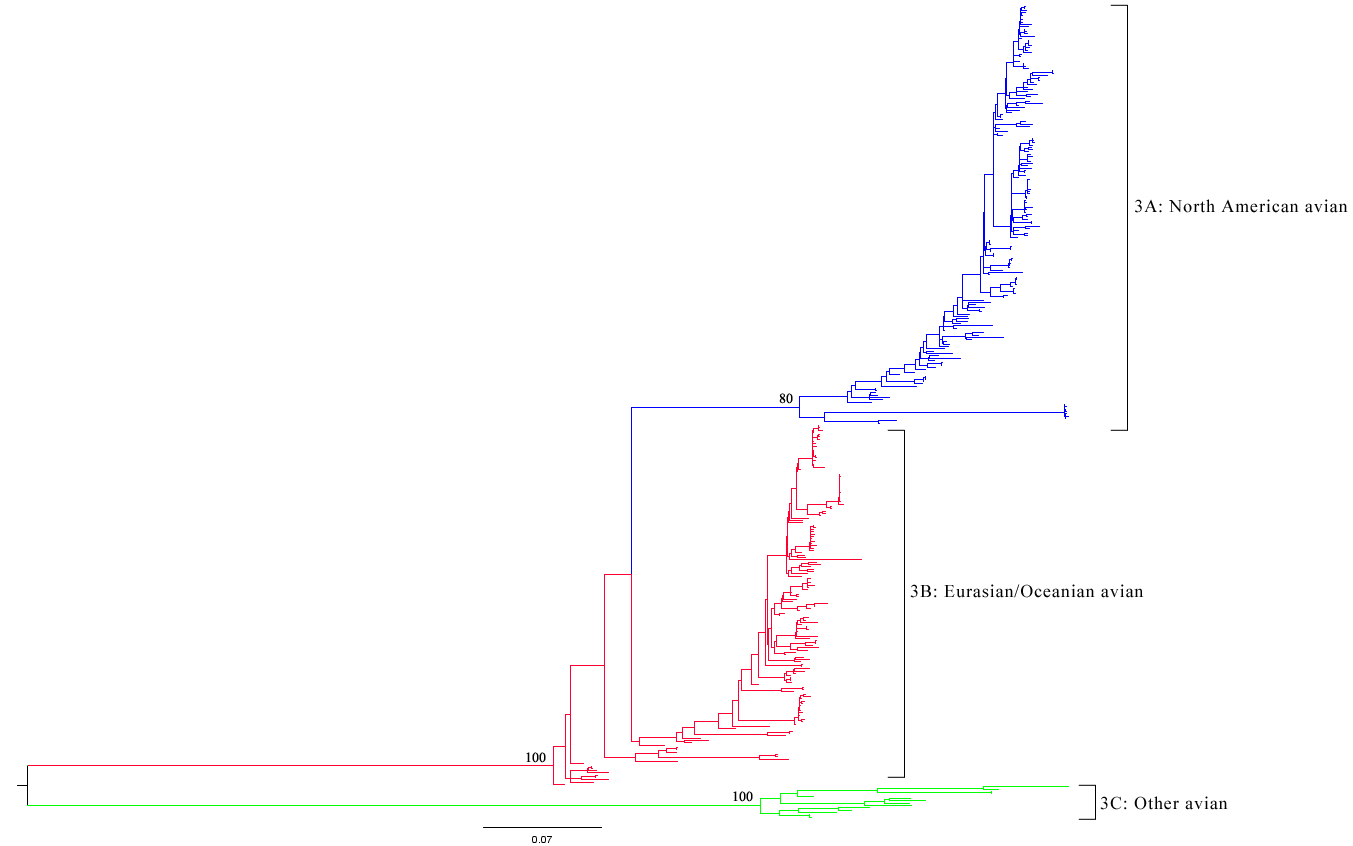

Supplement: Figure S1 — Maximum-likelihood (ML) tree of influenza A N3 genes. Three lineages, denoted 3A, 3B and 3C, were classified. The bootstrap values supporting the corresponding lineages are shown on the major nodes. The scale bars indicate the numbers of nucleotide substitutions per site. (TIF) [file pone.0038665.s001.tif]

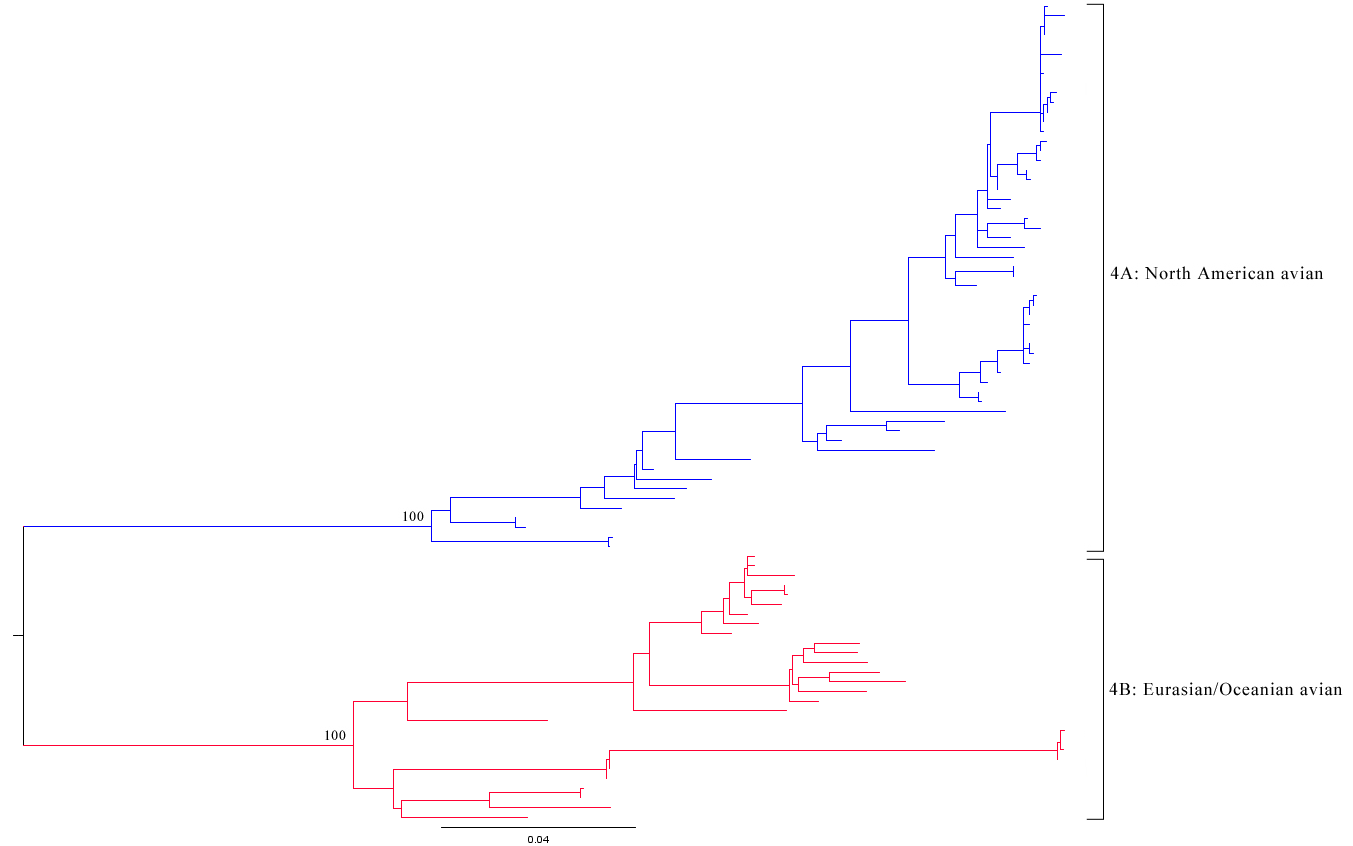

Supplement: Figure S2 — Maximum-likelihood (ML) tree of influenza A N4 genes. Two lineages, denoted 4A and 4B, were classified. The bootstrap values supporting the corresponding lineages are shown on the major nodes. The scale bars indicate the numbers of nucleotide substitutions per site. (TIF) [file pone.0038665.s002.tif]

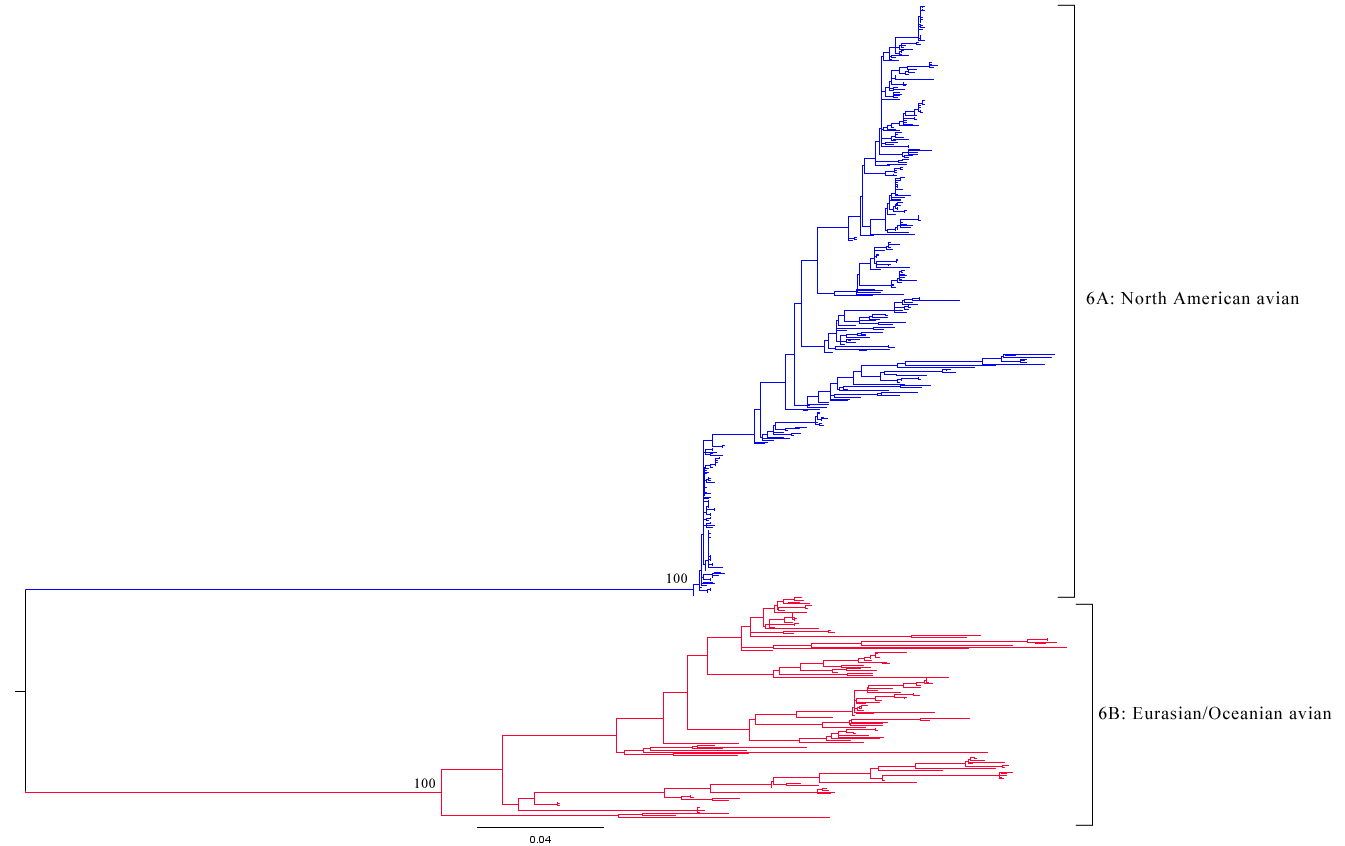

Supplement: Figure S3 — Maximum-likelihood (ML) tree of influenza A N6 genes. Two lineages, denoted 6A and 6B, were classified. The bootstrap values supporting the corresponding lineages are shown on the major nodes. The scale bars indicate the number of nucleotide substitutions per site. (TIF) [file pone.0038665.s003.tif]

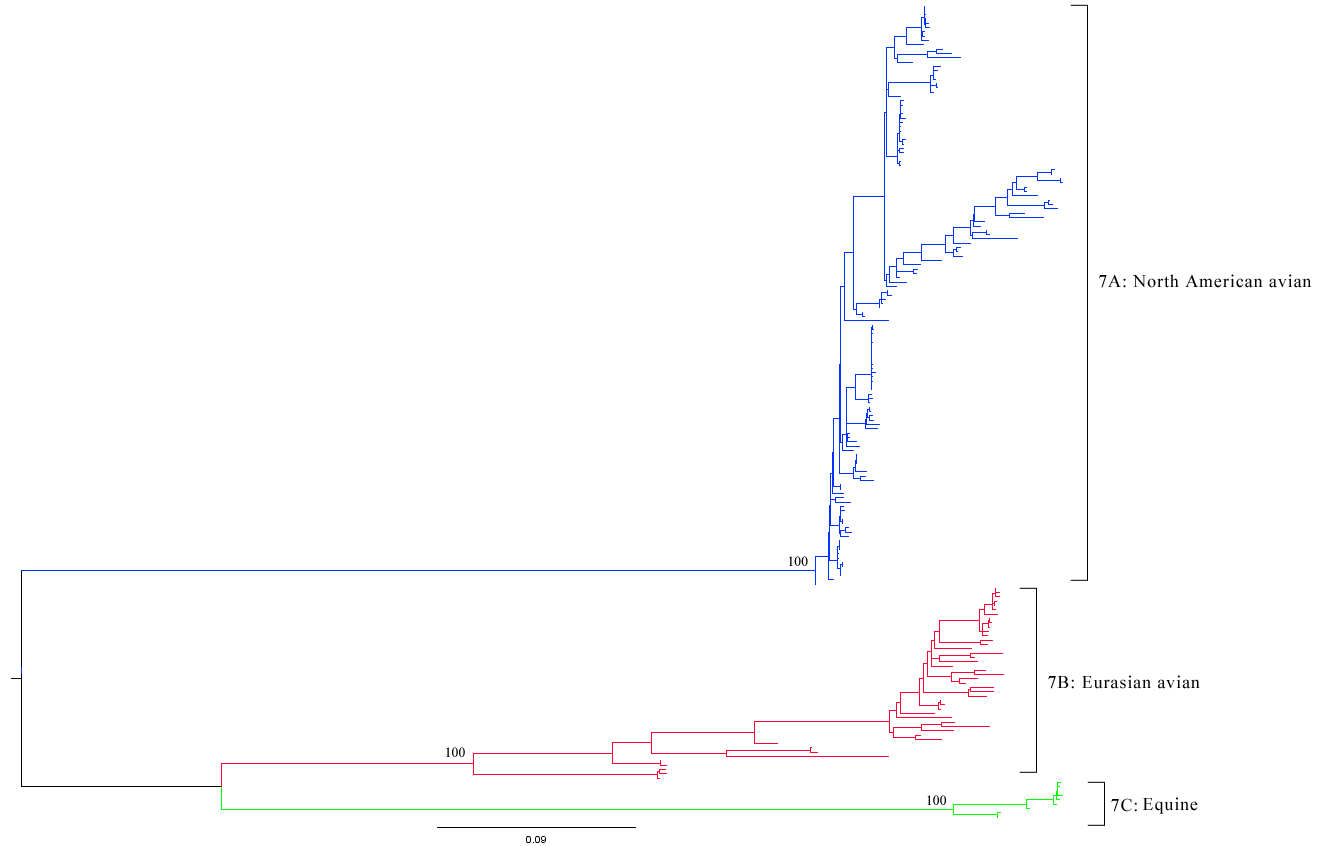

Supplement: Figure S4 — Maximum-likelihood (ML) tree of influenza A N7 genes. Three lineages, denoted 7A, 7B and 7C, were classified. The bootstrap values supporting the corresponding lineages are shown on the major nodes. The scale bars indicate the number of nucleotide substitutions per site. (TIF) [file pone.0038665.s004.tif]

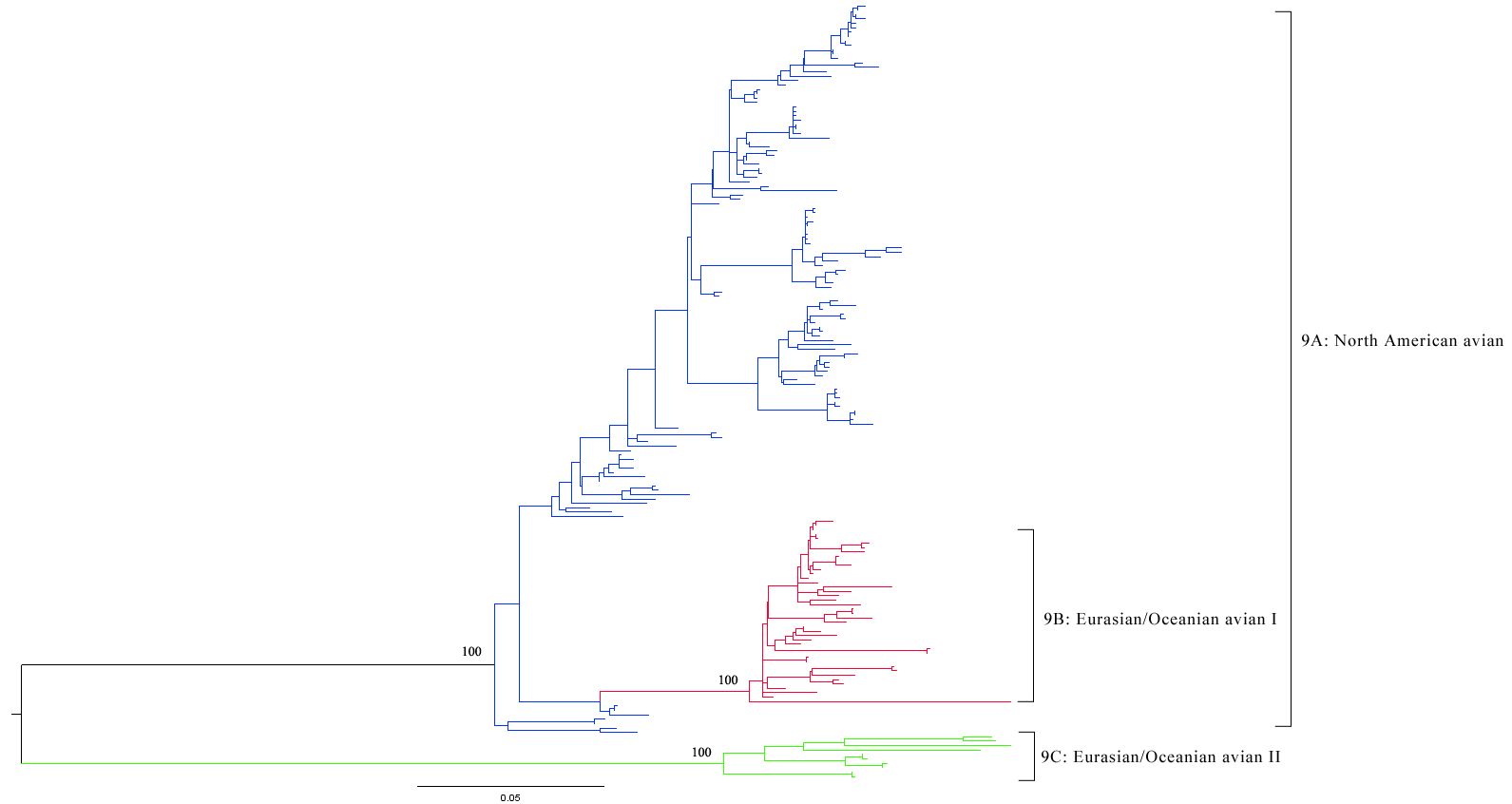

Supplement: Figure S5 — Maximum-likelihood (ML) tree of influenza A N9 genes. Three lineages, denoted 9A, 9B and 9C, were classified. The bootstrap values supporting the corresponding lineages are shown on the major nodes. The scale bars indicate the number of nucleotide substitutions per site. (TIF) [file pone.0038665.s005.tif]

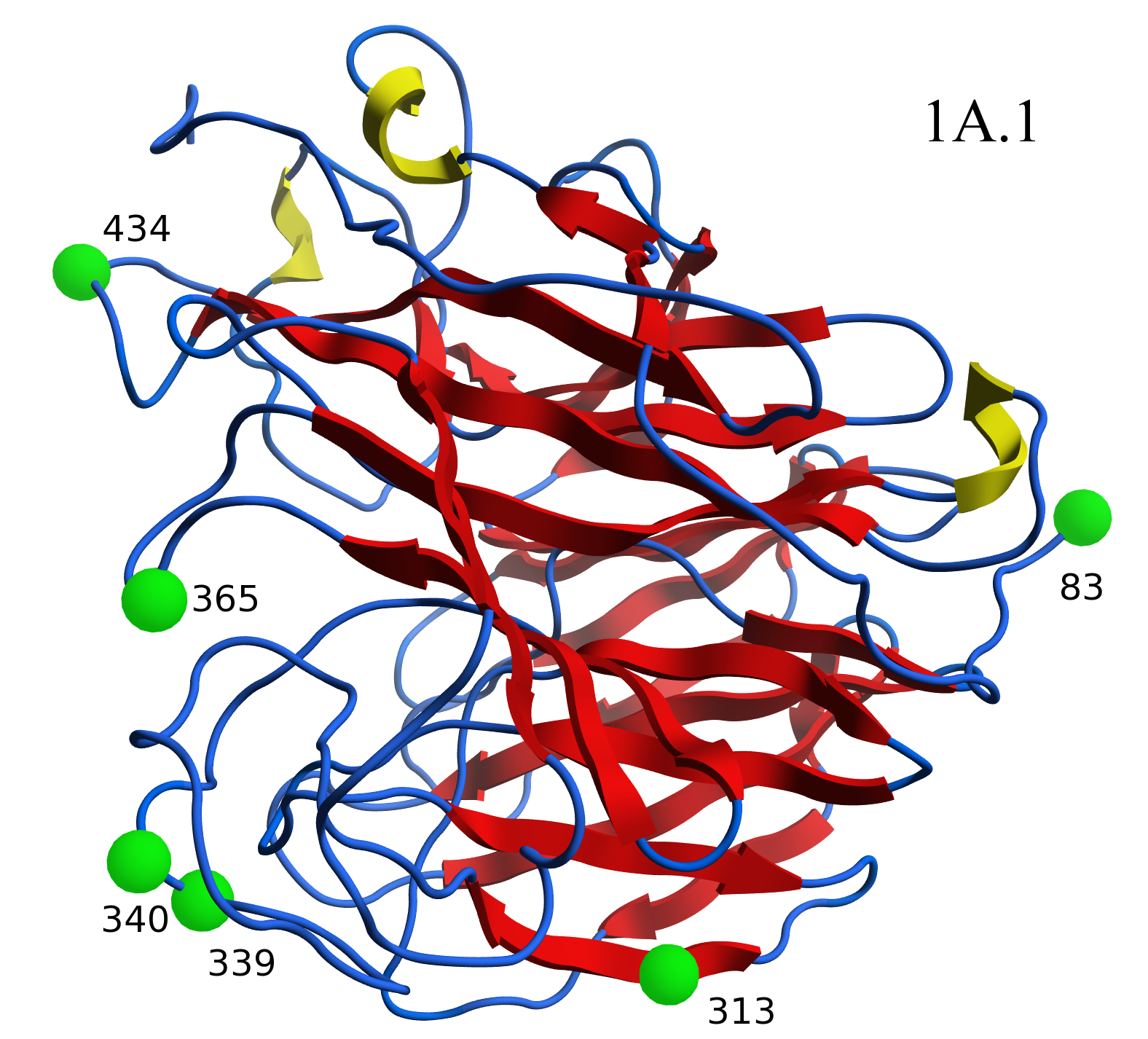

Supplement: Figure S6 — The structure of 1A.1 influenza neuraminidase, with positive selection sites denoted as green balls. (TIF) [file pone.0038665.s006.tif]

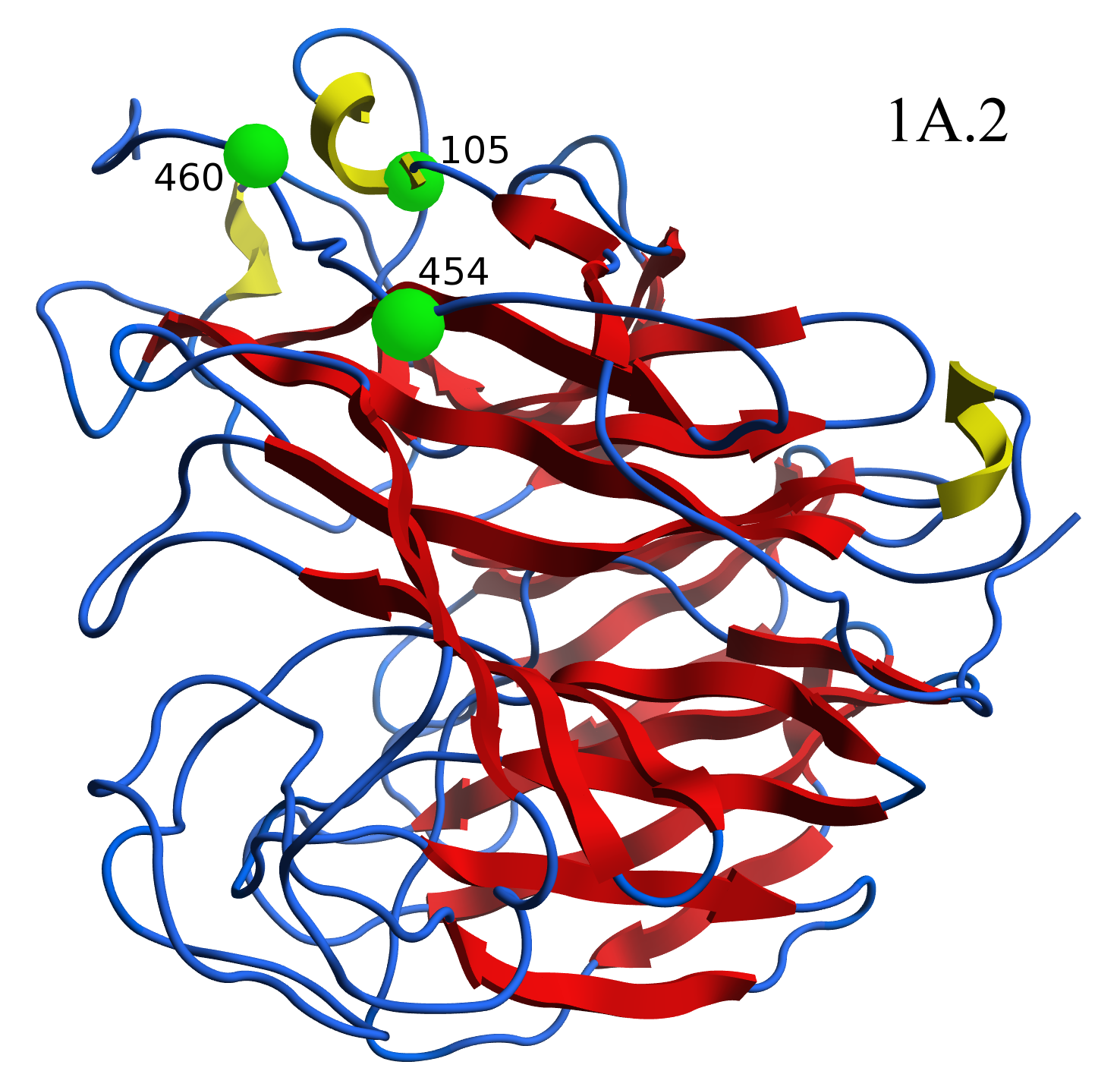

Supplement: Figure S7 — The structure of 1A.2 influenza neuraminidase, with positive selection sites denoted as green balls. (TIF) [file pone.0038665.s007.tif]

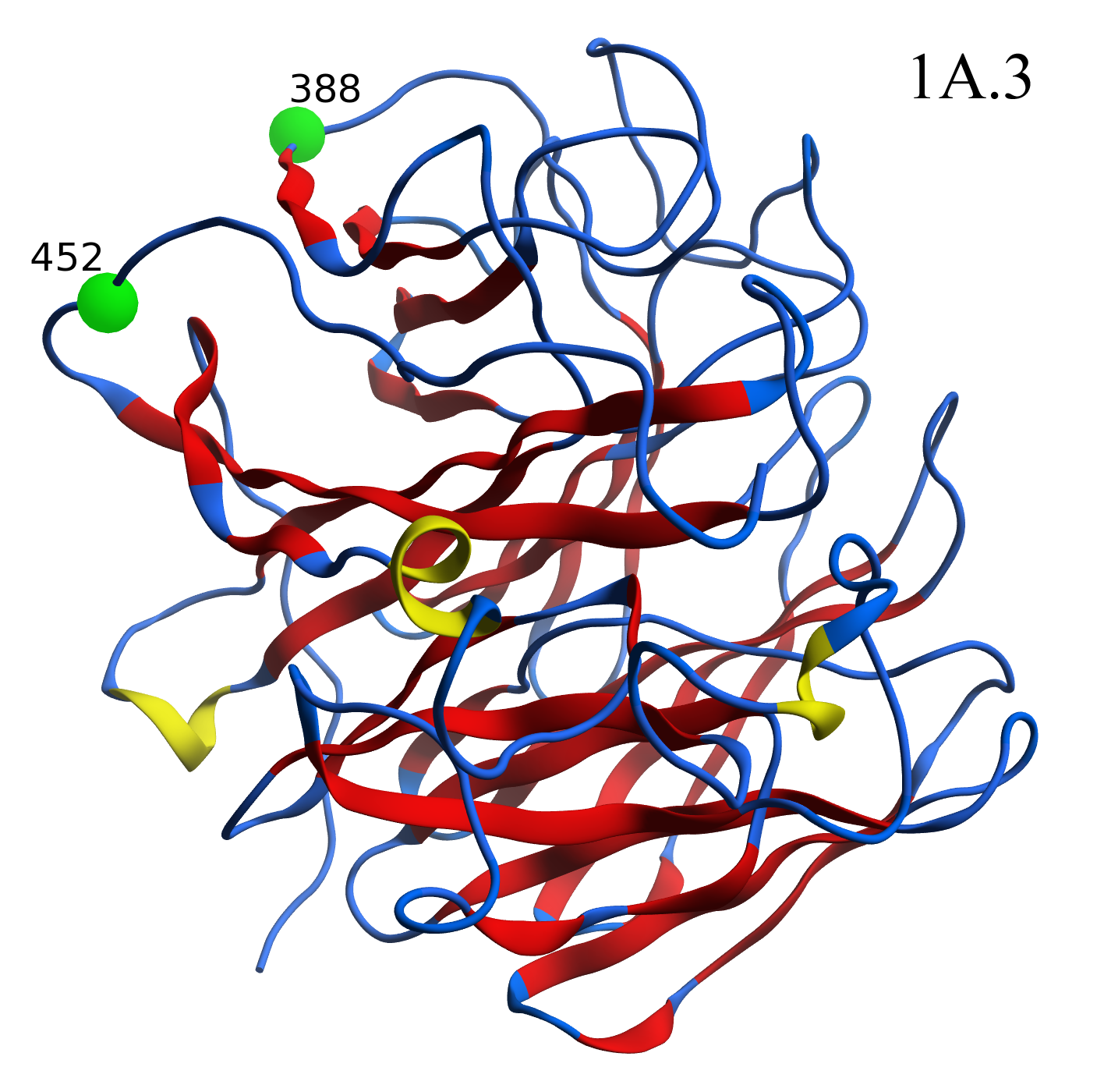

Supplement: Figure S8 — The structure of 1A.3 influenza neuraminidase, with positive selection sites denoted as green balls. (TIF) [file pone.0038665.s008.tif]

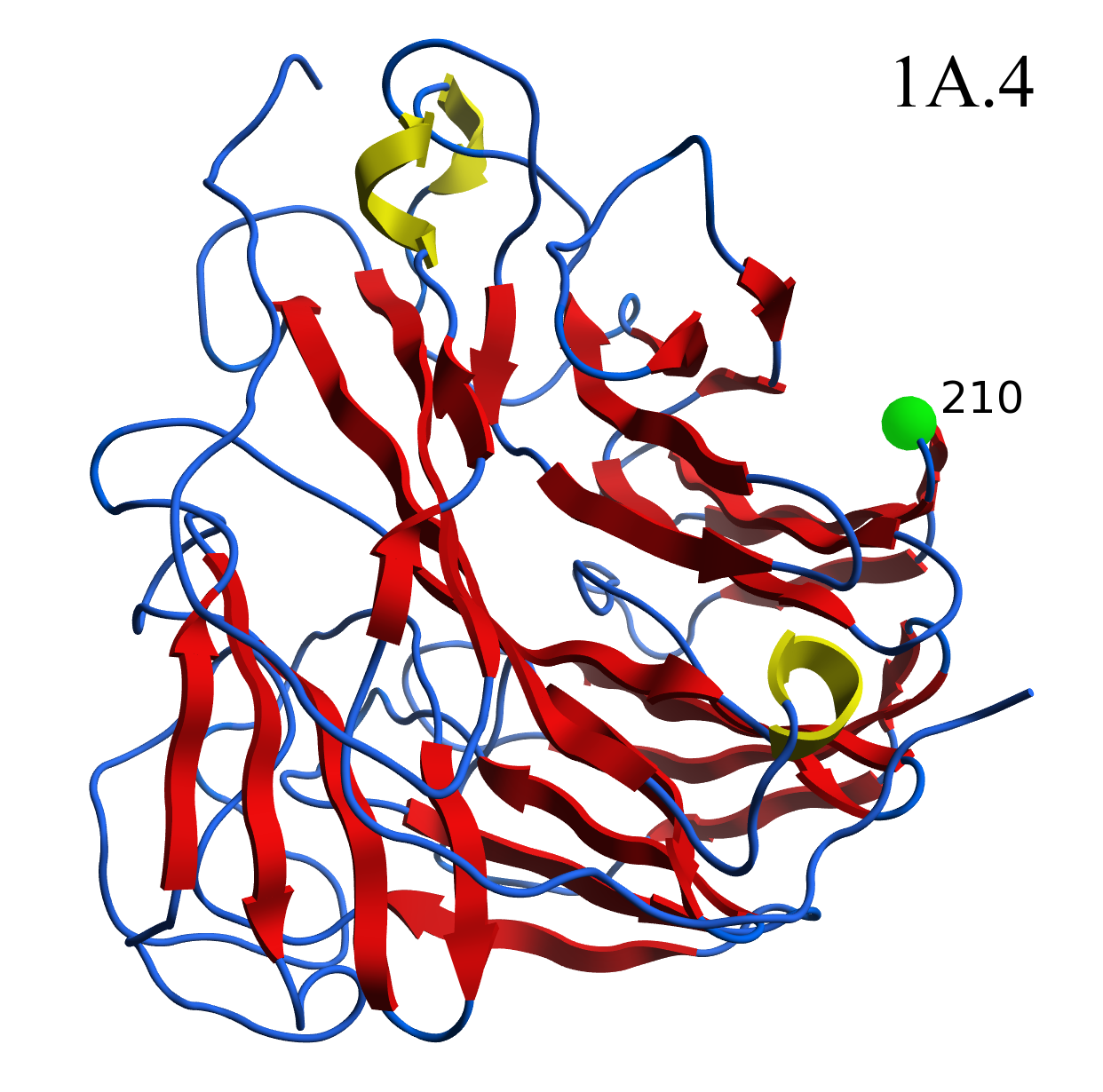

Supplement: Figure S9 — The structure of 1A.4 influenza neuraminidase, with positive selection sites denoted as green balls. (TIF) [file pone.0038665.s009.tif]

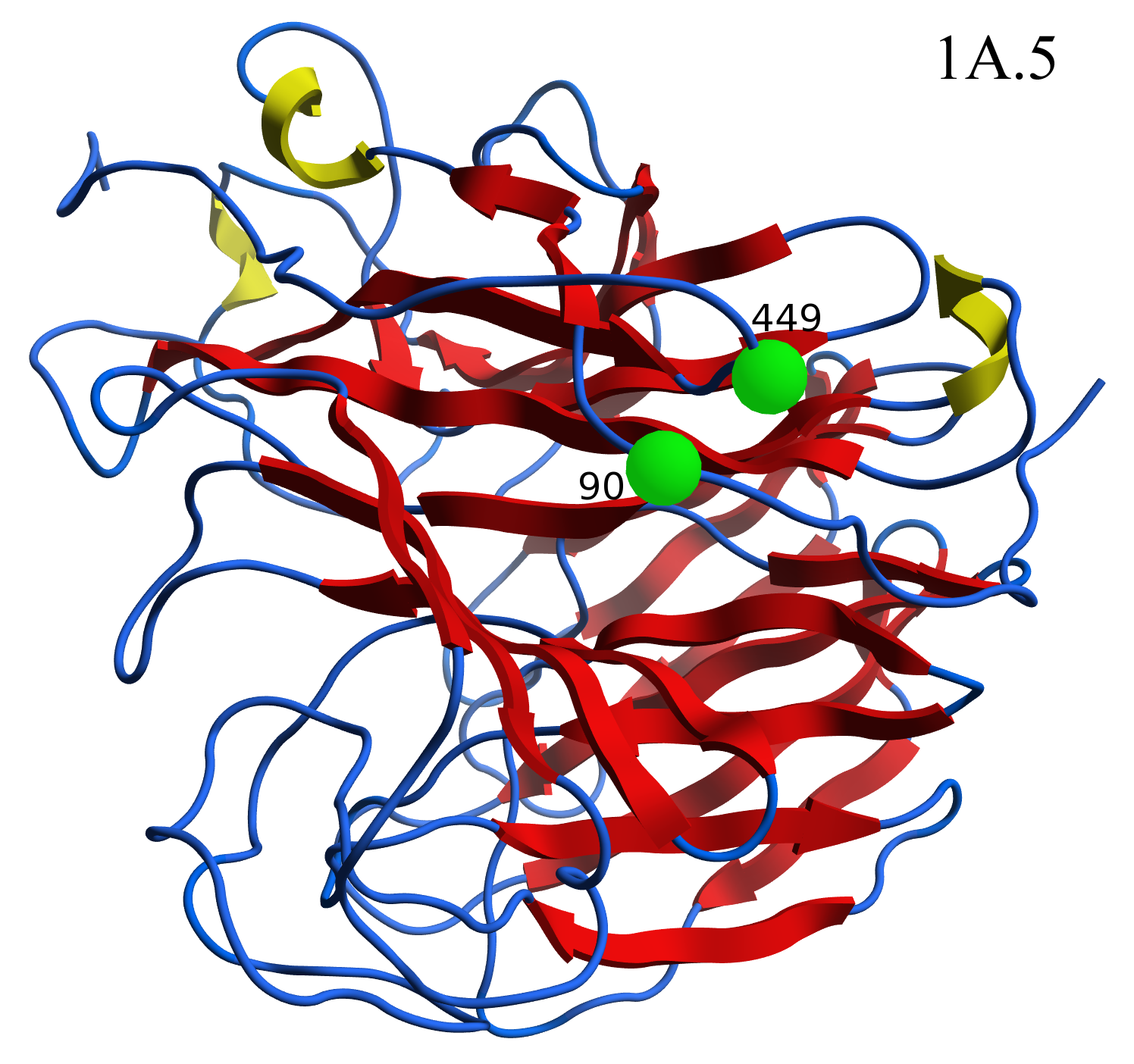

Supplement: Figure S10 — The structure of 1A.5 influenza neuraminidase, with positive selection sites denoted as green balls. (TIF) [file pone.0038665.s010.tif]

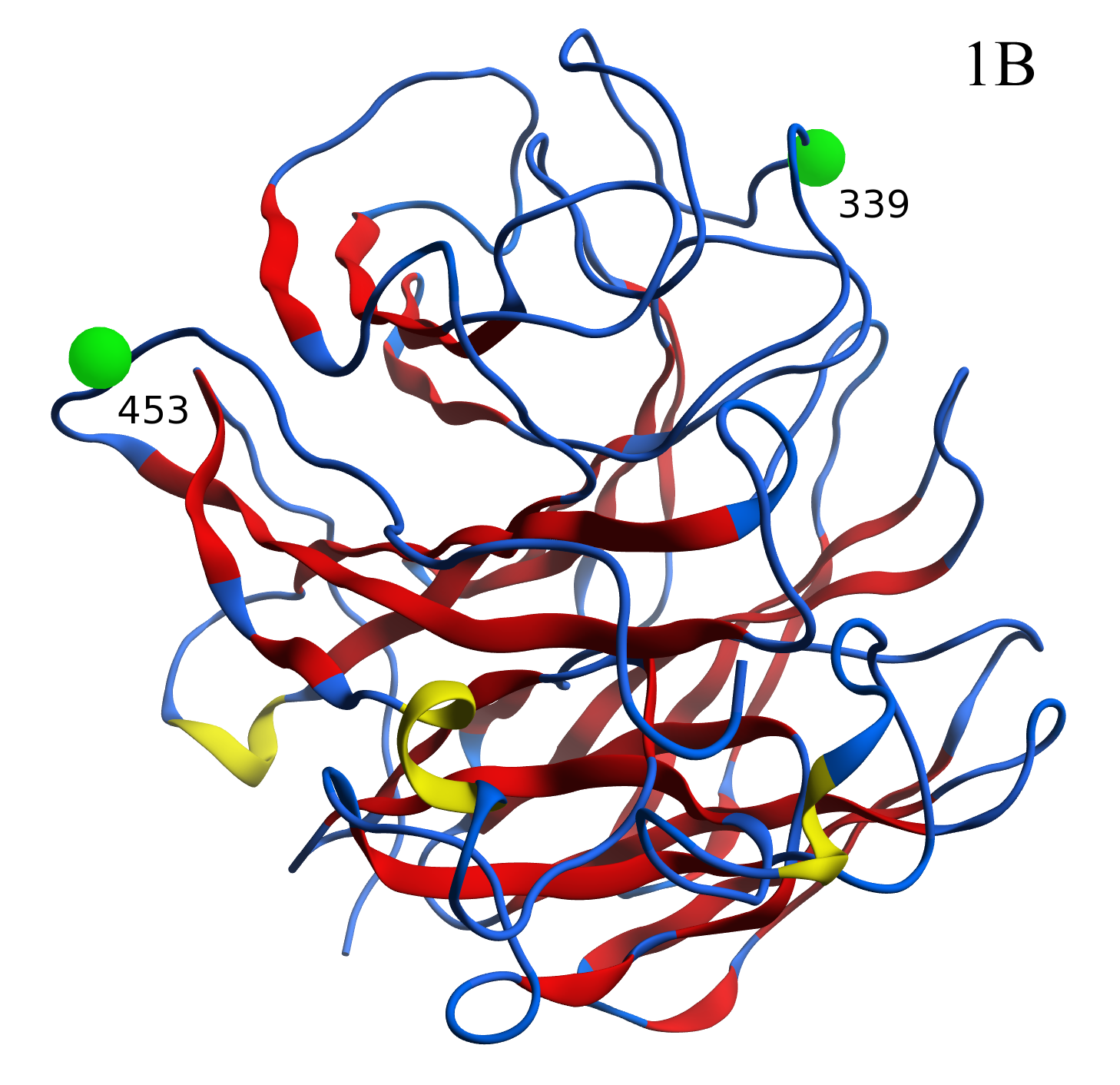

Supplement: Figure S11 — The structure of 1B influenza neuraminidase, with positive selection sites denoted as green balls. (TIF) [file pone.0038665.s011.tif]

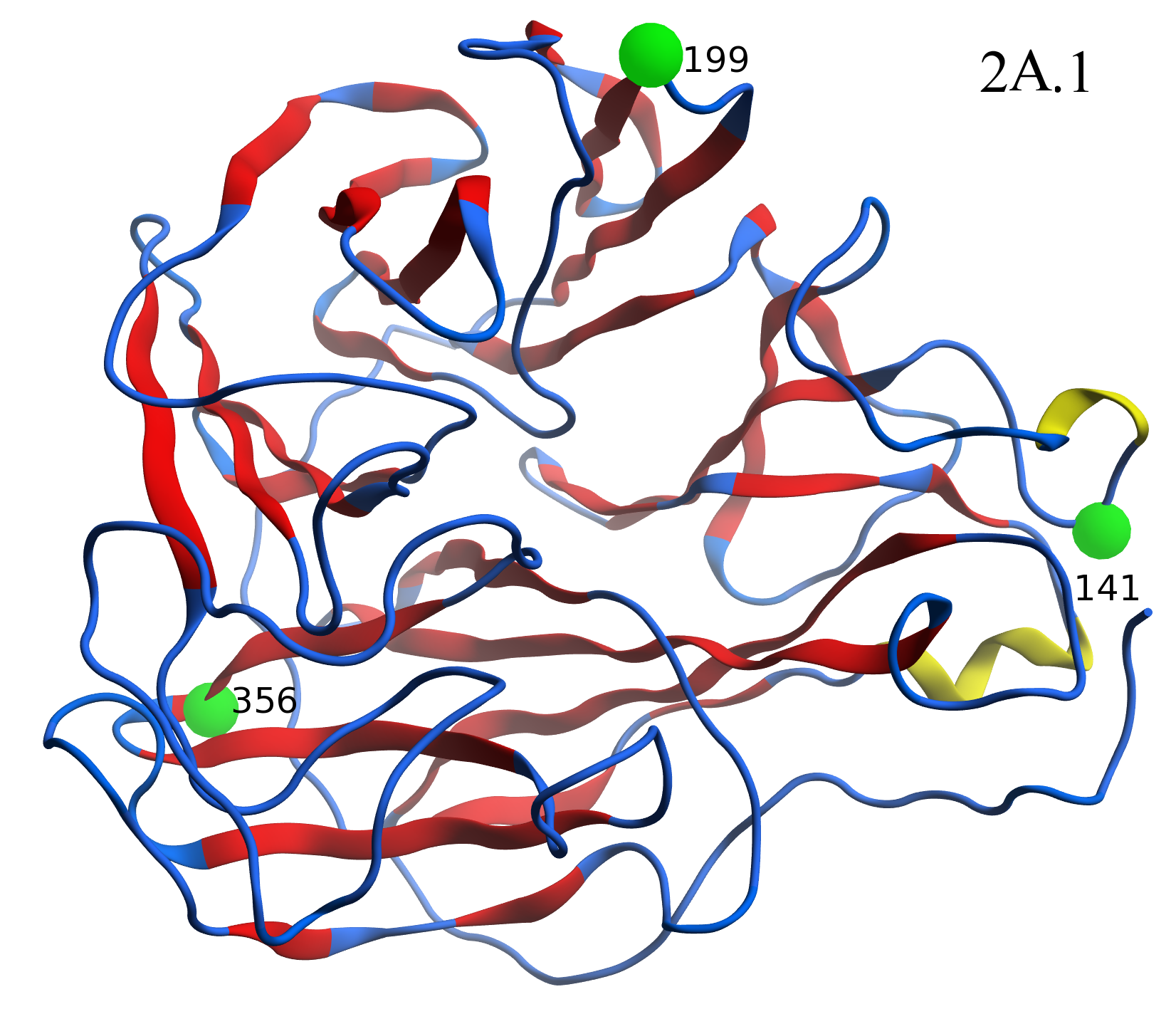

Supplement: Figure S12 — The structure of 2A.1 influenza neuraminidase, with positive selection sites denoted as green balls. (TIF) [file pone.0038665.s012.tif]

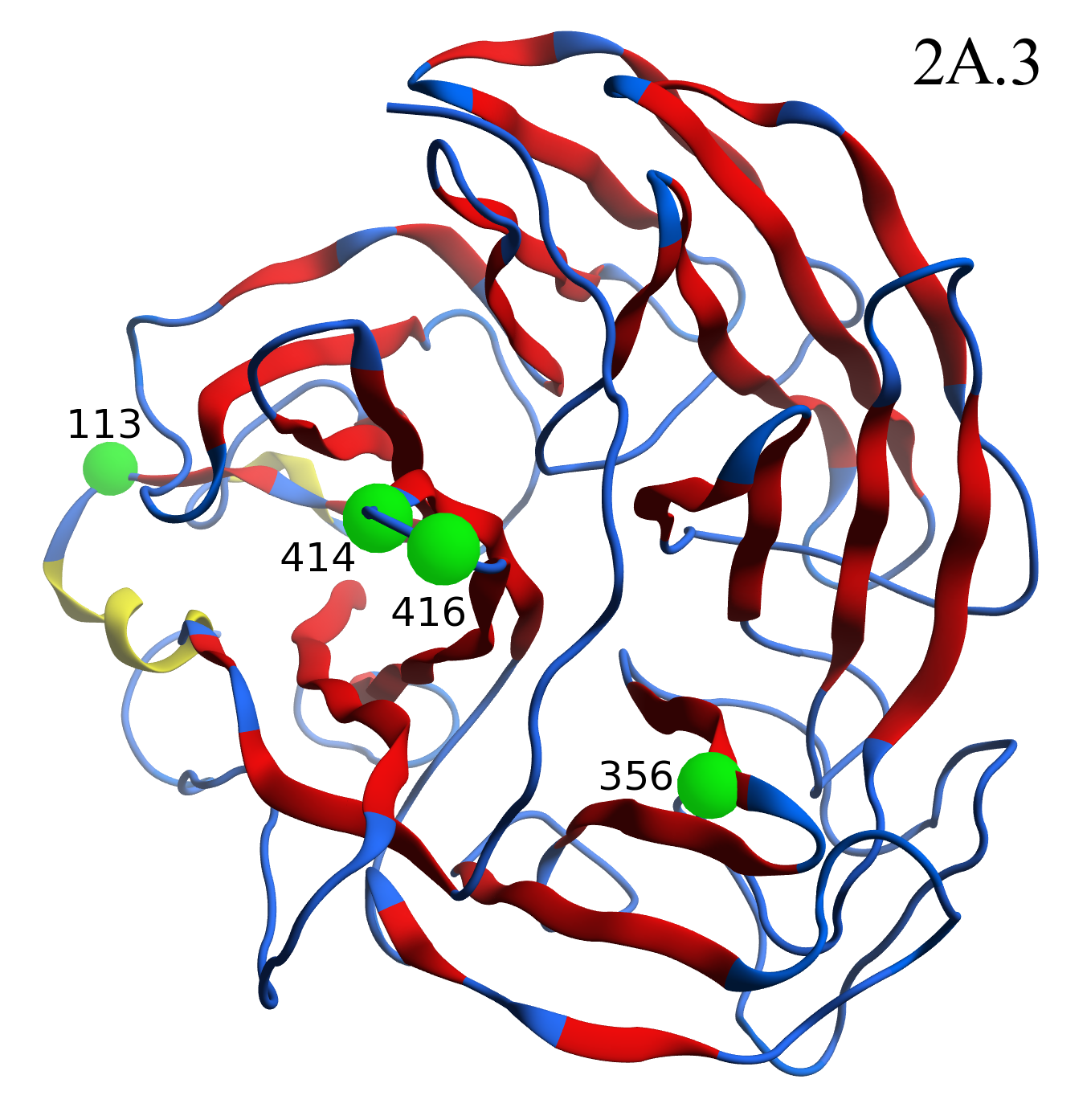

Supplement: Figure S13 — The structure of 2A.3 influenza neuraminidase, with positive selection sites denoted as green balls. (TIF) [file pone.0038665.s013.tif]

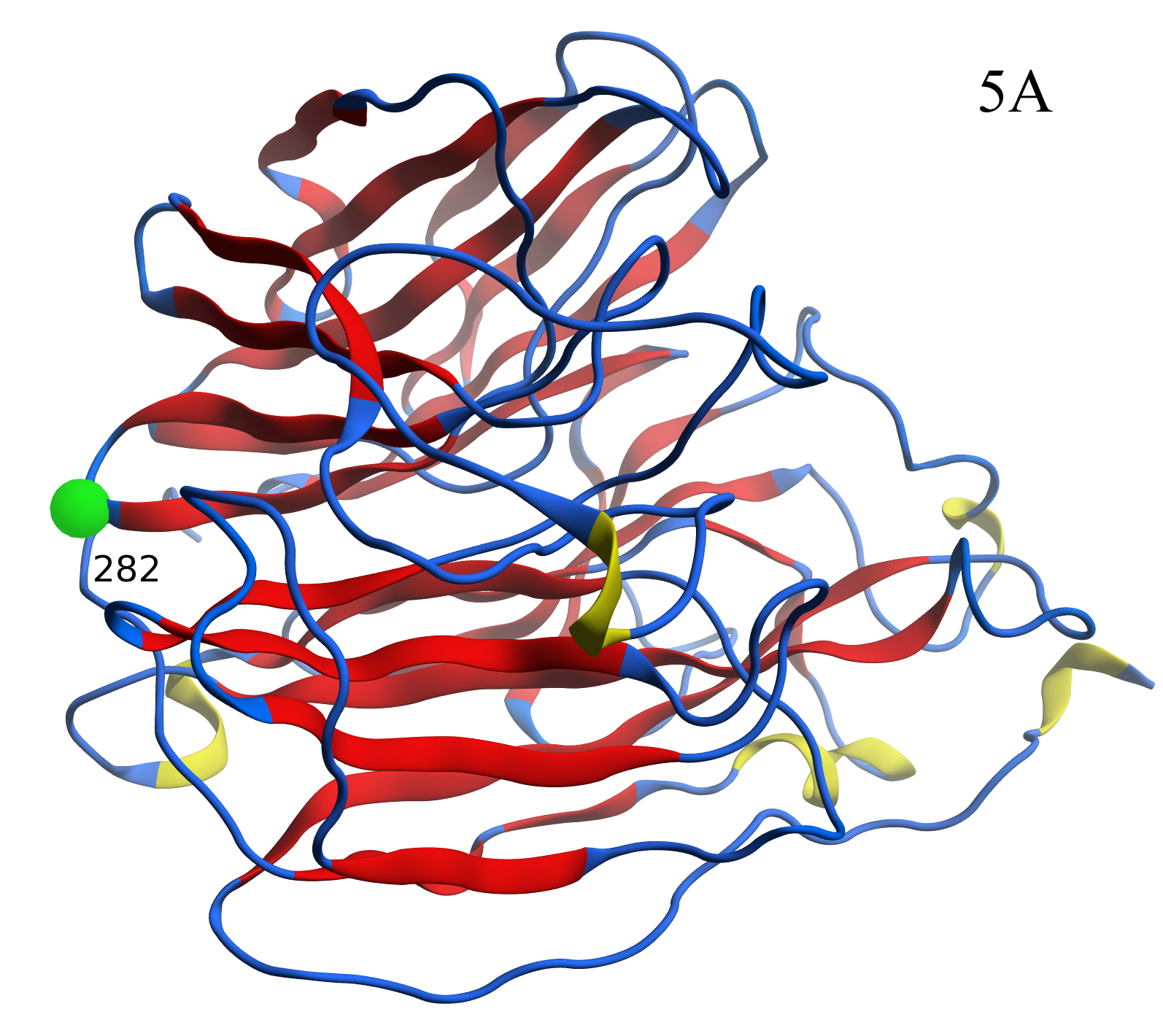

Supplement: Figure S14 — The structure of 5A influenza neuraminidase, with positive selection sites denoted as green balls. (TIF) [file pone.0038665.s014.tif]

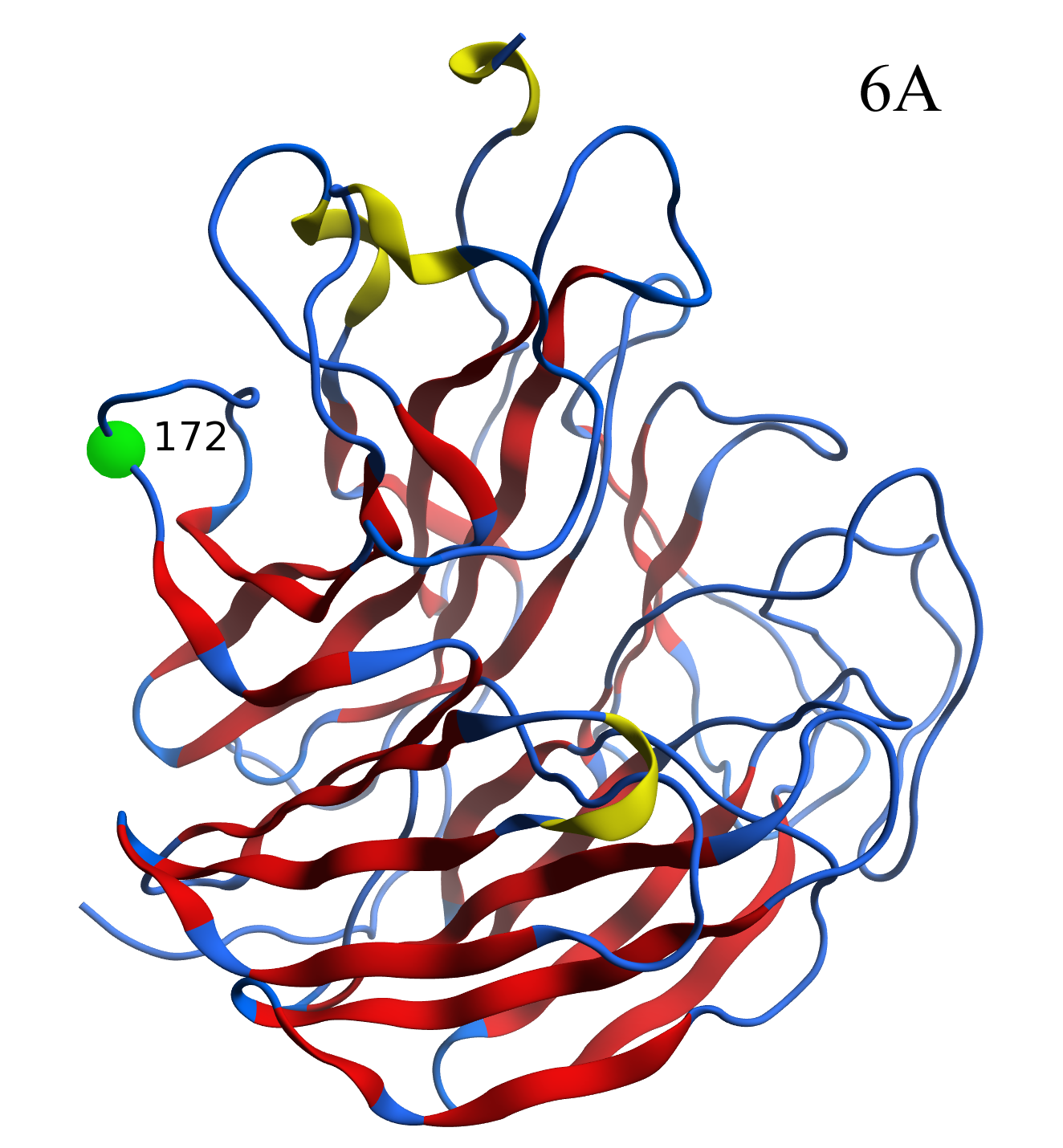

Supplement: Figure S15 — The structure of 6A influenza neuraminidase, with positive selection sites denoted as green balls. (TIF) [file pone.0038665.s015.tif]

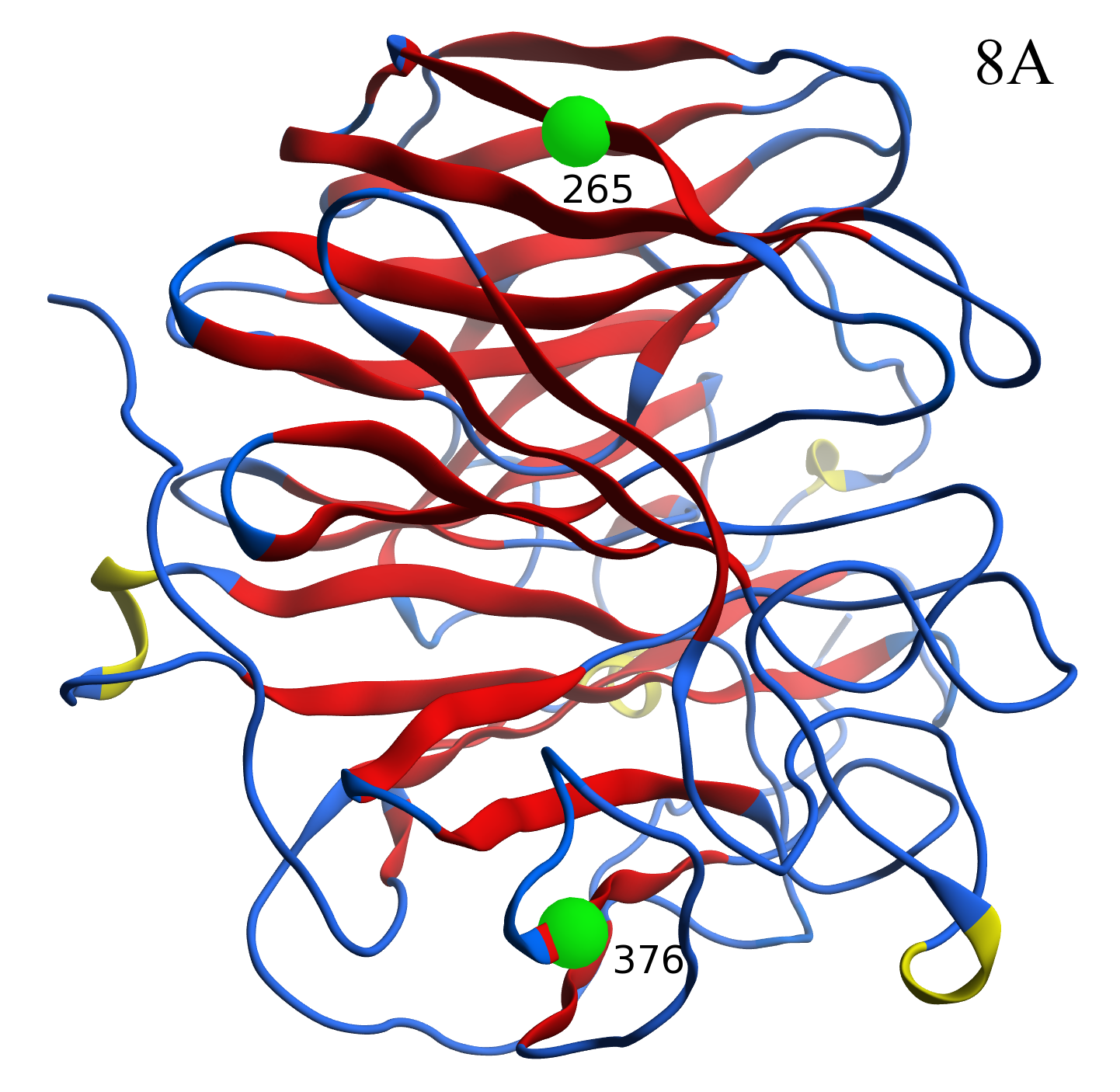

Supplement: Figure S16 — The structure of 8A influenza neuraminidase, with positive selection sites denoted as green balls. (TIF) [file pone.0038665.s016.tif]
