# Supplementary material for: Evolutionary History and Phylodynamics of Influenza A and B Neuraminidase (NA) Genes Inferred from Large-Scale Sequence Analyses
Source: PLoS One. 2012 Jul 11;7(7):e38665. doi: 10.1371/journal.pone.0038665 (PMC3394769; doi:10.1371/journal.pone.0038665)
Supplement: Table S1 — The number of sequences of each lineage and the number of outliers identified by residual analysis. (DOCX) [file pone.0038665.s017.docx]

**Table S1** The number of sequences of each lineage and the number of outliers identified by residual analysis

| Influenza | Subtype | Lineage | Annotation | Isolation period | N | Outliers |
| --- | --- | --- | --- | --- | --- | --- |
| A | N1 | 1A.1 | H5N1 | 1996-2010 | 1325 | 12 |
|  |  | 1A.2 | Eurasian avian | 1934-2009 | 258 | 0 |
|  |  | 1A.3 | Pandemic H1N1 2009 | 2009-2010 | 1850 | 0 |
|  |  | 1A.4 | Eurasian (avian-like) swine | 1979-2010 | 69 | 2 |
|  |  | 1A.5 | North American avian | 1969-2008 | 204 | 2 |
|  |  | 1B | North American swine | 1930-2009 | 146 | 4 |
|  |  | 1C | Major human | 1918-2010 | 1311 | 2 |
|  | N2 | 2A.1 | H9N2 | 1994-2009 | 583 | 16 |
|  |  | 2A.2 | Eurasian avian | 1977-2009 | 207 | 1 |
|  |  | 2A.3 | North American avian | 1972-2008 | 342 | 14 |
|  |  | 2B | Major human and swine | 1957-2009 | 2619 | 24 |
|  | N3 | 3A | North American avian | 1971-2008 | 181 | 0 |
|  |  | 3B | Eurasian/Oceanian avian | 1961-2009 | 156 | 0 |
|  |  | 3C | Other avian | 1980-2002 | 14 | 0 |
|  | N4 | 4A | North American avian | 1968-2008 | 57 | 0 |
|  |  | 4B | Eurasian/Oceanian avian | 1979-2006 | 28 | 0 |
|  | N5 | 5A | North American avian | 1976-2008 | 91 | 0 |
|  |  | 5B | Eurasian/Oceanian avian | 1972-2009 | 37 | 4 |
|  | N6 | 6A | North American avian | 1976-2008 | 355 | 0 |
|  |  | 6B | Eurasian/Oceanian avian | 1976-2007 | 133 | 6 |
|  | N7 | 7A | North American avian | 1977-2008 | 135 | 0 |
|  |  | 7B | Eurasian/Oceanian avian | 1927-2008 | 45 | 0 |
|  |  | 7C | Equine | 1956-1977 | 10 | 0 |
|  | N8 | 8A | North American avian | 1963-2010 | 430 | 11 |
|  |  | 8B | Equine | 1963-2010 | 101 | 0 |
|  |  | 8C | Eurasian/Oceanian avian | 1963-2010 | 91 | 8 |
|  | N9 | 9A | North American avian | 1966-2008 | 124 | 0 |
|  |  | 9B | Eurasian/Oceanian avian I | 1996-2010 | 42 | 1 |
|  |  | 9C | Eurasian/Oceanian avian II | 1978-2004 | 10 | 0 |
| B |  | Yam88 | - | 1988-2009 | 571 | 0 |
|  |  | Vic87 | - | 1987-2002 | 83 | 0 |
