# Supplementary material for: Evolutionary History and Phylodynamics of Influenza A and B Neuraminidase (NA) Genes Inferred from Large-Scale Sequence Analyses
Source: PLoS One. 2012 Jul 11;7(7):e38665. doi: 10.1371/journal.pone.0038665 (PMC3394769; doi:10.1371/journal.pone.0038665)
Supplement: File S2 — The alignment of influenza A and B neuraminidase sequences. The quality of the alignment is indicated by different colors. (DOCX) [file pone.0038665.s019.docx]

**File S2: The alignment of influenza A and B sequences. The quality of the alignment is indicated by different colors.**

T-COFFEE
SCORE=97
*
 BAD AVG GOOD
*
AAA43735/B:  94
AAA43743/B:  94
AAA43749/B:  94

AAU94779/B:  94

ABL84344/B:  94
ABO72374/B:  94

ABQ81846/B:  94

ACU12759/B:  94

BAB32609/B:  94

AAA43093/A:  97
AAA43429/A:  97
AAA43449/A:  97
AAA43575/A:  97
AAD49005/A:  97
AAD49007/A:  97
AAF77036/A:  97
AAO46232/A:  97
AAO46822/A:  97
AAO62036/A:  96
AAO62041/A:  97
AAO62044/A:  97
AAO62064/A:  97
AAT08005/A:  97
AAT37407/A:  96
AAT37414/A:  95
AAT65406/A:  96
AAX78821/A:  97
AAY52584/A:  97
AAY87411/A:  97
ABB17695/A:  97
ABB19439/A:  97
ABB19610/A:  97
ABB19747/A:  97
ABB19914/A:  97
ABB20088/A:  97
ABB20104/A:  97
ABB20243/A:  97
ABB20375/A:  97
ABB20475/A:  97
ABB20501/A:  97
ABB21754/A:  97
ABB87198/A:  97
ABB87732/A:  97
ABB87959/A:  97
ABB88058/A:  96
ABB88351/A:  97
ABC02269/A:  97
ABF47958/A:  97
ABG37189/A:  97
ABH04381/A:  96
ABI30359/A:  97
ABI84399/A:  95
ABI84436/A:  97
ABI84475/A:  97
ABI84973/A:  96
ABI84982/A:  97
ABI92217/A:  96
ABJ09099/A:  96
ABK32099/A:  97
ABP49198/A:  97
ABP49253/A:  97
ABQ97208/A:  97
ABR28683/A:  97
ABR37723/A:  97
ABS50124/A:  97
ABS89357/A:  97
ABU95382/A:  97
ABV25648/A:  97
ABV29593/A:  97
ABV82576/A:  97
ABV82587/A:  97
ABW95951/A:  97
ABX88802/A:  97
ABY84687/A:  97
ACA04655/A:  97
ACA04738/A:  97
ACA04739/A:  97
ACA04740/A:  97
ACA14299/A:  97
ACB70583/A:  97
ACD47117/A:  97
ACD65204/A:  97
ACD76864/A:  97
ACD85421/A:  97
ACE73320/A:  97
ACE76594/A:  97
ACE78976/A:  97
ACE78981/A:  97
ACF20219/A:  97
ACF25054/A:  97
ACF25354/A:  97
ACF33655/A:  96
ACF36500/A:  97
ACF93490/A:  97
ACI41114/A:  96
ACI48786/A:  97
ACN65661/A:  97
ACO24985/A:  97
ACP50710/A:  97
ACQ73415/A:  97
ACR66907/A:  96
ACR83968/A:  97
ACS68311/A:  97
ACS92897/A:  97
ACT67761/A:  97
ACT97062/A:  97
ACV41710/A:  97
ADA71169/A:  97
ADK70163/A:  97
ADU17627/A:  97
ADU20276/A:  97
ADU20397/A:  96
ADU53220/A:  97
AEB89856/A:  97
AEB89867/A:  97
AEK50939/A:  96
AEK50961/A:  97
BAA06132/A:  97
BAA06718/A:  97
BAF03532/A:  96
BAF03569/A:  97
BAF34378/A:  97
BAF34925/A:  97
BAF43417/A:  97
BAF43457/A:  97
BAF43461/A:  97
BAF46763/A:  96
BAF46905/A:  97
BAF48640/A:  97
BAF48644/A:  96
BAF48646/A:  97
BAF63047/A:  97
BAG66251/A:  97
BAG66259/A:  92
BAG66279/A:  97
BAH02172/A:  97
BAH70320/A:  97
BAI39637/A:  97
BAI48894/A:  97
BAI50030/A:  96
BAJ07979/A:  97
BAJ10562/A:  97
CAA36475/A:  97
CAC36997/A:  97
CAD57252/A:  97
CAO82694/A:  97
CAP69845/A:  97
cons      :  97

AAA43735/B      85 -------L-----------------SCQGSTFQ   93 
AAA43743/B      85 -------L-----------------SCQGSTFQ   93 
AAA43749/B      85 -------L-----------------SCQGSTFQ   93

AAU94779/B      85 -------L-----------------SCPGSTFQ   93

ABL84344/B      85 -------L-----------------SCPGSTFQ   93 
ABO72374/B      85 -------L-----------------SCPGSTFQ   93

ABQ81846/B      85 -------L-----------------SCQGSTFQ   93

ACU12759/B      85 -------L-----------------SCPGSTFQ   93

BAB32609/B      85 -------L-----------------SCQGSTFQ   93     
AAA43093/A      84 ----LNKS--------------L---CNVEGWV   95 
AAA43429/A      87 ------EP-----------------LCEAQGFA   96 
AAA43449/A      89 ------SS-----------------LCPIRGWA   98 
AAA43575/A      90 ------KG--------------L---CTINSWH   99 
AAD49005/A      89 ------KP-----------------QCQIKGFA   98 
AAD49007/A      89 ------KP-----------------QCQITGFA   98 
AAF77036/A      89 ------SS-----------------LCPISGWA   98 
AAO46232/A      89 ------KP-----------------QCQITGFA   98 
AAO46822/A      89 ------SS-----------------LCPISGWA   98 
AAO62036/A      87 ----PSLP--------------L---CPFRGFF   98 
AAO62041/A      89 ------SS-----------------LCPVSGWA   98 
AAO62044/A      88 ------KD-----------------LCPVKGWA   97 
AAO62064/A      89 ------KP--------------L---CEVNSWH   98 
AAT08005/A      87 ------EP-----------------LCDAKGFA   96 
AAT37407/A      87 ----PPLP--------------L---CPFRGFF   98 
AAT37414/A      83 THFRLPLP--------------L---CPFKGFF   98 
AAT65406/A      89 ------KP--------------L---CEVNSWH   98 
AAX78821/A      89 ------KP-----------------QCQITGFA   98 
AAY52584/A      89 ------KP-----------------QCQITGFA   98 
AAY87411/A      89 ------KP-----------------QCQITGFA   98 
ABB17695/A      86 ------EP-----------------LCNVSGFA   95 
ABB19439/A      89 ------KP--------------L---CEVNSWH   98 
ABB19610/A      89 ------SS-----------------LCPISGWA   98 
ABB19747/A      86 ------EP-----------------LCNVSGFA   95 
ABB19914/A      88 ------RD-----------------LCPIKGWA   97 
ABB20088/A      88 ------RD-----------------LCPIRGWA   97 
ABB20104/A      87 ------KS--------------L---CKVEGWV   96 
ABB20243/A      90 ------KG--------------L---CTINSWH   99 
ABB20375/A      89 ------KP--------------L---CEVNSWH   98 
ABB20475/A      89 ------KP-----------------QCQITGFA   98 
ABB20501/A      84 ----LNKS--------------L---CNVEGWV   95 
ABB21754/A      90 ------KG--------------L---CTINSWH   99 
ABB87198/A      86 ------EP-----------------LCNVSGFA   95 
ABB87732/A      88 ------RN-----------------LCPIKGWA   97 
ABB87959/A      87 ------KS--------------L---CKVEGWV   96 
ABB88058/A      83 RIFKPSLP--------------L---CPFRGFF   98 
ABB88351/A      89 ------KP--------------L---CEANSWH   98 
ABC02269/A      89 ------KP-----------------QCKITGFA   98 
ABF47958/A      73 ------SS-----------------LCPIRGWA   82 
ABG37189/A      89 ------KP-----------------QCKITGFA   98 
ABH04381/A      83 RLF---KP--------------PLPLCPFRGFF   98 
ABI30359/A      89 ------KP-----------------QCQITGFA   98 
ABI84399/A      85 --FRLPLP--------------L---CPFRGFF   98 
ABI84436/A      86 ------EP-----------------LCNVSGFA   95 
ABI84475/A      88 ------RD-----------------LCPVKGWA   97 
ABI84973/A      87 ----PSLP--------------L---CPFRGFF   98 
ABI84982/A      72 ----INKGTNLKAPNYLLLNKSL---CSVEGWV   97 
ABI92217/A      89 ------KP--------------L---CEVNSWH   98 
ABJ09099/A      87 ------EP-----------------LCDAKGFA   96 
ABK32099/A      88 ------KD-----------------LCPVKGWA   97 
ABP49198/A      90 ------KG--------------L---CTINSWH   99 
ABP49253/A      90 ------KG--------------L---CTINSWH   99 
ABQ97208/A      89 ------KP-----------------QCKITGFA   98 
ABR28683/A      89 ------SS-----------------LCPISGWA   98 
ABR37723/A      90 ------KG--------------L---CTINSWH   99 
ABS50124/A      89 ------SS-----------------LCPISGWA   98 
ABS89357/A      86 ----LNKS--------------L---CNVEGWV   97 
ABU95382/A      89 ------SS-----------------LCPISGWA   98 
ABV25648/A      89 ------SS-----------------LCPISGWA   98 
ABV29593/A      89 ------SS-----------------LCPISGWA   98 
ABV82576/A      89 ------SS-----------------LCPISGWA   98 
ABV82587/A      89 ------SP-----------------LCPISGWA   98 
ABW95951/A      69 ------SS-----------------LCPISGWA   78 
ABX88802/A      89 ------KP-----------------QCQITGFA   98 
ABY84687/A      89 ------SS-----------------LCPISGWA   98 
ACA04655/A      89 ------KP--------------L---CEVSSWH   98 
ACA04738/A      86 ----LNKS--------------L---CSVEGWV   97 
ACA04739/A      87 ------EA-----------------ICDVKGFA   96 
ACA04740/A      90 ------RG--------------L---CTINSWH   99 
ACA14299/A      89 ------KP-----------------QCQITGFA   98 
ACB70583/A      69 ------SS-----------------LCPINGWA   78 
ACD47117/A      89 ------KP-----------------QCQITGFA   98 
ACD65204/A      89 ------SS-----------------LCPISGWA   98 
ACD76864/A      89 ------KP-----------------QCQITGFA   98 
ACD85421/A      87 ------EP-----------------LCEAQGFA   96 
ACE73320/A      88 ------RD-----------------LCPIRGWA   97 
ACE76594/A      89 ------KP-----------------QCQITGFA   98 
ACE78976/A      88 ------KP--------------L---CEVNSWH   97 
ACE78981/A      88 ------KP--------------L---CEVNSWH   97 
ACF20219/A      85 ----LNKS--------------L---CKVEGWV   96 
ACF25054/A      89 ------KP-----------------QCQITGFA   98 
ACF25354/A      85 ----LNKS--------------L---CKVEGWV   96 
ACF33655/A      83 RPFKSPLP--------------L---CPFRGFF   98 
ACF36500/A      89 ------KP-----------------QCNITGFA   98 
ACF93490/A      87 ------EP-----------------LCEAQGFA   96 
ACI41114/A      83 RPFKSPLP--------------L---CPFRGFF   98 
ACI48786/A      87 ------EP-----------------LCEAQGFA   96 
ACN65661/A      86 ----LNKS--------------L---CNVEGWV   97 
ACO24985/A      88 ------SS-----------------LCPISGWA   97 
ACP50710/A      89 ------KP-----------------QCQITGFV   98 
ACQ73415/A      84 ----LNKS--------------L---CNVEGWV   95 
ACR66907/A      87 ----SSLP--------------L---CPFRGFF   98 
ACR83968/A      89 ------TS-----------------LCPVSGWA   98 
ACS68311/A      90 ------KG--------------L---CTINSWH   99 
ACS92897/A      89 ------KP-----------------QCNITGFA   98 
ACT67761/A      89 ------KP-----------------QCDITGFA   98 
ACT97062/A      87 ------EA-----------------ICDVRGFA   96 
ACV41710/A      87 ------EA-----------------LCDAKGFA   96 
ADA71169/A      89 ------SS-----------------LCSISGWA   98 
ADK70163/A      88 ------KD-----------------LCPVKGWA   97 
ADU17627/A      90 ------KG--------------L---CTINSWH   99 
ADU20276/A      86 ------EP-----------------LCNVSGFA   95 
ADU20397/A      83 RPFKSPLP--------------L---CPFRGFF   98 
ADU53220/A      88 ------RD-----------------LCPIRGWA   97 
AEB89856/A      90 ------KG--------------L---CIINSWH   99 
AEB89867/A      87 ------EA-----------------ICDVKGFA   96 
AEK50939/A      89 ------KP--------------L---CEVNSWH   98 
AEK50961/A      85 ----LNKS--------------L---CKVEGWV   96 
BAA06132/A      89 ------KP-----------------QCKITGFA   98 
BAA06718/A      89 ------SS-----------------LCPISGWA   98 
BAF03532/A      85 ------EP-----------------LCEVSGFA   94 
BAF03569/A      85 ------EP-----------------LCEVSGFA   94 
BAF34378/A      89 ------KP-----------------QCQITGFA   98 
BAF34925/A      89 ------KP-----------------QCQITGFA   98 
BAF43417/A      86 ------EP-----------------LCNVSGFA   95 
BAF43457/A      87 ------EA-----------------ICDVKGFA   96 
BAF43461/A      85 ------EP-----------------LCEVSGFA   94 
BAF46763/A      87 ----PSLP--------------L---CPFRGFF   98 
BAF46905/A      89 ------KP--------------L---CEVNSWH   98 
BAF48640/A      89 ------KP-----------------QCQITGFA   98 
BAF48644/A      87 ----PSLP--------------L---CSFRGFF   98 
BAF48646/A      86 ----LNKS--------------L---CNVEGWV   97 
BAF63047/A      89 ------SS-----------------LCSISGWA   98 
BAG66251/A      87 ------EP-----------------LCDAKGFA   96 
BAG66259/A      83 RPFKPPLP--------------L---CPFRGFF   98 
BAG66279/A      87 ------EA-----------------ICDVKGFA   96 
BAH02172/A      89 ------KP-----------------QCKITGFA   98 
BAH70320/A      86 ----LNKS--------------L---CNVEGWV   97 
BAI39637/A      69 ------SS-----------------LCPIRGWA   78 
BAI48894/A      89 ------SS-----------------LCPISGWA   98 
BAI50030/A      87 ----SSLP--------------L---CPFRGFF   98 
BAJ07979/A      85 ------EP-----------------LCEISGFA   94 
BAJ10562/A      89 ------KP--------------L---CEVNSWH   98 
CAA36475/A      67 ------SS-----------------LCPISGWA   76 
CAC36997/A      89 ------KP-----------------QCKITGFA   98 
CAD57252/A      89 ------SS-----------------LCSISGWA   98 
CAO82694/A     100 ------SS-----------------LCPVSGWA  109 
CAP69845/A      88 ------KD-----------------LCPVKGWA   97 

cons          595                           *    :   627 


AAA43735/B      94 KALLISPHRFGETRGNSAPLIIREPFVACGPKE  126 
AAA43743/B      94 KALLISPHRFGEARGNSAPLIIREPFIACGPKE  126 
AAA43749/B      94 KALLISPHRFGEIKGNSAPLIIREPFVACGPKE  126

AAU94779/B      94 KALLISPHRFGETKGNSAPLIIREPFIACGPKE  126

ABL84344/B      94 KALLISPHRFGETKGNSAPLIIREPFIACGPKE  126

ABO72374/B      94 KALLISPHRFGETKGNSAPLIIREPFIACGPKE  126

ABQ81846/B      94 KALLISPHRFGETRGNSAPLIIREPFVACGPKE  126

ACU12759/B      94 KALLISPHRFGETKGNSAPLIIREPFIACGPKE  126

BAB32609/B      94 KALLISPHRFGEAKGNSAPLIIREPFIACGPKE  126     
AAA43093/A      96 VIAKDNAIRFGE---SEQIIVTREPYVSCDPLS  125 
AAA43429/A      97 PFSKDNGIRIGS---KGHVFVIREPFVSCSPLE  126 
AAA43449/A      99 IYSKDNSIRIGS---KGDVFVIREPFISCSHLE  128 
AAA43575/A     100 IYGKDNAVRIGE---DSDVLVTREPYVSCDPDE  129 
AAD49005/A      99 PFSKDNSIRLSA---GGDIWVTREPYVSCGPSK  128 
AAD49007/A      99 PFSKDNSIRLSA---GGDIWVTREPYVSCGLGK  128 
AAF77036/A      99 IYSKDNGIRIGS---KGDVFVIREPFISCSHLE  128 
AAO46232/A      99 PFSKDNSIRLSA---GGDIWVTREPYVSCDPGK  128 
AAO46822/A      99 VHSKDNGIRIGS---KGDVFVIREPFISCSHLE  128 
AAO62036/A      99 PFHKDNAIRLGE---NKDVIVTREPYVSCDNNN  128 
AAO62041/A      99 IYSKDNGIRIGS---KGDVFVIREPFISCSHLE  128 
AAO62044/A      98 PLSKDNGIRIGS---RGEVFVIREPFISCSINE  127 
AAO62064/A      99 ILSKDNAIRIGE---DAHILVTREPYLSCGPQG  128 
AAT08005/A      97 PFSKDNGIRIGS---RGHVFVIREPFVSCSPTE  126 
AAT37407/A      99 PFHKDNAIRLGE---NKDVIVTREPYVSCDNDD  128 
AAT37414/A      99 PFHKDNALRLAE---NKDVLVTREPYISCDNVG  128 
AAT65406/A      99 ILSKDNAIRIGE---DAHILVTREPYLSCGPHE  128  
AAX78821/A      99 PFSKDNSIRLSA---GGDIWVTREPYVSCDPGK  128 
AAY52584/A      99 PFSKDNSIRLSA---GGDIWVTREPYVSCGLGK  128 
AAY87411/A      99 PFSKDNSIRLSA---GGDIWVTREPYVSCDPGK  128 
ABB17695/A      96 IVSKDNGIRIGS---RGHVFVIREPFVACGPTE  125 
ABB19439/A      99 ILSKDNAIRIGE---DAHILVTREPYLSCGPNE  128 
ABB19610/A      99 IYSKDNGIRIGS---KGDVFVIREPFISCSHLE  128 
ABB19747/A      96 IVSKDNGIRIGS---RGHVFVIREPFVACGPAE  125 
ABB19914/A      98 PLSKDNGIRIGS---RGEVFVIREPFISCSISE  127 
ABB20088/A      98 PLSKDNGIRIGS---RGEVFVIREPFISCSISE  127 
ABB20104/A      97 VVAKDNAIRFGE---SEQIIVTREPYVSCDPSG  126 
ABB20243/A     100 IFGKDNAIRIGE---NSDVLVTREPYVSCDPDE  129 
ABB20375/A      99 ILSKDNAIRIGE---DAHILVTREPYLSCDPQG  128 
ABB20475/A      99 PFSKDNSIRLSA---GGDIWVTREPYVSCDPGK  128 
ABB20501/A      96 VIAKDNAIRFGE---SEQIIVTREPYVSCDPLS  125 
ABB21754/A     100 IFGKDNAIRIGE---NSDVLVTREPYVSCDPDE  129 
ABB87198/A      96 IVSKDNGIRIGS---RGHVFVIREPFVACGPTE  125 
ABB87732/A      98 PLSKDNGIRIGS---RGEVFVIREPFISCSISE  127 
ABB87959/A      97 VVAKDNAIRFGE---SEQVIVTREPYVSCDPLG  126 
ABB88058/A      99 PFHKDNAIRLGE---NKDVIVTREPYVSCDNDN  128 
ABB88351/A      99 ILSKDNAIRIGE---DAHILVTREPYLSCDPQG  128 
ABC02269/A      99 PFSKDNSIRLSA---GGDIWVTREPYVSCDPGK  128 
ABF47958/A      83 IHSKDNGIRIGS---KGDVFVIREPFISCSHLE  112 
ABG37189/A      99 PFSKDNSIRLSA---GGDIWVTREPYVSCDPGK  128 
ABH04381/A      99 PFHKDNAIRLGE---NKDVIVTREPYVSCDNDN  128 
ABI30359/A      99 PFSKDNSIRLSA---GGDIWVTREPYVSCDTSK  128 
ABI84399/A      99 PFHKDNALRLAE---NKDVLVTREPYISCDNRG  128 
ABI84436/A      96 IVSKDNGIRIGS---RGHVFVIREPFVACGPTE  125 
ABI84475/A      98 PLSKDNGIRIGS---RGEVFVIREPFISCSINE  127 
ABI84973/A      99 PFHKDNAIRLGE---NKDVIVTREPYVSCDNDN  128 
ABI84982/A      98 VIAKDNAIRFGE---SEQIIVTREPYVSCDPSG  127 
ABI92217/A      99 ILSKDNAIRIGE---DAHILVTREPYLSCGPHE  128 
ABJ09099/A      97 PFSKDNGIRIGS---RGHVFVIREPFVSCSPTE  126 
ABK32099/A      98 PLSKDNGIRIGS---RGEVSVIREPFISCSIHE  127 
ABP49198/A     100 IYGKDNAVRIGE---DSDVLVTREPYVSCDPDE  129 
ABP49253/A     100 IYGKDNAVRIGE---DSDVLVTREPYVSCEPDE  129 
ABQ97208/A      99 PFSKDNSIRLSA---GGDIWVTREPYVSCDPGK  128 
ABR28683/A      99 IYSKDNSIRIGS---KGDIFVMREPFISCSHLE  128 
ABR37723/A     100 IFGKDNAIRIGE---NSDVLVTREPYVSCDPDE  129 
ABS50124/A      99 IYSKDNSIRIGS---KGDIFVIREPFISCSHLE  128 
ABS89357/A      98 VVAKDNAIRFGE---SEQIIVTREPYVSCDPSG  127 
ABU95382/A      99 IYSKDNSIRIGS---KGDVFVIREPFISCSHLE  128 
ABV25648/A      99 IYSKDNSIRIGS---KGDIFVIREPFISCSHLE  128 
ABV29593/A      99 IYSKDNGVRIGS---KGDVFVIREPFISCSHLE  128 
ABV82576/A      99 IYSKDNSIRIGS---KGDIFVIREPFISCSHLE  128 
ABV82587/A      99 IYSKDNSIRIGS---KGDIFVMREPFISCSHLE  128 
ABW95951/A      79 VHSKDNGIRIGS---KGDVFVIREPFISCSHLE  108 
ABX88802/A      99 PFSKDNSIRLSA---GGDIWVTREPYVSCSPGK  128 
ABY84687/A      99 IYSKDNSIRIGS---KGDIFVIREPFISCSHLE  128 
ACA04655/A      99 ILSKDNAIRIGE---DAHILVTREPYLSCDPQG  128 
ACA04738/A      98 VIAKDNAIRFGE---SEQIIVTREPYVSCDPSG  127 
ACA04739/A      97 PFSKDNGIRIGS---RGHIFVIREPFVSCSPIE  126 
ACA04740/A     100 IFGKDNAVRIGE---DSDVLVTRESYVSCDPDE  129 
ACA14299/A      99 PFSKDNSIRLSA---GGDIWVTREPYVSCDPSK  128 
ACB70583/A      79 VYSKDNSIRIGS---KGDVFVIREPFISCSHLE  108 
ACD47117/A      99 PFSKDNSIRLSA---GGDIWVTREPYVSCGLGK  128 
ACD65204/A      99 IYSKDNSIRIGS---KGDIFVIREPFISCSHLE  128 
ACD76864/A      99 PFSKDNSIRLSA---GGDIWVTREPYVSCDTSK  128 
ACD85421/A      97 PFSKDNGIRIGS---RGHVFVIREPFVSCSPLE  126 
ACE73320/A      98 PLSKDNGIRIGS---RGEVFVIREPFISCSINE  127 
ACE76594/A      99 PFSKDNSIRLSA---GGDIWVTREPYVSCSPGK  128 
ACE78976/A      98 ILTKDNAIRIGE---EAHILVTREPYLSCDPQG  127 
ACE78981/A      98 ILSKDNAIRIGE---DAHVLVTREPYLSCDPQG  127 
ACF20219/A      97 VVAKDNAIRXGE---SEQIIVIREPYVSCDPLG  126 
ACF25054/A      99 PFSKDNSIRLSA---GGDIWVTREPYVSCDPSK  128 
ACF25354/A      97 VVAKDNAIRFGE---SEQIIVTREPYVSCDPLG  126 
ACF33655/A      99 PFHKDNAIRLGE---NKDVIVTREPYVSCDNDN  128 
ACF36500/A      99 PFSKDNSIRLSA---GGDIWVTREPYVSCDPDK  128 
ACF93490/A      97 PFSKDNGIRIGS---RGHVFVIREPFVSCSPSE  126 
ACI41114/A      99 PFHKDNAIRLGE---NKDVIVTREPYVSCDNDN  128 
ACI48786/A      97 PFSKDNGIRIGS---RGHVFVIREPFVSCSPSE  126 
ACN65661/A      98 VIEKDNAVRFGE---SEQIIVTREPYVSCDPTG  127 
ACO24985/A      98 IYSKDNGVRIGS---KGDVFVIREPFISCSHLE  127 
ACP50710/A      99 PFSKDNSIRLSA---GGDIWVTREPYVSCGLSK  128 
ACQ73415/A      96 VIAKDNAIRFGE---SEQIIVTREPYVSCDPLS  125 
ACR66907/A      99 PFHKDNAIRLGE---NKDVIVTREPYVSCDNDD  128 
ACR83968/A      99 IYSKDNSVRIGS---KGDVFVIREPFISCSHLE  128 
ACS68311/A     100 IYGKDNAVRIGE---DSDVLVTREPYVSCDPDE  129 
ACS92897/A      99 PFSKDNSIRLSA---GGDIWVTREPYVSCDPDK  128 
ACT67761/A      99 PFSKDNSIRLSA---GGDIWVTREPYVSCDPDK  128 
ACT97062/A      97 PFSKDNGIRIGS---RGHVFVIREPFVSCSPIE  126  
ACV41710/A      97 PFSKDNGIRIGS---RGHVFVIREPFVSCSPIE  126 
ADA71169/A      99 IYTKDNSIRIGS---KGDVFVIREPFISCSHLE  128 
ADK70163/A      98 PLSKDNGIRIGS---RGEVFVIREPFISCSINE  127 
ADU17627/A     100 IYGKDNAVRIGE---NSDVLVTREPYVSCDPDE  129 
ADU20276/A      96 IVSKDNGIRIGS---RGHVFVIREPFVACGPTE  125 
ADU20397/A      99 PFHKDNAIRLGE---NKDVIVTREPYVSCDNDN  128 
ADU53220/A      98 PLSKDNGIRIGS---RGEVFVIREPFISCSISE  127 
AEB89856/A     100 IYGKDNAVRIGE---SSDVLVTREPYVSCDPDE  129 
AEB89867/A      97 PFSKDNGIRIGS---RGHVFVIREPFVSCSPIE  126 
AEK50939/A      99 ILSKDNAIRIGE---DAHILVTREPYLSCGPHE  128 
AEK50961/A      97 VVAKDNAIRFGE---GEQIIVTREPYVSCDPLG  126 
BAA06132/A      99 PFSKDNSIRLSA---GGDIWVTREPYVSCDPGK  128 
BAA06718/A      99 IYSKDNSIRIGS---KGDVFVIREPFISCSHLE  128 
BAF03532/A      95 IVSKDNGIRIGS---RGHVFVIREPFVSCGPSE  124 
BAF03569/A      95 IVSKDNGIRIGS---RGHVFVIREPFVACGPSE  124 
BAF34378/A      99 PFSKDNSIRLSA---GGDIWVTREPYVSCGPSK  128 
BAF34925/A      99 PFSKDNSIRLSA---GGDIWVTREPYVSCGPGK  128 
BAF43417/A      96 IVSKDNGIRIGS---RGHVFVIREPFVACGPTE  125 
BAF43457/A      97 PFSKDNGIRIGS---RGHVFVIREPFISCSPAE  126 
BAF43461/A      95 IVSKDNGIRIGS---RGHVFVIREPFVACGPSE  124 
BAF46763/A      99 PFHKDNAIRLGE---NKDVIVTREPYVSCDNDN  128 
BAF46905/A      99 ILSKDNAIRIGE---DAHILVTREPYLSCDPQG  128 
BAF48640/A      99 PFSKDNSIRLSA---GGNIWVTREPYVSCDPGK  128 
BAF48644/A      99 PFHKDNAIRLGE---NKDVIVTREPYVSCDNYN  128 
BAF48646/A      98 VIAKDNAVRFGE---SEQIIVTREPYVSCDPTG  127 
BAF63047/A      99 IYTKDNSIRIGS---KGDVFVIREPFISCSHLE  128 
BAG66251/A      97 PFSKDNGIRIGS---RGHVFVIREPFVSCSPTE  126 
BAG66259/A      99 PFHKDNAIRLGE---NKDVIVTREPYVSCDNDN  128 
BAG66279/A      97 PFSKDNGIRIGS---RGHVFAIREPFVSCSPTE  126 
BAH02172/A      99 PFSKDNSIRLSA---GGDIWVTREPYVSCDPGK  128 
BAH70320/A      98 VIAKDNAVRFGE---SEQIIVTREPYVSCDPTG  127 
BAI39637/A      79 IHSKDNSIRIGS---KGDVFVIREPFISCSHLE  108 
BAI48894/A      99 IYSKDNGIRIGS---KGDVFVIREPFISCSHLE  128 
BAI50030/A      99 PFHKDNAIRLGE---NKDVIVTREPYVSCDNDN  128 
BAJ07979/A      95 IVSKDNGIRIGS---RGHVFVIREPFVACGPSE  124 
BAJ10562/A      99 ILSKDNAIRIGE---DAHILVTREPYLSCDPQG  128 
CAA36475/A      77 IYSKDNGIRIGS---KGDVFVIREPFISCSHLE  106 
CAC36997/A      99 PFSKDNSIRLSA---GGDIWVTREPYVSCDPGK  128 
CAD57252/A      99 IYTKDNSIRIGS---KGDVFVIREPFISCSHLE  128 
CAO82694/A     110 IYSKDNSIRIGS---KGDVFVIREPFISCSHLE  139 
CAP69845/A      98 PLSKDNGIRIGS---RGEVFVIREPFISCSINE  127 

cons           628      .  * .           **.:::*      660 

AAA43735/B     127 CRHFALTHYAAQPGGYYNGTRKDRNKLRHLISV  159 
AAA43743/B     127 CKHFALTHYAAQPGGYYNGTRKDRNKLRHLISV  159 
AAA43749/B     127 CRHFALTHYAAQPGGYYNGTRKDRNKLRHLVSV  159

AAU94779/B     127 CKHFALTHYAAQPGGYYNGTREDRNKLRHLISV  159

ABL84344/B     127 CKHFALTHYAAQPGGYYNGTREDRNKLRHLISV  159 
ABO72374/B     127 CKHFALTHYAAQPGGYYNGTREDRNKLRHLISV  159

ABQ81846/B     127 CRHFALTHYAAQPGGYYNGTRKDRNKLRHLISV  159

ACU12759/B     127 CKHFALTHYAAQPGGYYNGTRGDRNKLRHLISV  159

BAB32609/B     127 CKHFALTHYAAQPGGYYNGTREDRNKLRHLISV  159

AAA43093/A     126 CKMYALHQGTTIRNKHSNSTTHDRTAFRGLIST  158 
AAA43429/A     127 CRTFFLTQGSLLNDKHSNGTVKDRSPYRTLMSV  159 
AAA43449/A     129 CRTFFLTQGALLNDKHSNGTVKDRSPYRALMSC  161 
AAA43575/A     130 CRFYALSQGTTIRGKHSNGTIHDRSQYRDLISW  162 
AAD49005/A     129 CYQFALGQGATLDNKHSNGTIHDRTLNRTLLRN  161 
AAD49007/A     129 CYQFALGQGTTLNNKHSNGTTHDRIPHRTLLMN  161 
AAF77036/A     129 CRTFFLTQGALLNDKHSNGTVKDRSPYRTLMSC  161 
AAO46232/A     129 CYQFALGQGTTLDNKHSNDTIHDRIPHRTLLMN  161 
AAO46822/A     129 CRTFFLTQGALLNDKHSNGTVKDRSPHRTLMSC  161 
AAO62036/A     129 CWSFALAQGALLGTQHSNGTIKDRTPYRSLIRF  161 
AAO62041/A     129 CRTFFLTQGALLNDKHSNGTVKDRSPYRTLMSC  161 
AAO62044/A     128 CRTFFLTQGALLNDKHSNGTVKDRSPFRTLMSC  160 
AAO62064/A     129 CRMFALSQGTTLRGRHANGTIHDRSPYRALISW  161 
AAT08005/A     127 CRTFFLTQGSLLNDKHSNGTVKDRSPYRTLMSV  159 
AAT37407/A     129 CWSFALAQGALLGTKHSNGTIKDRTPYRSLIRF  161 
AAT37414/A     129 CWSFALAQGALLGTKHSNGTNKDRTPYRSLIKF  161 
AAT65406/A     129 CRMFALSQGTTLRGRHANGTIHDRSPFRALISW  161 
AAX78821/A     129 CYQFALGQGTTLDNKHSNGTIHDRIPHRTLLMN  161 
AAY52584/A     129 CYQFALGQGTTLENKHSNGTALDRTPHRTLLMN  161 
AAY87411/A     129 CYQFALGQGTTLDNKHSNGTIHDRIPHRTLLMN  161 
ABB17695/A     126 CRTFFLTQGALLNDKHSNNTVKDRSPYRALMSV  158 
ABB19439/A     129 CRMFALSQGTTLRGRHANGTIHDRSPFRALISW  161 
ABB19610/A     129 CRTFFLTQGALLNDKHSNGTVKDRSPYRTLMSC  161 
ABB19747/A     126 CRTFFLTQGALLNDKHSNNTVKDRSPYRALMSV  158 
ABB19914/A     128 CRTFFLTQGALLNDKHSNGTVKDRSPFRTLMSC  160 
ABB20088/A     128 CRTFFLTQGALLNDKHSNGTVKDRSPFRTLMSC  160 
ABB20104/A     127 CRMYALHQGTTIRNKHSNGTIHDRTAFRGLIST  159 
ABB20243/A     130 CRFYALSQGTTIRGKHSNGTIHDRSQYRALVSW  162 
ABB20375/A     129 CRMFALSQGTTLRGRHANGTIHDRSPYRALISW  161 
ABB20475/A     129 CYQFALGQGTTLDNKHSNGTIHDRIPHRTLLMN  161 
ABB20501/A     126 CKMYALHQGTTIRNKHSNGTTHDRTAFRGLIST  158 
ABB21754/A     130 CRFYALSQGTTIRGKHSNGTIHDRSQYRALVSW  162 
ABB87198/A     126 CRTFFLTQGALLNDKHSNNTVKDRSPYRALMSV  158 
ABB87732/A     128 CRTFFLTQGALLNDKHSNGTVKDRSPFRTLMSC  160 
ABB87959/A     127 CRMYALHQGTTIRNKHSNGTIHDRTAFRGLIST  159 
ABB88058/A     129 CWSFALAQGALLGTKHSNGTIKDRTPYRSLIRF  161 
ABB88351/A     129 CRMFALSQGTTLRGRHANGTIHDRSPFRALISW  161 
ABC02269/A     129 CYQFALGQGTTLDNKHSNDTIHDRTPHRTLLMN  161 
ABF47958/A     113 CRTFFLTQGALLNDKHSRGTFKDRSPYRALMSC  145 
ABG37189/A     129 CYQFALGQGTTLNNRHSNDTVHDRTPYRTLLMN  161 
ABH04381/A     129 CWSFALAQGALLGTKHSNGTIKDRTPYRSLIRF  161 
ABI30359/A     129 CYQFALGQGTTLDNKHSNGTIHDRIPHRTLLMN  161 
ABI84399/A     129 CWSFALAQGALLGTKHSNGTNKDRTPYRSLIRF  161 
ABI84436/A     126 CRTFFLTQGALLNDKHSNNTVKDRSPYRALMSV  158 
ABI84475/A     128 CRTFFLTQGALLNDKHSNGTVKDRSPFRTLMSC  160 
ABI84973/A     129 CWSFALAQGALLGTKHSNGTIKDRTPYRSLIRF  161 
ABI84982/A     128 CKMYALHQGTTIRNKHSNGTIHDRTTFRGLLST  160 
ABI92217/A     129 CRMFALSQGTTLRGRHANGTIHDRSPFRALISW  161 
ABJ09099/A     127 CRTFFLTQGSLLNDKHSNGTVKDRSPYRTLMSV  159 
ABK32099/A     128 CRTFFLTQGALLNDKHSNGTVKDRSPFRTLMSC  160 
ABP49198/A     130 CRFYALSQGTTIRGKHSNGTIHDRSQYRALISW  162 
ABP49253/A     130 CRFYALSQGTTIRGKHSNGTIHDRSQYRALISW  162  
ABQ97208/A     129 CYQFALGQGTTLNNKHSNDTIHDRTPYRTLLMN  161 
ABR28683/A     129 CRTFFLTQGALLNDRHSNGTVKDRSPYRTLMSC  161 
ABR37723/A     130 CRFYALSQGTTIRGKHSNGTIHDRSQYRALVSW  162 
ABS50124/A     129 CRTFFLTQGALLNDRHSNGTVKDRSPYRTLMSC  161 
ABS89357/A     128 CKMYALHQGTTIRNKHSNGTIHDRTAFRGLIST  160 
ABU95382/A     129 CRTFFLTQGALLNDKHSNGTVKDRSPYRTLMSC  161 
ABV25648/A     129 CRTFFLTQGALLNDRHSNGTVKDRSPYRTLMSC  161 
ABV29593/A     129 CRTFFLTQGALLNDKHSNGTVKDRSPYRTLMSC  161 
ABV82576/A     129 CRTFFLTQGALLNDRHSNGTVKDRSPYRTLMSC  161 
ABV82587/A     129 CRTFFLTQGALLNDKHSNGTVKDRSPYRTLMSC  161 
ABW95951/A     109 CRTFFLTQGALLNDKHSNGTVKDRSPHRTLMSC  141 
ABX88802/A     129 CYQFALGQGTTLNNKHSNGTIHDRIPHRTLLMN  161 
ABY84687/A     129 CRTFFLTQGALLNDRHSNGTVKDRSPYRTLMSC  161 
ACA04655/A     129 CRMFALSQGTTLRGRHVNGTIHDRSPFRALISW  161 
ACA04738/A     128 CKMYALHQGTTIRNKHSNGTIHDRTAFRGLIST  160 
ACA04739/A     127 CRTFFLTQGSLLNDKHSNGTVKDRSPFRTLMSV  159 
ACA04740/A     130 CRFYALSQGTTIRGKHSNGTIHDRSQYRALISW  162 
ACA14299/A     129 CYQFALGQGTTLDNKHSNGTIHDRIPHRTLLMN  161 
ACB70583/A     109 CRTFFLTQGALLNDKHSNGTVKDRSPHRTLMSC  141 
ACD47117/A     129 CYQFALGQGTTLNNKHSNGTTHDRSPHRTLLMN  161 
ACD65204/A     129 CRTFFLTQGALLNDRHSNGTVKDRSPYRTLMSC  161 
ACD76864/A     129 CYQFALGQGTTLDNKHSNGTIHDRIPHRTLLMN  161 
ACD85421/A     127 CRTFFLTQGSLLNDKHSNGTVKDRSPYRTLMSV  159 
ACE73320/A     128 CRTFFLTQGALLNDKHSNGTVKDRSPFRTLMSC  160 
ACE76594/A     129 CYQFALGQGTTLNNKHSNGTIHDRIPHRTLLMN  161 
ACE78976/A     128 CRMFALSQGTTLRGRHANGTIHDRSPFRALISW  160 
ACE78981/A     128 CRMFALSQGTTLRGRHANGTIHDRSPFRALISW  160 
ACF20219/A     127 CKMYALHQGTTIRNRHSNGTIXDRTAFRGLIST  159 
ACF25054/A     129 CYQFALGQGTTLDNKHSNGTIHDRIPHRTLLMN  161 
ACF25354/A     127 CKMYALHQGTTIRNKHSNGTIHDRTAFRGLIST  159 
ACF33655/A     129 CWSFALAQGALLGTKHSNGTIKDRTPYRSLIRF  161 
ACF36500/A     129 CYQFALGQGTTLNNVHSNDTVHDRTPYRTLLMN  161 
ACF93490/A     127 CRTFFLTQGSLLNDKHSNGTVKDRSPYRTLMSV  159 
ACI41114/A     129 CWSFALAQGALLGTKHSNGTIKDRTPYRSLIRF  161 
ACI48786/A     127 CRTFFLTQGSLLNDKHSNGTVKDRSPYRTLMSV  159 
ACN65661/A     128 CKMYALHQGTTIRNKHSNGTIHDRTAFRGLIST  160 
ACO24985/A     128 CRTFFLTQGALLNDKHSNGTVKDRSPYRTLMSC  160 
ACP50710/A     129 CYQFALGQGTTLNNKHSNGTTHDRSPYRTLLMS  161 
ACQ73415/A     126 CKMYALHQGTTIRNKHSNSTTHDRTAFRGLIST  158 
ACR66907/A     129 CWSFALAQGALLGTKHSNGTIKDRTPYRSLIRF  161 
ACR83968/A     129 CRTFFLTQGALLNDKHSNGTIKDRSPYRTLMSC  161 
ACS68311/A     130 CRFYALSQGTTIRGKHSNGTIHDRSQYRALISW  162 
ACS92897/A     129 CYQFALGQGTTLNNGHSNDTVHDRTPYRTLLMN  161 
ACT67761/A     129 CYQFALGQGTTLNNVHSNNTVRBRTPYRTLLMN  161 
ACT97062/A     127 CRTFFLTQGSLLNDKHSNGTVKDRSPFRTLMSV  159 
ACV41710/A     127 CRTFFLTQGSLLNDKHSNGTVKDRSPYRTLMSV  159 
ADA71169/A     129 CKTFFLTQGALLNDKHSNGTVKDRSPYRALMSC  161 
ADK70163/A     128 CRTFFLTQGALLNDKHSNGTVKDRSPFRTLMSC  160 
ADU17627/A     130 CRFYALSQGTTIRGKHSNGTIHDRSQYRALISW  162 
ADU20276/A     126 CRTFFLTQGALLNDKHSNNTVKDRSPYRALMSV  158 
ADU20397/A     129 CWSFALAQGALLGTKHSNGTIKDRTPYRSLIRF  161 
ADU53220/A     128 CRTFFLTQGALLNDKHSNGTVKDRSPFRTLMSC  160 
AEB89856/A     130 CRFYALSQGTTIRGKHSNGTIHDRSQYRALISW  162 
AEB89867/A     127 CRTFFLTQGSLLNDKHSNGTVKDRSPFRTLMSV  159 
AEK50939/A     129 CRMFALSQGTTLKGRHANGTIHDRSPFRALISW  161 
AEK50961/A     127 CKMYALHQGTTIRNKHSNGTIHDRTAFRGLIST  159 
BAA06132/A     129 CYQFALGQGTTLNNKHSNDTIHDRTPHRTLLMN  161 
BAA06718/A     129 CRTFFLTQGALLNDKHSNGTVKDRSPYRRLMSC  161 
BAF03532/A     125 CRTFFLTQGALLNDKHSNNTVKDRSPYRALMSV  157 
BAF03569/A     125 CRTFFLTQGALLNDKHSNNTVKDRSPYRALMSV  157 
BAF34378/A     129 CYQFALGQGTTLDNKHSNGTIHDRTPHRTLLMN  161 
BAF34925/A     129 CYQFALGQGTTLDNKHSNGTIHDRIPHRTLLMN  161 
BAF43417/A     126 CRTFFLTQGALLNDKHSNNTVKDRSPYRALMSV  158 
BAF43457/A     127 CRTFFLTQGSLLNDKHSNGTVKDRSPFRTLMSV  159 
BAF43461/A     125 CRTFFLTQGALLNDKHSNNTVKDRSPYRALMSV  157 
BAF46763/A     129 CWSFALAQGALLGTKHSNGTIKDRTPYRSLIRF  161 
BAF46905/A     129 CRMFALSQGTTLRGRHANGTIHDRSPFRALISW  161 
BAF48640/A     129 CYQFALGQGTTLDNKHSNGTIHDRIPHRTLLMN  161 
BAF48644/A     129 CWSFALAQGALLGTKHSNGTIKDRTPYRSLIRF  161 
BAF48646/A     128 CKMYALHQGTTIRNKHSNGTIHDRTAFRGLMST  160 
BAF63047/A     129 CRTFFLTQGALLNDKHSNGTVKDRSPYRALMSC  161 
BAG66251/A     127 CRTFFLTQGSLLNDKHSNGTVKDRSPYRTLMSV  159 
BAG66259/A     129 CWSFALAQGALLGTKHSNGTIKDRTPYRSLIRF  161 
BAG66279/A     127 CRTFFLTQGSLLNDKHSNGTVKDRSPFRTLMSV  159 
BAH02172/A     129 CYQFALGQGTTLDNKHSNDTIHDRIPHRTLLMN  161 
BAH70320/A     128 CKMYALHQGTTIRNKHSNGTIHDRTAFRGLIST  160 
BAI39637/A     109 CRTFFLTQGALLNDKHSNGTVKDRSPHRTLMSC  141 
BAI48894/A     129 CRTFFLTQGALLNDKHSNGTVKDRSPYRTLMSC  161 
BAI50030/A     129 CWSFALAQGALLGTKHSNGTIKDRTPYRSLIRF  161 
BAJ07979/A     125 CRTFFLTQGALLNDKHSNNTVKDRSPYRALMSV  157 
BAJ10562/A     129 CRMFALSQGTTLRGRHANGTIHDRSPFRALISW  161 
CAA36475/A     107 CRTFFLTQGALLNDKHSNGTVKDRSPYRTLMSC  139 
CAC36997/A     129 CYQFALGQGTTLDNKHSNDTIHDRIPHRTLLMN  161 
CAD57252/A     129 CRTFFLTQGALLNDKHSNGTVKDRSPYRALMSC  161 
CAO82694/A     140 CRTFFLTQGALLNDKHSNGTIKDRSPYRTLMSC  172 
CAP69845/A     128 CRTFFLTQGALLNDKHSNGTVKDRSPFRTLMSC  160 

cons           661 *  : * : :     : ..*   *   * *:    693 


AAA43735/B     160 KLGK-IPTVENS-IFH-MAAWSGSACHDGREWT  189 
AAA43743/B     160 KLGK-IPTVENS-IFH-MAAWSGSACHDGREWT  189 
AAA43749/B     160 KLGK-IPTVENS-IFH-MAAWSGSACHDGREWT  189

AAU94779/B     160 KLGK-IPTVENS-IFH-MAAWSGSACHDGREWT  189

ABL84344/B     160 KLGK-IPTVENS-IFH-MAAWSGSACHDGREWT  189 
ABO72374/B     160 KLGK-IPTVENS-IFH-MAAWSGSACHDGKEWT  189

ABQ81846/B     160 KLGK-IPTVENS-IFH-MAAWSGSACHDGREWT  189

ACU12759/B     160 KLGK-IPTVENS-IFH-MAAWSGSACHDGKEWT  189

BAB32609/B     160 NLGK-IPTVENS-IFH-MAAWSGSACHDGREWT  189

AAA43093/A     159 PLGS-PPTVSNS-EFI-CVGWSSTSCHDGVNRM  188 
AAA43429/A     160 EVGQ-SPNVYQA-RFE-AVAWSATACHDGKKWM  189 
AAA43449/A     162 PIGE-APSPYNS-RFE-SVAWSASACYDGMGCL  191 
AAA43575/A     163 PLSS-PPTVYNS-RVE-CIGWSSTSCHDGRARM  192 
AAD49005/A     162 ELGV-PFNLG-T-KQV-CIAWSSSSCHDGKAWL  190 
AAD49007/A     162 ELGV-PFHLG-T-KQV-CIAWSSSSCHDGKAWL  190 
AAF77036/A     162 PVGE-APSPYNS-RFE-SVAWSASACHDGMGWL  191 
AAO46232/A     162 ELGV-PFHLG-T-RQV-CIAWSSSSCHDGKAWL  190 
AAO46822/A     162 PVGE-APSPYNS-RFE-SVAWSASACHDGTSWL  191 
AAO62036/A     162 PIGTAPV-LG-NYKEI-CIAWSSSSCFDGKEWM  191 
AAO62041/A     162 PVGE-APSPYNS-RFE-SVAWSASACHDGISWL  191 
AAO62044/A     161 PIGV-APSPSNS-RFE-SVAWSATACSDGPGWL  190 
AAO62064/A     162 EMGQ-APSPYNT-RVE-CIGWSSTSCHDGKSRM  191 
AAT08005/A     160 EIGQ-SPNVYQA-RFE-AVAWSATACHDGKKWM  189 
AAT37407/A     162 PIGTAPV-LG-NYKEI-CIAWSSSSCFDGKEWM  191 
AAT37414/A     162 PIGTAPV-LG-NYKEM-CAAWSSSSCFDGKEWL  191 
AAT65406/A     162 EMGQ-APSPYNI-RVE-CVGWSSTSCHDGISRM  191 
AAX78821/A     162 ELGV-PFHLG-T-KQV-CIAWSSSSCHDGRAWL  190 
AAY52584/A     162 ELGV-PFHLA-T-KQV-CIAWSSSSCHDGKAWL  190 
AAY87411/A     162 ELGV-PFHLG-T-KQV-CIAWSSSSCHDGRAWL  190 
ABB17695/A     159 PLGS-SPNAYQA-KFE-SVAWSATACHDGKKWL  188 
ABB19439/A     162 EMGQ-APSPYNV-RVE-CVGWSSTSCHDGISRM  191 
ABB19610/A     162 PVGE-APSPYNS-RFE-SVAWSASACHDGISWL  191 
ABB19747/A     159 PLGS-SPNAYQA-KFE-SVAWSATACHDGKEWL  188 
ABB19914/A     161 PIGV-APSPSNS-RFE-SVAWSATACSDGSGWL  190 
ABB20088/A     161 PIGV-APSPSNS-RFE-SVAWSATACSDGPGWL  190 
ABB20104/A     160 PLGS-PPIVSNS-DFL-CVGWSSTSCHDGIGRM  189 
ABB20243/A     163 PLSS-PPTVYNT-RVE-CIGWSSTSCHDGKARM  192 
ABB20375/A     162 EMGQ-APSPYNA-RVE-CIGWSSTSCHDGKSRM  191 
ABB20475/A     162 ELGV-PFHLG-T-KQV-CIAWSSSSCHDGRAWL  190 
ABB20501/A     159 PLGN-PPTVSNS-EFI-CVGWSSTSCHDGVSRM  188 
ABB21754/A     163 PLSS-PPTVYNT-KIE-CIGWSSTSCHDGKARM  192 
ABB87198/A     159 PLGS-SPNAYQA-KFE-SVAWSATACHDGKEWL  188 
ABB87732/A     161 PIGV-APSPSNS-RFE-SVAWSATACSDGPGWL  190 
ABB87959/A     160 PLGS-PPVVSNS-DFL-CVGWSSTSCHDGIGRM  189 
ABB88058/A     162 PIGTAPV-LG-NYKEI-CIAWSSSSCFDGKEWM  191 
ABB88351/A     162 EMGQ-APSPYNA-RVE-CIGWSSTSCHDGISRM  191 
ABC02269/A     162 ELGV-PFHLG-T-RQV-CIAWSSSSCHDGKAWL  190 
ABF47958/A     146 PVGE-APSPYNS-RFE-SVAWSASACHDGMGWL  175 
ABG37189/A     162 ELGV-PFHLG-T-KQV-CIAWSSSSCHDGKAWL  190 
ABH04381/A     162 PIGTAPV-LG-NYKEI-CIAWSSSSCFDGKEWM  191 
ABI30359/A     162 ELGV-PFHLG-T-KQV-CIAWSSSSCHDGKAWL  190 
ABI84399/A     162 PIGTAPV-LG-NYEEM-CAAWSSSSCFDGKEWM  191 
ABI84436/A     159 PLGS-SPNAYQA-KFE-SVAWSATACHDGKKWL  188 
ABI84475/A     161 PIGV-APSPSNS-RFE-SVAWSATACSDGPGWL  190 
ABI84973/A     162 PIGTAPV-LG-NYKEI-CVAWSSSSCFDGKEWM  191 
ABI84982/A     161 PLGT-PPTVSNS-DFI-CVGWSSTSCHDGVGRM  190 
ABI92217/A     162 EMGQ-APSPYNI-RVE-CVGWSSTSCHDGISRM  191 
ABJ09099/A     160 EIGQ-SPNVYQA-RFE-AVAWSATACHDGKKWM  189 
ABK32099/A     161 HIGV-APSPSNS-RFE-SVAWSATACSDGPGWL  190  
ABP49198/A     163 PLSS-PPTVYNS-RVE-CIGWSSTSCHDGRARM  192 
ABP49253/A     163 PLSS-PPTVYNS-RVE-CIGWSSTSCHDGRARM  192  
ABQ97208/A     162 ELGV-PFHLG-T-KQV-CIAWSSSSCHDGKAWL  190 
ABR28683/A     162 PIGE-APSPYNS-RFE-SVAWSASACHDGMGWL  191 
ABR37723/A     163 PLSS-PPTVYNT-RIE-CIGWSSTSCHDGKARM  192 
ABS50124/A     162 PIGE-APSPYNS-RFE-SVAWSASACHDGMGWL  191 
ABS89357/A     161 HLGT-PPTVSNS-DFI-CVGWSSTSCHDGIGRM  190 
ABU95382/A     162 PVGE-APSPYNS-RFE-SVAWSASACHDGIGWL  191 
ABV25648/A     162 PIGE-APSPYNS-RFE-SVAWSASACHDGMGWL  191 
ABV29593/A     162 PIGE-VPSPYNS-RFE-SVAWSASACHDGISWL  191 
ABV82576/A     162 PIGE-APSPYNS-RFE-SVAWSASACHDGMGWL  191 
ABV82587/A     162 PIGE-APSPYNS-RFE-SVAWSASACHDGMGWL  191 
ABW95951/A     142 PVGE-APSPYNS-RFE-SVAWSASACHDGTSWL  171 
ABX88802/A     162 ELGV-PFHLG-T-KQV-CIAWSSSSCHDGKAWL  190 
ABY84687/A     162 PIGE-APSPYNS-RFE-SVAWSASACHDGMGWL  191 
ACA04655/A     162 EMGQ-APSPYNV-RVE-CIGWSSTSCHDGISRM  191 
ACA04738/A     161 PLGT-PPTVSNS-DFI-CVGWSSTSCHDGVGRM  190 
ACA04739/A     160 KVGQ-SPNVYQA-RFE-AVAWSATACHDGKKWM  189 
ACA04740/A     163 PLSS-PPTVYNS-RVE-CIGWSSTSCHDGKARM  192 
ACA14299/A     162 ELGV-PFHLG-T-KQV-CIAWSSSSCHDGKAWL  190 
ACB70583/A     142 PVGE-APSPYNS-RFE-SVAWSASACHDGTSWL  171 
ACD47117/A     162 ELGV-PFHLG-T-KQV-CIAWSSSSCHDGKAWL  190 
ACD65204/A     162 PIGE-APSPYNS-RFE-SVAWSASACHDGMGWL  191 
ACD76864/A     162 ELGV-PFHLG-T-KQV-CIAWSSSSCHDGKAWL  190 
ACD85421/A     160 EVGQ-SPNVYQA-RFE-AVAWSATACHDGKKWM  189 
ACE73320/A     161 PIGV-APSPSNS-RFE-SVAWSATACSDGPGWL  190 
ACE76594/A     162 ELGV-PFHLG-T-KQV-CIAWSSSSCHDGKAWL  190 
ACE78976/A     161 GMGQ-APSPYNI-KIE-CIGWSSTSCHDGISRM  190 
ACE78981/A     161 EMGQ-APSPYNV-RVE-CIGWSSTSCHDGISRM  190 
ACF20219/A     160 PLGS-PPIVSNS-DFL-CVGWSSTSCHDGIGRM  189 
ACF25054/A     162 ELGV-PFHLG-T-KQV-CIAWSSSSCHDGKAWL  190 
ACF25354/A     160 PLGS-PPIVSNS-DFL-CVGWSSTSCHDGIGRM  189 
ACF33655/A     162 PIGTAPV-LG-NYKEI-CIAWSSSSCFDGKEWM  191 
ACF36500/A     162 ELGV-PFHLG-T-KQV-CIAWSSSSCHDGKAWL  190 
ACF93490/A     160 KIGQ-SPNVYQA-RFE-SVAWSATACHDGKKWM  189 
ACI41114/A     162 PIGTAPV-LG-NYKEI-CIAWSSSSCFDGKEWM  191 
ACI48786/A     160 KIGQ-SPNVYQA-RFE-SVAWSATACHDGKKWM  189 
ACN65661/A     161 PLGT-PPTVSNS-DFI-CVGWSSTTCHDGVGRM  190 
ACO24985/A     161 PVGE-VPSPYNS-RFE-SVAWSASACHDGISWL  190 
ACP50710/A     162 ELGV-PFNLG-T-KQV-CIAWSSSSCHDGRAWL  190 
ACQ73415/A     159 PLGS-PPTVSNS-EFI-CVGWSSTSCHDGVSRM  188 
ACR66907/A     162 PIGTAPV-LG-NYKEI-CVAWSSSSCFDGKEWM  191 
ACR83968/A     162 PIGE-VPSPYNS-RFE-SVAWSASACHDGTSWL  191 
ACS68311/A     163 PLSS-PPTVYNS-RVE-CIGWSSTSCHDGKARM  192 
ACS92897/A     162 ELGV-PFHLG-T-RQV-CIAWSSSSCHDGKAWL  190 
ACT67761/A     162 ELGV-PFHLG-T-KQV-CIAWSSSSCHDGKAWL  190 
ACT97062/A     160 EVGQ-SPNVYQA-RFE-AVAWSATACHDGKKWM  189 
ACV41710/A     160 EIGQ-SPNVYQA-RFE-AVAWSATACHDGKKWM  189 
ADA71169/A     162 PLGE-APSPYNS-KFE-SVAWSASACHDGIGWL  191 
ADK70163/A     161 PIGV-APSPSNS-RFE-SVAWSATACSDGPGWL  190 
ADU17627/A     163 PLSS-PPTVYNS-RVE-CIGWSSTSCHDGRARM  192 
ADU20276/A     159 PLGS-SPNAYQA-RFE-SVAWSATACHDGKEWL  188 
ADU20397/A     162 PIGTAPV-LG-NYKEI-CIAWSSSSCFDGKEWM  191 
ADU53220/A     161 PIGV-APSPSNS-RFE-SVAWSATACSDGPGWL  190 
AEB89856/A     163 PLSS-PPTVYNS-RVE-CIGWSSTSCHDGKSRM  192 
AEB89867/A     160 EVGQ-SPNVYQA-RFE-AVAWSATACHDGKKWM  189 
AEK50939/A     162 EMGQ-APSPYNV-RVE-CIGWSSTSCHDGISRM  191 
AEK50961/A     160 PLGS-PPVVSNS-DFL-CVGWSSTSCHDGIGRM  189 
BAA06132/A     162 ELGV-PFHLG-T-RQV-CIAWSSSSCHDGKAWL  190 
BAA06718/A     162 PIGE-APSPYNS-RFE-SVAWSASACHDGMGWL  191 
BAF03532/A     158 PLGS-SPNAYQA-KFE-SVGWSATACHDGKEWM  187 
BAF03569/A     158 PLGS-SPNAYQA-KFE-SVGWSATACHDGKEWM  187 
BAF34378/A     162 ELGV-PFHLG-T-KQV-CIAWSSSSCHDGKAWL  190 
BAF34925/A     162 ELGV-PFHLG-T-KQV-CIAWSSSSCHDGKAWL  190 
BAF43417/A     159 PLGS-SPNAYQA-KFE-SVAWSATACHDGKKWL  188 
BAF43457/A     160 EVGQ-SPNVYQA-RFE-AVAWSATACHDGKKWM  189 
BAF43461/A     158 PLGS-SPNAYQA-KFE-SVGWSATACHDGKEWM  187 
BAF46763/A     162 PIGTAPV-LG-NYKEI-CVAWSSSSCFDGKGWM  191 
BAF46905/A     162 EMGQ-APSPYNV-RVE-CIGWSSTSCHDGISRM  191 
BAF48640/A     162 ELGV-PFHLG-T-KQV-CIAWSSSSCHDGKAWL  190 
BAF48644/A     162 PIGTAPV-LG-NYKEI-CVAWSSSSCFDGKEWM  191 
BAF48646/A     161 PLGT-PPTVSNS-DFI-CVGWSSTTCHDGIGRM  190 
BAF63047/A     162 PLGE-APSPYNS-KFE-SVAWSASACHDGMGWL  191 
BAG66251/A     160 GIGQ-SPNVYQA-RFE-AVAWSATACHDGKKWM  189 
BAG66259/A     162 PIGT-APVLG-N-YKEICIAWSSSSCFDGKEWM  191 
BAG66279/A     160 EVGQ-SPNVYQA-RFE-AVAWSATACHDGKKWM  189 
BAH02172/A     162 ELGV-PFHLG-T-RQV-CIAWSSSSCHDGKAWL  190 
BAH70320/A     161 PLGT-PPTVSNS-DFM-CVGWSSTTCHDGVGRM  190 
BAI39637/A     142 PVGE-APSPYNS-RFE-SVAWSASACHDGTSWL  171 
BAI48894/A     162 PVGE-APSPYNS-RFE-SVAWSASACHDGISWL  191 
BAI50030/A     162 PIGTAPV-LG-NYKEI-CVAWSSSSCFDGKEWM  191 
BAJ07979/A     158 PLGS-SPNAYQA-KFE-SVGWSATACHDGKEWM  187 
BAJ10562/A     162 EMSQ-APSPYNT-KVE-CIGWSSTSCHDGISRM  191 
CAA36475/A     140 PVGE-APSPYNS-RFV-SVAWSASACHDGMGWL  169 
CAC36997/A     162 ELGV-PFHLG-T-RQV-CIAWSSSSCHDGKAWL  190 
CAD57252/A     162 PLGE-APSPYNS-KFE-SVAWSASACHDGMGWL  191 
CAO82694/A     173 PIGE-VPSPYNS-RFE-SVAWSASACHDGTSWL  202 
CAP69845/A     161 PIGV-APSPSNS-RFE-SVAWSATACSDGPGWL  190 

cons           694  :.                .**.::* **      726 

AAA43735/B     190 YIGVDGPDSDALIKIKYGEAYTDT-YHSYAHNI  221 
AAA43743/B     190 YIGVDGPDSNALIKIKYGEAYTDT-YHSYANNI  221 
AAA43749/B     190 YIGVDGPDNDALVKIKYGEAYTDT-YHSYAHNI  221

AAU94779/B     190 YIGVDGPDSNALLKIKYGEAYTDT-YHSYANNI  221

ABL84344/B     190 YIGVDGPDSNALLKIKYGEAYTDT-YHSYANNI  221 
ABO72374/B     190 YIGVDGPDSNALLKIKYGEAYTDT-YHSYANNI  221

ABQ81846/B     190 YIGVDGPDSNALIKIKYGEAYTDT-YHSYANNI  221

ACU12759/B     190 YIGVDGPDNNALLKIKYGEAYTDT-YHSYANNI  221

BAB32609/B     190 YIGVDGPDSNALIKIKYGEAYTDT-YHSYANNI  221

AAA43093/A     189 TICVQGDNENATATVYYNKRLTTT-IKTWAKNI  220 
AAA43429/A     190 TVGVTGPDAQAVAVVHYGGVPVDV-INSWAGNI  221 
AAA43449/A     192 TIGISGPDDGAVAVLKYNGIITET-IKSWRKQI  223 
AAA43575/A     193 SICISGPNNNASAVIWYNRRPVTE-INTWARNI  224 
AAD49005/A     191 HVCVTGDDRNATAKFIYNGMLVDS-IGSWSQNI  222 
AAD49007/A     191 HVCVTGDDRNATASIIYDGMLVDS-IGSWSKNI  222 
AAF77036/A     192 TIGISGPDNGAVAVLKYNGIITDT-IKSWRNNI  223 
AAO46232/A     191 HVCVTGDDKNATASFIYDGRLVDS-IGSWSQNI  222 
AAO46822/A     192 TIGISGPDNGAVAVLKYNGIITDT-IKSWRNNI  223 
AAO62036/A     192 HVCMTGNDNDASAQIIYAGKMTDS-IKSWRRDI  223 
AAO62041/A     192 TIGISGPDNGAVAVLKYNGIITDT-IKNWRNNI  223 
AAO62044/A     191 TIGITGPDATAVAVLKYNGIITDT-LKSWKGNI  222 
AAO62064/A     192 SICMSGPNNNASAVVWYGGRPATE-IPSWAGNI  223 
AAT08005/A     190 TIGVTGPDAKAVAVVHYGGIPTDV-INSWAGDI  221 
AAT37407/A     192 HVCMTGNDNDASGQIIYAGRMTDS-IKSWRKDI  223 
AAT37414/A     192 HVCVTGNDNDATAQIIYAGKMRDS-IKSWRKNI  223 
AAT65406/A     192 SICMSGPNNNASAVVWYNGRPVTE-IASWAGNI  223 
AAX78821/A     191 HICVTGDDRNATASFIYDGRLVDS-IGSWSQNI  222 
AAY52584/A     191 HVCVTGDDRNATASIIYDGILVDS-IGSWSKNI  222 
AAY87411/A     191 HVCVTGDDRNATASLIYDGRLVDS-IGSWSQNI  222 
ABB17695/A     189 AIGISGADDDAYAVIHYGGMPTDV-VRSWRKQI  220 
ABB19439/A     192 SICMSGPNNNASAVVWYNGRPVTE-IASWAGNI  223 
ABB19610/A     192 TIGISGPDNGAVAVLKYNGIITDT-IKSWRNNI  223 
ABB19747/A     189 AVGVSGADDDAYAVIHYGGMPTDV-VRSWRKQI  220 
ABB19914/A     191 TLGITGPDATAVAVLKYNGIITDT-LKSWKGNI  222 
ABB20088/A     191 TLGITGPDATAVAVLKYNGIITDT-LKSWKGNI  222 
ABB20104/A     190 TICVQGNNDNATATVYYDRRLTTT-IKTWAGNI  221 
ABB20243/A     193 SICVSGPNNNASAVVWYNGRPITE-INTWARNI  224 
ABB20375/A     192 SICMSGPNNNASAVVWYGGRPVTE-IPSWAGNI  223 
ABB20475/A     191 HVCVTGDDRNATASLIYDGRLVDS-IGSWSQNI  222 
ABB20501/A     189 TICVQGNNENATATVYYNKRLTTT-IKTWAKNI  220 
ABB21754/A     193 SICVSGPNNNASAVIWYNGRPTTE-INTWARNI  224 
ABB87198/A     189 AVGISGADDDAYAVIHYGGVPTDV-VRSWRKQI  220 
ABB87732/A     191 TLGITGPDATAVAVLKYNGIITDT-LKSWKGNI  222 
ABB87959/A     190 TICVQGNNDNATATVYYDRRLTTT-IKTWARNI  221 
ABB88058/A     192 HVCMTGNDNDASAQIIYAGRMTDS-IKSWRKDI  223 
ABB88351/A     192 SICMSGPNNNASAVVWYGGRPVTE-IPSWAGNI  223 
ABC02269/A     191 HVCVTGYDKNATASFIYDGRLVDS-IGSWSQNI  222 
ABF47958/A     176 TIGISGPDDGAVAVLKYNGIITET-IKSWRKNI  207 
ABG37189/A     191 HVCVTGHDENATASFIYDGRLVDS-IGSWSKNI  222 
ABH04381/A     192 HVCMTGNDNDASAQIIYAGRMTDS-IKSWRKDI  223 
ABI30359/A     191 HVCVTGDDRNATASFIYDGMLVDS-ISSWSQNI  222 
ABI84399/A     192 HVCITGNDNDATAQIIYAGTMRDS-IKSWRRNI  223 
ABI84436/A     189 AVGISGADDDAYAVIHYGGMPTDV-VRSWRKQI  220 
ABI84475/A     191 TLGITGPDTTAVAVLKYNGVITDT-LKSWKGNI  222 
ABI84973/A     192 HVCMTGNDNDASAQIIYAGKMTDS-IKSWRRDI  223 
ABI84982/A     191 TICIQGNNDNATATVYYNRRLTTT-IKTWAKNI  222 
ABI92217/A     192 SICMSGPNNNASAVVWYNGRPVTE-IASWAGNI  223 
ABJ09099/A     190 TIGVTGPDAKAVAVVHYGGIPTDV-INSWAGDI  221 
ABK32099/A     191 TLGITGPDSTAVAVIKYNGIITDT-LKSWKGNI  222  
ABP49198/A     193 SICISGPNNNASAVIWYNRRPVTE-INTWARNI  224 
ABP49253/A     193 SICISGPNNNASAVIWYNRRPVTE-INTWARNI  224  
ABQ97208/A     191 HVCITGHDKNATASFIYDGRLVDS-IVSWSKNI  222 
ABR28683/A     192 TIGISGPDNGAVAVLKYNGIITDT-IKSWRSKI  223 
ABR37723/A     193 SICVSGPNNNASAVIWYNGRPTTE-INTWARNI  224 
ABS50124/A     192 TIGISGPDNGAVAVLKYNGIITDT-IKSWRNKI  223 
ABS89357/A     191 TICVQGNNDNATATVYYNRRLTTT-IKTWARNI  222 
ABU95382/A     192 TIGISGPDNGAVAVLKYNGIITDT-IKSWRNDI  223 
ABV25648/A     192 TIGISGPDNGAVAVLKYNGIITDT-IKSWRNKI  223 
ABV29593/A     192 TIGISGPDNGAVAVLKYNGIITDT-IKSWRNNI  223 
ABV82576/A     192 TIGISGPDNGAVAVLKYNGIITDT-IKSWRNKI  223 
ABV82587/A     192 TIGISGPDNGAVAVLKYNGIITDT-IKSWRNKI  223 
ABW95951/A     172 TIGISGPDNGAVAVLKYNGIITDT-IKSWRNNI  203 
ABX88802/A     191 HVCVTGDDRNATASFIYDGMLVDS-IGSWSQNI  222 
ABY84687/A     192 TIGISGPDNGAVAVLKYNGIITDT-IKSWRNKI  223 
ACA04655/A     192 SICMSGPNNNASAVVWYGGRPVTE-IPSWAGNI  223 
ACA04738/A     191 IICIQGNNDNATATVYYNRRLTTT-IKPWARNI  222 
ACA04739/A     190 TVGVTGPDSKAVAVIHYGGVPTDV-INSWAGDI  221 
ACA04740/A     193 SICISGPNNNASAVIWYNRRPVTE-INTWARNI  224 
ACA14299/A     191 HVCVTGDDRNATASFIYNGMLIDS-IVSWSQNI  222 
ACB70583/A     172 TIGISGPDNGAVAVLKYNGIITDT-IKSWGNNI  203 
ACD47117/A     191 HVCVTGDDGNATASIIYDGMLTDS-IGSWSKSI  222 
ACD65204/A     192 TIGISGPDNGAVAVLKYNGIITDT-IKSWRNKI  223 
ACD76864/A     191 HVCVTGDDRNATASFIYDGVLVDS-IGSWSQNI  222 
ACD85421/A     190 TVGVTGPDAQAVAVVHYGGVPVDV-INSWAGDI  221 
ACE73320/A     191 TLGITGPDATAVAVLKYNGIITDT-LKSWKGNI  222 
ACE76594/A     191 HVCVTGDDKNATASFIYDGMLVDS-IGSWSQNI  222 
ACE78976/A     191 SICMSGPNNNASAVVWYGGRPVTE-IPSWAGNI  222 
ACE78981/A     191 SICISGPNNNASAVVWYEGRPVTE-IPSWAGNI  222 
ACF20219/A     190 TICVQGNNDNATATVYYDRRLTTT-IKTWAGNI  221 
ACF25054/A     191 HVCVTGDDRNATASFIYDGMLVDS-IGSWSQNI  222 
ACF25354/A     190 TICVQGNNDNATATVYYDRRLTTT-IKTWAGNI  221 
ACF33655/A     192 HVCMTGNDNDASAQIIYAGRMTDS-IKSWRKDI  223 
ACF36500/A     191 HVCVTGDDENATASFIYNGRLVDS-VVSWSKKI  222 
ACF93490/A     190 TVGVTGPDNQAIAVVNYGGVPIDI-INSWAGDI  221 
ACI41114/A     192 HVCMTGNDNDASAQIIYAGRMTDS-IKSWRKDI  223 
ACI48786/A     190 TVGVTGPDNQAIAVVNYGGVPVDI-INSWAWDI  221 
ACN65661/A     191 TICIQGNNDNATATVYYNRRLTTT-IKTWARNI  222 
ACO24985/A     191 TIGISGPDNGAVAVLKYNGIITDT-IKSWRNNI  222 
ACP50710/A     191 HVCITGDDRNATASIIYDGMITDS-IGSWSNNI  222 
ACQ73415/A     189 TICVQGDNENATATVYYNKRLTTT-IKTWAKNI  220 
ACR66907/A     192 HVCMTGNDNDASGQIMYAGKMTDS-IKSWRKDI  223 
ACR83968/A     192 TIGISGPDNGAVAVLKYNGIITDT-IKSWRKNI  223 
ACS68311/A     193 SICISGPNNNASAVIWYNRRPVTE-INTWARNI  224 
ACS92897/A     191 HVCITGDDKNATASFIYNGRLVDS-IGSWSKNI  222 
ACT67761/A     191 HVCITGDDKNATASFIYNGRLVDS-VVSWSKEI  222 
ACT97062/A     190 TVGVTGPDSKAVAVVHYGGVPTDV-INSWAGDI  221 
ACV41710/A     190 TIGVTGPDAKAVAVVHYGGIPTDV-INSWAGDI  221 
ADA71169/A     192 TIGISGPDNGAVAVLKYKGIITGT-IKSWKKQI  223 
ADK70163/A     191 TLGITGPDSTAVAVIKYNGIITDT-FKSWKGNI  222 
ADU17627/A     193 SICISGPNNNASAVIWYNRRPVTE-INTWARNI  224 
ADU20276/A     189 AVGISGADDDAYAVIHYGGMPTDV-VRSWRKQI  220 
ADU20397/A     192 HVCMTGNDNDASAQIIYGGRMTDS-IKSWRKDI  223 
ADU53220/A     191 TLGITGPDATAVAVLKYNGIITDT-LKSWKGNI  222 
AEB89856/A     193 SICISGPNNNASAVVWYNRRPVAE-INTWARNI  224 
AEB89867/A     190 TVGVTGPDSKAVAVVHYGGVPTDV-INSWAGDI  221 
AEK50939/A     192 SICMSGPNNNASAVVWYNGRPVTE-IASWAGNI  223 
AEK50961/A     190 TICVQGNNDNATATVYYDRRLTTT-IKTWAGNI  221 
BAA06132/A     191 HVCVTGDDKNATASFIYNGRLVDS-IGSWSQNI  222 
BAA06718/A     192 TIGISGPDDGAVAVLKYNGIITET-IKSWRKRI  223 
BAF03532/A     188 AIGVSGADDDAYAVIHYGGVPTDV-VRSWRKQI  219 
BAF03569/A     188 AIGVSGADDDAYAVIHYGGVPTDV-VRSWRKQI  219 
BAF34378/A     191 HVCVTGDDRNATASFIYNGMLVDS-IGSWSQNI  222 
BAF34925/A     191 HVCVTGDDRNATASFIYDGMLVDS-IGSWSQNI  222 
BAF43417/A     189 AVGISGADDDAYAVIHYGGMPTDV-VRSWRKQI  220 
BAF43457/A     190 TIGVTGPDSKAVAVVHYGGVPTDV-VNSWAGDI  221 
BAF43461/A     188 AIGVSGADDDAYAVIHYGGVPTDV-VRSWRKQI  219 
BAF46763/A     192 HVCMTGNDNDASAQIIYAGRMTDS-IKSWRRDI  223 
BAF46905/A     192 SICMSGPNNNASAVVWYGGRPVTE-IPSWAGNI  223 
BAF48640/A     191 HVCITGDDRNATASFIYDGMLVDS-IGSWSQNI  222 
BAF48644/A     192 HVCMTGNDNDASAQIIYAGKMTDS-IKSWRRDI  223 
BAF48646/A     191 TICIQGNNDNATATVYYNRRLTTT-IKTWARNI  222 
BAF63047/A     192 TIGISGPDNGAVAVLKYNGIITET-IKSWKKRI  223 
BAG66251/A     190 IIGVTGPDAKAVAVVHYGGIPTDV-INSWAGDI  221 
BAG66259/A     192 HVCMTGNDNDASAQIIYAGR-MTDSIKSWRKDI  223 
BAG66279/A     190 TIGVTGSDSKAVAVVHYGGVPTDV-VNSWAGDI  221 
BAH02172/A     191 HVCVTGDDKNATASFIYNGRLVDS-IGSWSQNI  222 
BAH70320/A     191 TICIQGNNDNATATVYYNGRLTTT-IKTWARNI  222 
BAI39637/A     172 TIGISGPDNGAVAVVKYNGIITDT-IKSWRNNI  203 
BAI48894/A     192 TIGISGPDTGAVAVLKYNGIITDT-IKSWRNNI  223 
BAI50030/A     192 HVCMTGNDNDASGQIIYAGKMTGS-IKSWRKDI  223 
BAJ07979/A     188 AIGVSGADDDAYAVIHYGGIPTDV-VRSWRKQI  219 
BAJ10562/A     192 SICMSGPNNNASAVVWYGGRPVTE-IPSWAGNI  223 
CAA36475/A     170 TIGISGPDNGAVAVLKYNGIITDT-IKSWKNNI  201 
CAC36997/A     191 HVCVTGDDKNATASFIYDGRLVDS-IGSWSQNI  222 
CAD57252/A     192 TIGISGPDNGAVAVLKYNGIITET-IKSWKKRI  223 
CAO82694/A     203 TIGISGPDNGAVAVLKYNGIITDT-IKSWRSNI  234 
CAP69845/A     191 TLGITGPDSTAVAVIKYNGIITDT-LKSWKGNI  222 

cons           727  : : * :  *   . *           :   *  759 


AAA43735/B     222 LRTQESACNCIGGDCYLMITDGSASGISKCRFL  254 
AAA43743/B     222 LRTQESACNCIGGDCYLMITDGSASGISKCRFL  254 
AAA43749/B     222 LRTQESACNCIGGDCYLMITDGSASGISKCRFL  254

AAU94779/B     222 LRTQESACNCIGGDCYLMITDGSASGISECRFL  254

ABL84344/B     222 LRTQESACNCIGGDCYLMITDGSASGVSECRFL  254 
ABO72374/B     222 LRTQESACNCIGGNCYLMITDGSASGISECRFL  254

ABQ81846/B     222 LRTQESACNCIGGDCYLMITDGSASGISKCRFL  254

ACU12759/B     222 LRTQESACNCIGGNCYLMITDGSASGVSECRFL  254

BAB32609/B     222 LRTQESACNCIGGDCYLMITDGSASGISKCRFL  254     
AAA43093/A     221 LRTQESECVCHNSTCVVVMTDGPANNQAFTKVI  253 
AAA43429/A     222 LRTQESSCTCIKGDCYWVMTDGPANRQAQYKIF  254 
AAA43449/A     224 LRTQESECVCVNGSCFTIMTDGPSDGPASYRIF  256 
AAA43575/A     225 LRTQESECVCQNGVCPVVFTDGSATGPAETRIY  257 
AAD49005/A     223 LRTQESECVCINGTCTVVMTDGSASGRADTRIL  255 
AAD49007/A     223 LRTQESECVCINGTCTVVMTDGSASGRADTRIL  255 
AAF77036/A     224 LRTQESECACVNGSCFTIMTDGPSNGQASYKIL  256 
AAO46232/A     223 LRTQESECVCINGTCTVVMTDGSASGRADTRIL  255 
AAO46822/A     224 LRTQESECACVNGSCFTVMTDGPSNGQASYKIF  256 
AAO62036/A     224 LRTQESECQCIDGTCVVAVTDGPAANSADHRVY  256 
AAO62041/A     224 LRTQESECACVNGSCFTVMTDGPSNGQASYKIF  256 
AAO62044/A     223 MRTQESECVCQDEFCYTLITDGPSDAQAFYKIL  255 
AAO62064/A     224 LRTQESECVCHNGVCPVVMTDGPANNRAATKII  256 
AAT08005/A     222 LRTQESSCTCIQGECYWVMTDGPANRQAQYRAF  254 
AAT37407/A     224 LRTQESECQCIDGTCVVAVTDGPAANSADHRIY  256 
AAT37414/A     224 LRTQESECQCLYGTCVVAVTDGPADNKADHRVY  256 
AAT65406/A     224 LRTQESECVCHNGICPVVMTDGPANNRAETKII  256 
AAX78821/A     223 LRTQESECVCINGTCTVVMTDGSASGRADTRIL  255 
AAY52584/A     223 LRTQESECVCINGTCAVVMTDGSASGRADTRIL  255 
AAY87411/A     223 LRTQESECVCINGTCTVVMTDGSASGKADTRIL  255 
ABB17695/A     221 LRTQESSCVCMNGDCYWVMTDGPANNQASYKIF  253 
ABB19439/A     224 LRTQESECVCHNGICPVVMTDGPANNRAETKII  256 
ABB19610/A     224 LRTQESECACINGSCFTIMTDGPSNGQASYKIF  256 
ABB19747/A     221 LRTQESSCVCMKGNCYWVMTDGPANNQASYKIF  253 
ABB19914/A     223 MRTQESECVCQDEFCYTLITDGPSDAQAFYKIL  255 
ABB20088/A     223 MRTQESECVCQDEFCYTLITDGPSDAQAFYKIL  255 
ABB20104/A     222 LRTQESECVCHNGTCVAVMTDGSASSQAYTKVL  254 
ABB20243/A     225 LRTQESECVCHNGICPVVFTDGSATGPAETRIY  257 
ABB20375/A     224 LRTQESECVCHNGVCPVVMTDGPANNRAATKVI  256 
ABB20475/A     223 LRTQESECVCINGTCTVVMTDGSASGRADTRIL  255 
ABB20501/A     221 LRTQESECVCHNSTCVVVMTDGPANNQAFTKVI  253 
ABB21754/A     225 LRTQESECVCHNGICPVVFTDGSATGPAETRIY  257 
ABB87198/A     221 LRTQESSCVCMKGNCYWVMTDGPANNQASYKIF  253 
ABB87732/A     223 MRTQESECVCQDEFCYTLITDGPSDAQAFYKIL  255 
ABB87959/A     222 LRTQESECVCHNGTCVVVMTDGSASSQAHTKVL  254 
ABB88058/A     224 LRTQESECQCISGTCVVAVTDGPAANSADHRVY  256 
ABB88351/A     224 LRTQESECVCHKGVCPVVMTDGPANNRAATKII  256 
ABC02269/A     223 LRTQESECVCINGTCTVVMTDGSASGRADTKIL  255 
ABF47958/A     208 LRTQESECTCVNGSCFTIMTDGPSDGLASYKIF  240 
ABG37189/A     223 LRTQESECVCINGTCTVVMTDGSASGRADTKIL  255 
ABH04381/A     224 LRTQESECQCIGGTCVVAVTDGPAANSADHRVY  256 
ABI30359/A     223 LRTQESECVCINGTCTVVMTDGSASGKADTRIL  255 
ABI84399/A     224 LRTQESECQCLHGTCVVAVTDGPADNKADHRVY  256 
ABI84436/A     221 LRTQESSCVCMNGNCYWVMTDGPANKQASYKIF  253 
ABI84475/A     223 MRTQESECVCQDEFCYTLVTDGPSDAQAFYKIL  255 
ABI84973/A     224 LRTQESECQCIDGTCVVAVTDGPAANSADHRVY  256 
ABI84982/A     223 LRTQESECVCHNGTCAVVMTDGPASSQAYTKIM  255 
ABI92217/A     224 LRTQESECVCHNGICPVVMTDGPANNRAETKII  256 
ABJ09099/A     222 LRTQESSCTCIQGECYWVMTDGPANRQAQYRAF  254 
ABK32099/A     223 MRTQESECVCQDEFCYTLITDGPSDAQAFYKIL  255 
ABP49198/A     225 LRTQESECVCHNGVCPVVFTDGSATGPAETRIY  257 
ABP49253/A     225 LRTQESECVCHNGVCPVVFTDGSATGPAETRVY  257 
ABQ97208/A     223 LRTQESECVCINGTCTVVMTDGSASGRADTKIL  255 
ABR28683/A     224 LRTQESECVCINGSCFTIMTDGPSNGQASYKIF  256 
ABR37723/A     225 LRTQESECVCHNGICPVVFTDGSATGPAETRIY  257 
ABS50124/A     224 LRTQESECVCINGSCFTIMTDGPSNGQASYKIF  256 
ABS89357/A     223 LRTQESECVCHNGTCAVVMTDGSASSQAYTKVM  255 
ABU95382/A     224 LRTQESECACINGSCFTIMTDGPSNGQASYKIF  256 
ABV25648/A     224 LRTQESECVCINGSCFTIMTDGPSNGQASYKIF  256 
ABV29593/A     224 LRTQESECACVNGSCFTVMTDGPSNGQASYKIF  256 
ABV82576/A     224 LRTQESECVCINGSCFTIMTDGPSNGQASYKIF  256 
ABV82587/A     224 LRTQESECVCINGSCFTIMTDGPSNGQASYKIF  256 
ABW95951/A     204 LRTQESECACVNGSCFTVMTDGPSNGQASYKIF  236 
ABX88802/A     223 LRTQESECVCINGTCTVVMTDGSASGRADTRIL  255 
ABY84687/A     224 LRTQESECVCINGSCFTIMTDGPSNGQASYKIF  256 
ACA04655/A     224 LRTQESECVCHKGICPVVMTDGPANNRAATKII  256 
ACA04738/A     223 LRTQESECVCHNGTCAVVMTDGSASSQAYTKVM  255 
ACA04739/A     222 LRTQESSCTCIQGDCYWVMTDGPANRQAQYRIY  254 
ACA04740/A     225 LRTQESECVCHNGVCPVVFTDGSATGPAETRIY  257 
ACA14299/A     223 LRTQESECVCINGTCTVVMTDGSASGRADTRVL  255 
ACB70583/A     204 LRTQESECACVNGSCFTVMTDGPSNGQASHKIF  236 
ACD47117/A     223 LRTQESECVCINGTCTVVMTDGSASGRADTRIL  255 
ACD65204/A     224 LRTQESECVCINGSCFTIMTDGPSNGQASYKIF  256 
ACD76864/A     223 LRTQESECVCISGTCAVVMTDGSASGKADTRIL  255 
ACD85421/A     222 LRTQESSCTCIKGDCYWVMTDGPANRQAQYRIF  254 
ACE73320/A     223 MRTQESECVCQDEFCYTLITDGPSDAQAFYKIL  255 
ACE76594/A     223 LRTQESECVCINGTCTVVMTDGSASGRADTRIL  255 
ACE78976/A     223 LRTQESECVCHKGICPVVMTDGPANNKAATKII  255 
ACE78981/A     223 LRTQESECVCHKGICPVVMTDGPANNRAATKII  255 
ACF20219/A     222 LRTQESECVCHNGTCVVIMTDGSASSQAYTKVL  254 
ACF25054/A     223 LRTQESECVCINGTCTVVMTDGSASGRADTRIL  255 
ACF25354/A     222 LRTQESECVCHNGTCVVIMTDGSASSQAHTKVL  254 
ACF33655/A     224 LRTQESECQCIDGTCVVAVTDGPAANSADHRVY  256 
ACF36500/A     223 LRTQESECVCINGTCTVVMTDGSASGKADTKIL  255 
ACF93490/A     222 LRTQESSCTCIKGDCYWVMTDGPANRQAKYRIF  254 
ACI41114/A     224 LRTQESECQCIDGTCVVAVTDGPAANSADHRVY  256 
ACI48786/A     222 LRTQESSCTCIKGDCYWVMTDGPANRQAKYRIF  254 
ACN65661/A     223 LRTQDSECVSHNGTWAVVMTDGSASSQAYTKIM  255 
ACO24985/A     223 LRTQESECACVNGSCFTVMTDGPSNGQASYKIF  255 
ACP50710/A     223 LRTQESECVCINGTCTVVMTDGSASGMADTRIL  255 
ACQ73415/A     221 LRTQESECVCHNSTCVVVMTDGPANNQAFTKVI  253 
ACR66907/A     224 LRTQESECQCIDGTCVVAVTDGPAANSADHRIY  256 
ACR83968/A     224 LRTQESECACINGSCFTVMTDGPSNGQASYKVF  256 
ACS68311/A     225 LRTQESECVCHNGVCPVVFTDGSATGPAETRVY  257 
ACS92897/A     223 LRTQESECVCINGTCTVVMTDGSASGKADTKIL  255 
ACT67761/A     223 LRTQESECVCINGTCTVVMTDGSASGKADTKIL  255 
ACT97062/A     222 LRTQESSCTCIQGNCYWVMTDGPANRQAQYRIY  254 
ACV41710/A     222 LRTQESSCTCIQGECYWVMTDGPANRQAQYRAF  254 
ADA71169/A     224 LRTQESECVCINGSCFTIMTDGPSDGAASYKIF  256 
ADK70163/A     223 MRTQESECVCQDEFCYTLITDGPSDAQAFYKIL  255 
ADU17627/A     225 LRTQESECVCHNGVCPVVFTDGSATGPAETRVY  257 
ADU20276/A     221 LRTQESSCVCVKGHCYWVMTDGPANNQASYKIF  253 
ADU20397/A     224 LRTQESECQCIDGTCVVAVTDGPAANSADHRVY  256 
ADU53220/A     223 MRTQESECVCQDEFCYTLITDGPSDAQAFYKIL  255 
AEB89856/A     225 LRTQESECVCHNGVCPVVFTDGSATGPADTRIY  257 
AEB89867/A     222 LRTQESSCTCIQGDCYWVMTDGPANRQAQYRIY  254 
AEK50939/A     224 LRTQESECVCHNGICPVVMTDGPANNRAETKII  256 
AEK50961/A     222 LRTQESECVCHNGTCVVIMTDGSASSQAYTKVL  254 
BAA06132/A     223 LRTQESECVCINGTCTVVMTDGSASGRADTRIL  255 
BAA06718/A     224 LRTQESECVCVNGSCFTIMTDGPNNGPASYRIF  256 
BAF03532/A     220 LRTQESSCVCMKGECYWVMTDGPANNQASYKIF  252 
BAF03569/A     220 LRTQESSCVCMKGECYWVMTDGPANNQASYKIF  252 
BAF34378/A     223 LRTQESECVCINGICTVVMTDGSASGRADTRIL  255 
BAF34925/A     223 LRTQESECVCINGTCTVVMTDGSASGRADTRIL  255 
BAF43417/A     221 LRTQESSCVCMNGNCYWVMTDGPANSQASYKIF  253 
BAF43457/A     222 LRTQESSCTCIQGNCYWVMTDGPANRQAQYRIY  254 
BAF43461/A     220 LRTQESSCVCMKGECYWVMTDGPANNQASYKIF  252 
BAF46763/A     224 LRTQESECQCIDGTCIVAVTDGPAANSADHRIY  256 
BAF46905/A     224 LRTQESECVCHKGICPVVMTDGPANNRAATKII  256 
BAF48640/A     223 LRTQESECVCINGTCTVVMTDGSASGRADTKIL  255 
BAF48644/A     224 LRTQESECQCIDGTCIVAVTDGPAANSADHRIY  256 
BAF48646/A     223 LRTQESECVCHNGTCAVVMTDGSASSQAYTKIM  255 
BAF63047/A     224 LRTQESECVCVNGSCFTIMTDGPSNGAASYKIF  256 
BAG66251/A     222 LRTQESSCTCIQGECYWVMTDGPANRQAQYRAF  254 
BAG66259/A     224 LRTQESECQCIGGICVVAVTDGPAANSADHRVY  256 
BAG66279/A     222 LRTQESSCTCIQGNCYWVMTDGPANRQAQYRIY  254 
BAH02172/A     223 LRTQESECVCINGTCTVVMTDGSASGRADTRIL  255 
BAH70320/A     223 LRTQESECVCHNGTCAVVMTDGSASSQAYTKVM  255 
BAI39637/A     204 LRTQESECACVNGSCFTVMTDGPSNGQASYKIF  236 
BAI48894/A     224 LRTQESECACVNGSCFTVMTDGPSNGQASYKIF  256 
BAI50030/A     224 LRTQESECQCIDGTCVVAVTDGPAANSADHRIY  256 
BAJ07979/A     220 LRTQESSCVCMKGECYWVMTDGPANNQASYKIF  252 
BAJ10562/A     224 LRTQESECVCHRGICPVIMTDGPANNRAATKII  256 
CAA36475/A     202 LRTQESECACINGSCFTIMTDGPSNGQASYKIF  234 
CAC36997/A     223 LRTQESECVCINGTCTVVMTDGSASGRADTRIL  255 
CAD57252/A     224 LRTQESECVCVNGSCFTIMTDGPSNGAASYKIF  256 
CAO82694/A     235 LRTQESECACVNGSCFTVMTDGPSNGQASYKIF  267 
CAP69845/A     223 MRTQESECVCQDEFCYTLITDGPSDAQAFYKIL  255 

cons           760 :***:* * .        .***.    :  :    792 


AAA43735/B     255 KIREGRII-KEI--FPAGRVEHTEECTCGFASN  284 
AAA43743/B     255 KIREGRII-KEI--FPTGRVEHTEECTCGFASN  284 
AAA43749/B     255 KIREGRII-KEI--LPTGRVEHTEECTCGFASN  284

AAU94779/B     255 KIREGRII-KEI--FPTGRVKHTEECTCGFASN  284

ABL84344/B     255 KIREGRII-KEI--FPTGRVEHTEECTCGFASN  284 
ABO72374/B     255 KIREGRII-KEI--FPTGRVKHTEECTCGFASN  284

ABQ81846/B     255 KIREGRII-KEI--FPTGRVEHTEECTCGFASN  284

ACU12759/B     255 KIREGRII-KEI--FPTGRVKHTEECTCGFASN  284

BAB32609/B     255 KIREGRII-KEI--FPTGRVEHTEECTCGFASN  284     
AAA43093/A     254 YFHKGMII-KEE--SLKGSAKHIEECSCYGHN-  282 
AAA43429/A     255 KAKDGRII-GQT--DVNFNGGHIEECSCYPNE-  283 
AAA43449/A     257 KIEKGKIT-KSI--ELDAPNSHYEECSCYPDT-  285 
AAA43575/A     258 YFKEGKIL-KWE--PLTGTAKHIEECSCYGEQ-  286 
AAD49005/A     256 FIREGKIV-HIS--PLSGSAQHIEECSCYPRY-  284 
AAD49007/A     256 FIREGKIV-HIS--PLSGSAQHVEECSCYPRY-  284 
AAF77036/A     257 KIEKGKVT-KSI--ELNAPNYHYEECSCYPDT-  285 
AAO46232/A     256 FIEEGKIV-HIS--PLSGSAQHVEECSCYPRY-  284 
AAO46822/A     257 KMEKGKVV-KSV--ELDAPNYHYEECSCYPDA-  285 
AAO62036/A     257 WIREGKVI-KYENIPKT-KIQHLEECSCYVDI-  286 
AAO62041/A     257 KIEKGKVV-KSV--ELNAPNYHYEECSCYPDA-  285 
AAO62044/A     256 KIKKGKIV-SVK--DVDAPGFHFEECSCYPSG-  284 
AAO62064/A     257 YFKEGKIQ-KIE--ELTGSAQHIEECSCYGAK-  285 
AAT08005/A     255 KAKQGKII-GQT--EISFNGGHIEECSCYPNE-  283 
AAT37407/A     257 WIREGKVI-KYENIPKT-KIQHLEECSCYVDI-  286 
AAT37414/A     257 WIREGRIIKHEK-VPDD-KIQHLEECSCYTDV-  286 
AAT65406/A     257 YFKEGKIQ-KIE--ELTGSAQHIEECSCYGAE-  285 
AAX78821/A     256 FIKEGKIV-HIS--PLSGSAQHIEECSCYPRY-  284 
AAY52584/A     256 FIREGKIA-HIS--PLSGSAQHVEECSCYPRY-  284 
AAY87411/A     256 FIKEGKIV-HVS--PLSGSAQHIEECSCYPRY-  284 
ABB17695/A     254 KSHEGMVI-NER--EVSFQGGHIEECSCYPNL-  282 
ABB19439/A     257 YFKEGKIQ-KVE--ELTGSAQHIEECSCYGAE-  285 
ABB19610/A     257 KIEKGKVI-KSV--ELNAPNYHYEECSCYPDA-  285 
ABB19747/A     254 KSHKGIVI-NER--EVSFQGGHIEECSCYPNL-  282 
ABB19914/A     256 KIRKGKIV-SMK--DVDATGFHFEECSCYPSG-  284 
ABB20088/A     256 KIRKGKIV-SME--DVDATGFHFEECSCYPSG-  284 
ABB20104/A     255 YFHKGLII-KEE--ALKGSARHIEECSCYGHD-  283 
ABB20243/A     258 YFKEGKIL-KWE--PLTGTAKHIEECSCYGKD-  286 
ABB20375/A     257 YFKEGKIQ-KIE--ELTGSAQHIEECSCYGAK-  285 
ABB20475/A     256 FIKEGKIV-HVS--PLSGSAQHIEECSCYPRY-  284 
ABB20501/A     254 YFHKGTII-KEE--PLKGSAKHIEECSCYGHN-  282 
ABB21754/A     258 YFKEGRIL-KWE--PLTGTAKHIEECSCYGKD-  286 
ABB87198/A     254 KSHKGMVT-NER--EVSFQGGHIEECSCYPNL-  282 
ABB87732/A     256 KIRKGKIV-SVK--DVDATGFHFEECSCYPSG-  284 
ABB87959/A     255 YFHKGLII-KEE--ALQGSARHIEECSCYGHD-  283 
ABB88058/A     257 WIREGRIVKHEN-IPKT-KIQHLEECSCYVDI-  286 
ABB88351/A     257 YFKEGKIQ-KIE--ELTGNAQHIEECSCYGAV-  285 
ABC02269/A     256 FIEEGKIV-HTS--PLSGSAQHVEECSCYPRY-  284 
ABF47958/A     241 KIEKGKVT-KSI--ELNAPNSHYEECSCYPDT-  269 
ABG37189/A     256 FIEEGKIV-HIS--SLSGSAQHVEECSCYPRY-  284 
ABH04381/A     257 WIREGRIV-KYENVPKT-KIQHLEECSCYVDI-  286 
ABI30359/A     256 FIKEGKIV-HIS--PLSGSAQHIEECSCYPRY-  284 
ABI84399/A     257 WIREGKIIKHEK-IPDD-KIQHLEECSCYTDV-  286 
ABI84436/A     254 KSREGMVT-NER--EVSFQGGHIEECSCYPNL-  282 
ABI84475/A     256 KIKKGKIV-GAK--DVDATGFHFEECSCYPSG-  284 
ABI84973/A     257 WIREGKVI-KYENVPKT-KIQHLEECSCYVDT-  286 
ABI84982/A     256 YFHKGLII-KEE--PLRGSARHIEECSCYGHD-  284 
ABI92217/A     257 YFKEGKIQ-KIE--ELTGSAQHIEECSCYGAE-  285 
ABJ09099/A     255 KAKQGKII-GQT--EISFNGGHIEECSCYPNE-  283 
ABK32099/A     256 KIRKGKIM-GVK--DVDATGFHFEECSCYPSG-  284  
ABP49198/A     258 YFKEGKIL-KWE--PLTGTAKHIEECSCYGEQ-  286 
ABP49253/A     258 YFKEGKIL-KWE--PLTGTAKHIEECSCYGEQ-  286 
ABQ97208/A     256 FIEEGKIV-HIS--PLSGSAQHVEECSCYPRY-  284 
ABR28683/A     257 KMEKGRII-KSI--ELDAPNYHYEECSCYPDT-  285 
ABR37723/A     258 YFKEGRIL-KWE--PLTGTAKHIEECSCYGKD-  286 
ABS50124/A     257 KMEKGKII-KSV--ELDAPNYHYEECSCYPDT-  285 
ABS89357/A     256 YFHKGLVI-KEE--PLKGSAKHIEECSCYGHN-  284 
ABU95382/A     257 KIEKGKVV-KSV--ELNAPNYHYEECSCYPDA-  285 
ABV25648/A     257 KMEKGKII-KSV--ELDAPNYHYEECSCYPDT-  285 
ABV29593/A     257 KIEKGKVV-KSV--ELNAPNYHYEECSCYPDS-  285 
ABV82576/A     257 KMEKGRII-KSI--ELDAPNYHYEECSCYPDT-  285 
ABV82587/A     257 KMEKGRII-KSI--ELDAPNYHYEECSCYPDT-  285 
ABW95951/A     237 KMEKGKVV-KSV--ELDAPNYHYEECSCYPDA-  265 
ABX88802/A     256 FIKEGKII-HIS--SLSGSAQHIEECSCYPRY-  284 
ABY84687/A     257 KMEKGKII-KSV--ELDAPNYHYEECSCYPDT-  285 
ACA04655/A     257 YFKEGKIQ-KIE--ELAGNAQHIEECSCYGAV-  285 
ACA04738/A     256 YFHKGLVI-KEE--PLKGSAKHIEECSCYGHN-  284 
ACA04739/A     255 KANQGRII-DQA--DISFNGGHIEECSCYPND-  283 
ACA04740/A     258 YFKEGKTL-KWE--PLTGTAKHIEECSSYGER-  286 
ACA14299/A     256 FFKEGKII-HIS--PLSGSAQHIEECSCYPRY-  284 
ACB70583/A     237 KMEKGKVI-KSV--ELDAPNYHYEECSCYPDA-  265 
ACD47117/A     256 FIREGKIV-HSS--PLSGSAQHVEECSCYPRY-  284 
ACD65204/A     257 KMEKGKII-KSV--ELDAPNYHYEECSCYPDT-  285 
ACD76864/A     256 FIKEGKIV-HIS--PLSGSAQHIEECSCYPRY-  284 
ACD85421/A     255 KAKDGRII-GQT--DVNFNGGHIEECSCYPNE-  283 
ACE73320/A     256 KIRKGKIV-SMK--DVDATGFHFEECSCYPSG-  284 
ACE76594/A     256 FIKEGKII-HIS--PLSGSAQHIEECSCYPRY-  284 
ACE78976/A     256 YFKEGKIQ-KIE--ELTGNAQHIEECSCYGAK-  284 
ACE78981/A     256 YFKEGKIQ-KIE--ELAGNAQHIEECSCYGAV-  284 
ACF20219/A     255 YFHKGLVI-KEE--ALKGSARHVEECSCYGHN-  283 
ACF25054/A     256 FIREGKIV-HIS--PLSGSAQHIEECSCYPRY-  284 
ACF25354/A     255 YFHKGLVI-KEE--ALKGSARHIEECSCYGHN-  283 
ACF33655/A     257 WIREGRII-KYENVPKT-KIQHLEECSCYVDI-  286 
ACF36500/A     256 FIEEGKIV-HTS--TLSGSAQHVEECSCYPRY-  284 
ACF93490/A     255 KAKDGRII-GQT--DISFNGGHIEECSCYPNE-  283 
ACI41114/A     257 WIREGRII-KYENVPKT-KIQHLEECSCYVDI-  286 
ACI48786/A     255 KAKDGRII-GQT--DISFNGGHIEECSCYPNE-  283 
ACN65661/A     256 YFHKGLVV-KEE--ALKGSARHIEECSCYGHS-  284 
ACO24985/A     256 KIEKGKVV-KSV--ELNAPNYHYEECSCYPDA-  284 
ACP50710/A     256 FIREGRIV-HIS--PLSGSAQHVEECSCYPRY-  284 
ACQ73415/A     254 YFHKGMII-KEE--SLKGSAKHIEECSCYGHN-  282 
ACR66907/A     257 WIREGRVI-KYENIPKT-KIQHLEECSCYVDI-  286 
ACR83968/A     257 KIERGKVV-KSV--ELNAPNYHYEECSCYPES-  285 
ACS68311/A     258 YFKEGKII-KWE--PLTGTAKHIEECSCYGEQ-  286 
ACS92897/A     256 FIEEGKIV-HIS--TLSGSAQHVEECSCYPRY-  284 
ACT67761/A     256 FIEEGKIV-HTS--TLSGSAQHVEECSCYPRY-  284 
ACT97062/A     255 KANQGRIV-GQI--DVNFNGGHIEECSCYPND-  283 
ACV41710/A     255 KAKQGKIV-GQI--EISFNGGHIEECSCYPNE-  283 
ADA71169/A     257 KIEKGKVT-KSM--ELNAPNFHYEECSCYPDT-  285 
ADK70163/A     256 KIRKGKIM-SVK--DVDATGFHFEECSCYPSG-  284 
ADU17627/A     258 YFKEGKIL-KWE--PLTGTAKHIEECSCYGVQ-  286 
ADU20276/A     254 KSHKGMVT-NER--EVSFQGGHIEECSCYPNL-  282 
ADU20397/A     257 WIREGRII-KYENVPKT-KIQHLEECSCYVDI-  286 
ADU53220/A     256 KIRKGKIV-SMK--DVDATGFHFEECSCYPSG-  284 
AEB89856/A     258 YFKEGKIL-KWE--SLTGTAKHIEECSCYGER-  286 
AEB89867/A     255 KANQGKII-GQT--DVNFNGGHIEECSCYPND-  283 
AEK50939/A     257 YFKEGKIQ-KIE--ELTGSAQHIEECSCYGAE-  285 
AEK50961/A     255 YFHKGLVI-KEE--ALKGSARHIEECSCYGHN-  283 
BAA06132/A     256 FIEEGKIV-HIS--PLSGSAQHVEECSCYPRY-  284 
BAA06718/A     257 KIEKGKIT-KSI--ELDAPNSHYEECSCYPDA-  285 
BAF03532/A     253 KSQKGLVV-DEK--EISFQGGHIEECSCYPNM-  281 
BAF03569/A     253 KSQKGLVV-NEK--EISFQGGHIEECSCYPNM-  281 
BAF34378/A     256 FIREGKIV-HIS--PLSGSAQHIEECSCYPRY-  284 
BAF34925/A     256 FIREGKIV-HIS--PLSGSAQHIEECSCYPRY-  284 
BAF43417/A     254 KSHEGMVT-NER--EVSFQGGHIEECSCYPNL-  282 
BAF43457/A     255 KANQGKIV-GQT--DVSFNGGHIEECSCYPND-  283 
BAF43461/A     253 KSQKGLVV-NEK--EISFQGGHIEECSCYPNM-  281 
BAF46763/A     257 WIREGKVI-KYENVPKT-KIQHLEECSCYVDT-  286 
BAF46905/A     257 YFKEGKIQ-KIE--ELAGNAQHIEECSCYGAV-  285 
BAF48640/A     256 FVREGKIV-HIS--PLSGSAQHIEECSCYPRY-  284 
BAF48644/A     257 WIREGKVI-KYENVPKT-KIQHLEECSCYVDN-  286 
BAF48646/A     256 YFHKGLVV-KEE--ALKGSARHIEECSCYGHS-  284 
BAF63047/A     257 KIEKGKVT-KSI--ELNAPNFHYEECSCYPDT-  285 
BAG66251/A     255 KAKQGKII-GQT--EISFNGGHIEECSCYPNE-  283 
BAG66259/A     257 WIREGRVM-KYENVPKT-KIQHLEECSCYVDI-  286 
BAG66279/A     255 KANQGKVV-GQT--DISFNGGHIEECSCYPND-  283 
BAH02172/A     256 FIEEGKIV-HIS--PLSGSAQHVEECSCYPRY-  284 
BAH70320/A     256 YFHKGLVV-KEE--ALKGSARHIEECSCYGHS-  284 
BAI39637/A     237 KMEKGKVI-KSV--ELDAPNYHYEECSCYPDA-  265 
BAI48894/A     257 KIEKGKVV-KSV--ELNAPNYHYEECSCYPDA-  285 
BAI50030/A     257 WIREGKVV-KYENIPKT-KIQHLEECSCYVDI-  286 
BAJ07979/A     253 KSQKGLVV-DEK--EISFQGGHIEECSCYPNM-  281 
BAJ10562/A     257 YFREGKIQ-KIE--ELTGNAQHIEECSCYGAA-  285 
CAA36475/A     235 KIEKGKVV-KSS--ELNAPNYHYEECSCYPDA-  263 
CAC36997/A     256 FIEEGKIV-HIS--PLSGSAQHVEECSCYPRY-  284 
CAD57252/A     257 KIEKGKVT-KSI--ELNAPNFHYEECSCYPDT-  285 
CAO82694/A     268 KIERGKVV-KSV--ELNAPNYHYEECSCYPES-  296 
CAP69845/A     256 KIRKGKIM-GVK--DVDATGFHFEECSCYPSG-  284 

cons           793   . *                * ***:.       825 

AAA43735/B     285 KTIECACRDNSYTAKRPFVKLNVET-DTAEIR-  315 
AAA43743/B     285 KTIECACRDNSYTAKRPFVKLNVET-DTAEIR-  315 
AAA43749/B     285 KTIECACRDNSYTAKRPFVKLNVET-DTAEIR-  315

AAU94779/B     285 KTIECACRDNSYTAKRPFVKLNVET-DTAEIR-  315

ABL84344/B     285 KTIECACRDNSYTAKRPFVKLNVET-DTAEIR-  315 
ABO72374/B     285 KTIECACRDNSYTAKRPFVKLNVET-DTAEIR-  315 
ABQ81846/B     285 KTIECACRDNSYTAKRPFVKLNVET-DTAEIR-  315

ACU12759/B     285 KTIECACRDNSYTAKRPFVKLNVET-DTAEIR-  315

BAB32609/B     285 KTIECACRDNSYTAKRPFVKLNVET-DTAEIR-  315 
AAA43093/A     283 QRVTCVCRDNWQGANRPIIEIDMN-KLEHTSR-  313 
AAA43429/A     284 GKVECVCRDNWTGTNRPVLVISP-D-LSYTVG-  313 
AAA43449/A     286 GTVMCVCRDNWHGSNRPWVSFNQ-N-LDYQIG-  315 
AAA43575/A     287 AGVTCTCRDNWQGSNRPVIQIDPV-AMTHTSQ-  317 
AAD49005/A     285 PDVRCVCRDNWKGSNRPVIDINMT-DYSIDSS-  315 
AAD49007/A     285 PEVRCVCRDNWKGSNRPVLYINMV-DYSIDSS-  315 
AAF77036/A     286 GKVMCVCRDNWHGSNRPWVSFDQ-N-LDYQIG-  315 
AAO46232/A     285 PDVRCICRDNWKGSNRPIIDINME-DYSIDSS-  315 
AAO46822/A     286 GEITCVCRDNWHGSNRPWVSFNQ-N-LEYQIG-  315 
AAO62036/A     287 D-VYCVCRDNWKGSNRPWMRIN--N-ETILETG  315 
AAO62041/A     286 GEITCVCRDNWHGSNRPWVSFNQ-N-LEYQIG-  315 
AAO62044/A     285 ENVECVCRDNWRGSNRPWIRFNS-D-LDYQIG-  314 
AAO62064/A     286 SVIKCICRDNWKGANRPVITIDPE-MMTHTSK-  316 
AAT08005/A     284 GKVECVCRDNWTGTNRPVLVISS-D-LSYRVG-  313 
AAT37407/A     287 D-VYCICRDNWKGSNRPWMRIN--N-ETILETG  315 
AAT37414/A     287 D-IYCICRDNWKGSNRPWMRINNET-I-LETG-  315 
AAT65406/A     286 EVIKCICRDNWKGANRPVITINPT-TMTHTSK-  316 
AAX78821/A     285 PDVRCICRDNWKGSNRPVIDINMA-DYSIDSS-  315 
AAY52584/A     285 PEVRCVCRDNWKGSNRPVLYINMA-NYSIDSS-  315 
AAY87411/A     285 PDVRCICRDNWKGSNRPVIDINMA-DYSIDSS-  315 
ABB17695/A     283 GKVECVCRDNWNGMNRPILIFNE-D-LDYEVG-  312 
ABB19439/A     286 EMIKCICRDNWKGANRPVITINPK-TMTHTSK-  316 
ABB19610/A     286 SEVMCVCRDNWHGSNRPWVSFNQ-N-LDYQIG-  315 
ABB19747/A     283 GKVECVCRDNWNGMNRPILIFDE-D-LSYNVG-  312 
ABB19914/A     285 TDVECVCRDNWRGSNRPWIRFNS-D-LDYQIG-  314 
ABB20088/A     285 TDIECVCRDNWRGSNRPWIRFNS-N-LDYQIG-  314 
ABB20104/A     284 SKVTCVCRDNWQGANRPVIEIDMN-AMEHTSQ-  314 
ABB20243/A     287 SEITCTCRDNWQGSNRPVIQIDPT-MMTHTSQ-  317 
ABB20375/A     286 SVIKCICRDNWKGANRPVITIDPE-MMTHTSR-  316 
ABB20475/A     285 PDVRCICRDNWKGSNRPVIDINMA-DYSIDSS-  315 
ABB20501/A     283 QRVTCVCRDNWQGANRPVIEIDMN-NLEHTSR-  313 
ABB21754/A     287 SEITCTCRDNWQGSNRPVIQINPT-TMTHTSQ-  317 
ABB87198/A     283 GKVECVCRDNWNGMNRPILTFDE-D-LNYEVG-  312 
ABB87732/A     285 TDVECVCRDNWRGSNRPWIRFNS-D-LDYQIG-  314 
ABB87959/A     284 SKVTCVCRDNWQGANRPVIEIDMN-AMEHTSQ-  314 
ABB88058/A     287 D-VYCICRDNWKGSNRPWMRIN--N-ETILETG  315 
ABB88351/A     286 GVIKCICRDNWKGANRPVITIDPE-LMTHTSK-  316 
ABC02269/A     285 PGVRCICRDNWKGSNRPVVDINVK-DYSIDSS-  315 
ABF47958/A     270 GKVMCVCRDNWHGSNRPWVSFDQ-N-LDYKIG-  299 
ABG37189/A     285 PGVRCVCRDNWKGSNRPIVDINVK-DYSIVSS-  315 
ABH04381/A     287 D-VYCICRDNWKGSNRPWMRIN--N-ETILETG  315 
ABI30359/A     285 PNVRCVCRDNWKGSNRPVIDINMA-DYSIDSS-  315 
ABI84399/A     287 D-IYCICRDNWKGSNRPWMRINNET-I-LETG-  315 
ABI84436/A     283 GKVECVCRDNWNGMNRPILVFDE-D-LDYEVG-  312 
ABI84475/A     285 ENVECVCRDNWRGSNRPWIRFNS-D-LDYQIG-  314 
ABI84973/A     287 D-VYCVCRDNWKGSNRPWMRIN--N-ETILETG  315 
ABI84982/A     285 QKVSCVCRDNWQGANRPIIEIDMS-TLEHTSR-  315 
ABI92217/A     286 EMIKCICRDNWKGANRPVITINPK-TMTHTSR-  316 
ABJ09099/A     284 GKVECVCRDNWTGTNRPVLIISP-D-LSYRVG-  313 
ABK32099/A     285 ENVECVCRDNWRGSNRPWIRFNS-D-LDYQIG-  314 
ABP49198/A     287 AGITCTCRDNWQGSNRPVIQIDPM-AMTHTSQ-  317 
ABP49253/A     287 ASITCTCRDNWQGSNRPVIQIDPI-AMTHTSQ-  317 
ABQ97208/A     285 PGVRCVCRDNWKGSNRPIVDINVK-DYSIVSS-  315 
ABR28683/A     286 GKVVCVCRDNWHASNRPWVSFDQ-N-LDYQIG-  315 
ABR37723/A     287 SEITCTCRDNWQGSNRPVIQINPT-TMTHTSQ-  317 
ABS50124/A     286 GKVVCVCRDNWHASNRPWVSFDQ-N-LDYQIG-  315 
ABS89357/A     285 QKITCVCRDNWQGANRPIIEIDMT-TLEHTSR-  315 
ABU95382/A     286 GEVICVCRDNWHGSNRPWVSFNQ-N-LEYQIG-  315 
ABV25648/A     286 GKVMCVCRDNWHASNRPWVSFDR-N-LDYQIG-  315 
ABV29593/A     286 GEITCVCRDNWHGSNRPWMSFNQ-N-LEYQIG-  315 
ABV82576/A     286 GKVVCVCRDNWHASNRPWVSFDQ-N-LDYQIG-  315 
ABV82587/A     286 GKVVCVCRDNWHASNRPWVSFDQ-N-LNYQIG-  315 
ABW95951/A     266 GEITCVCRDNWHGSNRPWVSFNQ-N-LEYQIG-  295 
ABX88802/A     285 PDVRCVCRDNWKGSNRPVIDINMA-DYSIDSS-  315 
ABY84687/A     286 GKVVCVCRDNWHASNRPWVSFDQ-N-LDYQIG-  315 
ACA04655/A     286 GVIKCICRDNWKGANRPVITIDPE-MMTHTSK-  316 
ACA04738/A     285 QKITCVCRDNWQGANRPIIEIDMN-TLEHTSR-  315 
ACA04739/A     284 GKVECVCRDNWTGTNRPVLVISP-D-LSYRVG-  313 
ACA04740/A     287 AGITCTCRDNWQGSNRPVIQIDPV-AMTHTSQ-  317 
ACA14299/A     285 PNVRCVCRDNWKGSNRPVIDINMA-DYSIGSS-  315 
ACB70583/A     266 GEITCVCRDNWHGSNRPWVSFNQ-N-LEYQIG-  295 
ACD47117/A     285 PEVRCVCRDNWKGSNRPVLYINVA-DYSIDSS-  315 
ACD65204/A     286 GKVVCVCRDNWHASNRPWVTFDQ-N-LNYQIG-  315 
ACD76864/A     285 PNVRCVCRDNWKGSNRPVIDINMA-DYSIDSS-  315 
ACD85421/A     284 GKVECVCRDNWTGTNRPVLVISP-D-LSYTVG-  313 
ACE73320/A     285 TDIECVCRDNWRGSNRPWIRFNS-D-LDYQIG-  314 
ACE76594/A     285 PDVRCVCRDNWKGSNRPVIDINMA-DYSIDSS-  315 
ACE78976/A     285 EVIKCICRDNWKGANRPVITIDPE-MMTHTSK-  315 
ACE78981/A     285 GVIKCICRDNWKGANRPVITIDPE-MMTHTSK-  315 
ACF20219/A     284 SKVTCVCRDNWQGANRPVIEIDMN-AMEHTSQ-  314 
ACF25054/A     285 PNVRCVCRDNWKGSNRPVIDINMA-DYSTDSS-  315 
ACF25354/A     284 SKVTCVCRDNWQGANRPVIEIDMN-AMEHTSQ-  314 
ACF33655/A     287 D-VYCICRDNWKGSNRPWMRIN--N-ETILETG  315 
ACF36500/A     285 PGVRCVCRDNWKGSNRPIVDINIK-DYSIVSS-  315 
ACF93490/A     284 GKVECICRDNWTGTNRPILVISS-D-LSYTVG-  313 
ACI41114/A     287 D-VYCICRDNWKGSNRPWMRIN--N-ETILETG  315 
ACI48786/A     284 GKVECICRDNWTGTNRPILVISS-D-LSYTVG-  313 
ACN65661/A     285 QKATCVCRDNWQGANRPIIEIDMN-TLEHTSR-  315 
ACO24985/A     285 GEITCVCRDNWHGSNRPWVSFNQ-N-LEYQIG-  314 
ACP50710/A     285 PEVRCVCRDNWKGSNRPVLYINVE-DYNIDSS-  315 
ACQ73415/A     283 QRVTCVCRDNWQGANRPIIEIDMN-NLEHTSR-  313 
ACR66907/A     287 D-VYCICRDNWKGSNRPWMRIN--N-ETILETG  315 
ACR83968/A     286 GEITCVCRDNWHGSNRPWVSFNQ-N-LEYQMG-  315 
ACS68311/A     287 AGITCTCRDNWQGSNRPVIQIDPV-AMTHTSQ-  317 
ACS92897/A     285 PGVRCVCRDNWKGSNRPIVDINVK-DYSIVSS-  315 
ACT67761/A     285 PGVRCVCRDNWKGSNRPIVDINIK-DHSIVSS-  315 
ACT97062/A     284 GKVECVCRDNWTGTNRPVLVISS-D-LSYRVG-  313 
ACV41710/A     284 GKVECVCRDNWTGTNRPVLVISP-D-LSYKVG-  313 
ADA71169/A     286 GTVMCVCRDNWHGSNRPWVSFNQ-N-LDYQIG-  315 
ADK70163/A     285 ENIECVCRDNWRGSNRPWIRFNS-D-LDYQIG-  314 
ADU17627/A     287 AGIICTCRDNWQGSNRPVIQIDPV-AMTHTSQ-  317 
ADU20276/A     283 GKVECVCRDNWNGMNRPILTFDE-D-LNYEVG-  312 
ADU20397/A     287 D-VYCICRDNWKGSNRPWMRIN--N-ETILETG  315 
ADU53220/A     285 TDIECVCRDNWRGSNRPWIRFNS-D-LDYQIG-  314 
AEB89856/A     287 TGITCTCRDNWQGSNRPVIQIDPV-AMTHTSQ-  317 
AEB89867/A     284 GKVECVCRDNWTGTNRPVLVISP-D-LSYRVG-  313 
AEK50939/A     286 EVIKCICRDNWKGANRPVITINPT-TMTHTSK-  316 
AEK50961/A     284 SKVTCVCRDNWQGANRPVIEIDMN-AMEHTSQ-  314 
BAA06132/A     285 PDVRCICRDNWKGSNRPIVDINMK-DHSIDSS-  315 
BAA06718/A     286 GTVMCVCRDNWHGSNRPWVSFNQ-N-LDYQIG-  315 
BAF03532/A     282 GKVECVCRDNWNGMNRPILTFDE-N-LEYEVG-  311 
BAF03569/A     282 GKVECVCRDNWNGMNRPILAFDE-N-LEYEVG-  311 
BAF34378/A     285 PDVRCVCRDNWKGSNRPVIDINMV-DYSIDSS-  315 
BAF34925/A     285 PDVRCVCRDNWKGSNRPVIDINMA-DYSIDSS-  315 
BAF43417/A     283 GKVECVCRDNWNGMNRPILIFDE-D-LDYEVG-  312 
BAF43457/A     284 GKVECVCRDNWTGTNRPVLIISP-D-LSYRVG-  313 
BAF43461/A     282 GKVECVCRDNWNGMNRPILAFDE-N-LEYEVG-  311 
BAF46763/A     287 D-VYCVCRDNWKGSNRPWMRIN--N-ETILETG  315 
BAF46905/A     286 GVIKCICRDNWKGANRPVITIDPE-MMTHTSK-  316 
BAF48640/A     285 PNVRCVCRDNWKGSNRPVIDISMA-DYSIDSS-  315 
BAF48644/A     287 D-VYCICRDNWKGSNRPWMRIN--N-ETIIETG  315 
BAF48646/A     285 QKVTCVCRDNWQGANRPIIEINMN-TLEHTSR-  315 
BAF63047/A     286 GTVMCVCRDNWHGSNRPWVSFNQ-N-LDYQIG-  315 
BAG66251/A     284 GKVECVCRDNWTGTNRPVLVISS-D-LSYRVG-  313 
BAG66259/A     287 D-IYCICRDNWKGSNRPWMRIN-NETILETG--  315 
BAG66279/A     284 GKVECVCRDNWTGTNRPVLVISP-D-LSYRVG-  313 
BAH02172/A     285 PDVRCICRDNWKGSNRPIVDINMK-DYSIDSS-  315 
BAH70320/A     285 QKVTCVCRDNWQGANRPIIEIDMN-TLEHTSR-  315 
BAI39637/A     266 GEITCVCRDNWHGSNRPWVSFNQ-N-LEYQIG-  295 
BAI48894/A     286 GKIMCVCRDNWHGSNRPWVSFNQ-N-LEYQIG-  315 
BAI50030/A     287 D-VYCICRDNWKGSNRPWMRIN--N-ETILETG  315 
BAJ07979/A     282 GKVECVCRDNWNGMNRPILTFNE-N-LEYEVG-  311 
BAJ10562/A     286 GVIKCVCRDNWKGANRPVITIDPE-MMTHTSK-  316 
CAA36475/A     264 GEVMCVCRDNWHGSNRPWVSFNK-N-LDYQIG-  293 
CAC36997/A     285 PDVRCICRDNWKGSNRPIVDINMK-DYSIDSS-  315 
CAD57252/A      286 GTVMCVCRDNWHGSNRPWVSFNQ-N-LDYQIG-  315 
CAO82694/A     297 GEITCVCRDNWHGSNRPWVSFNQ-N-LEYQIG-  326 
CAP69845/A     285 ENVECVCRDNWRGSNRPWIRFNS-D-LDYQIG-  314 

cons           826     * ****    :** : :.             858 

AAA43735/B     316 LMCTETYLDTPRPDDG-SITGPCESN-G-DK-G  344 
AAA43743/B     316 LMCTETYLDTPRPDDG-SITGPCESN-G-DK-G  344 
AAA43749/B     316 LMCTKTYLDTPRPDDG-SIAGPCESN-G-DK-W  344

AAU94779/B     316 LMCTETYLDTPRPDDG-SITGPCESN-G-DK-G  344

ABL84344/B     316 LMCTETYLDTPRPDDG-SITGPCESN-G-DK-G  344 
ABO72374/B     316 LMCTETYLDTPRPDDG-SITGPCESN-G-NK-G  344

ABQ81846/B     316 LMCTETYLDTPRPDDG-SITGPCESN-G-DK-G  344

ACU12759/B     316 LMCTETYLDTPRPDDG-SITGPCESN-G-DK-G  344 
BAB32609/B     316 LMCTETYLDTPRPDDG-SITGPCESN-G-EK-G  344 
AAA43093/A     314 YICTGVLTDTSRPKD-KTIG-ECFNPIT-GS-P  342 
AAA43429/A     314 YLCAGIPTDTPRGEDS-QFTGSCTKPLG-NQ--  342 
AAA43449/A     316 YICSGVFGDNPRPKDG-K--GRC-DPVN-VD-G  342 
AAA43575/A     318 YICSPVLTDNPRPND-PTVG-KCNDPYP-GN-N  346 
AAD49005/A     316 YVCSGLVGDTPRYDDS-SSSSNCRDPNN-ER-G  345 
AAD49007/A     316 YVCSGLVGDTPRNDDS-SSSSNCRDPNN-ER-G  345 
AAF77036/A     316 YICSGVFGDNPRPNDG-T--GSC-GPVS-SN-G  342 
AAO46232/A     316 YVCSGLVGDTPRNDDR-SSNSNCRNPNN-ER-G  345 
AAO46822/A     316 YICSGVFGDNPRPNDG-T--GSC-GPVS-PN-G  342 
AAO62036/A     316 YVCSKFHSDTPRPADP-STV-SCDSPSN-VN-G  344 
AAO62041/A     316 YICSGVFGDNPRPNDG-T--GSC-GPVS-SN-G  342 
AAO62044/A     315 YVCSGVFGDNPRPMDS-T--GSCNSPINNGK-G  343 
AAO62064/A     317 YLCSKILTDTSRPND-PIDG-DCDAPIT-GGSP  346 
AAT08005/A     314 YLCAGLPSDTPRGEDS-QFTGSCTSPMG-NQ--  342 
AAT37407/A     316 YVCSKFHSDTPRPADP-SKV-SCDSPSN-VN-G  344 
AAT37414/A     316 YICSKFHSDTPRPSDP-STV-SCSSPSG-ID-G  344 
AAT65406/A     317 YLCSKILTDTSRPND-PGSG-NCDAPIT-GGSP  346 
AAX78821/A     316 YVCSGLVGDTPRNDDS-SSSSNCRDPNN-ER-G  345 
AAY52584/A     316 YVCSGLVGDTPRNDDR-SSSSNCRDPNN-ER-G  345 
AAY87411/A     316 YVCSGLVGDTPRNDDS-SSSSNCRDPNN-ER-G  345 
ABB17695/A     313 YLCAGIPTDTPRVQDS-SFTGSCTSAVG-GS--  341 
ABB19439/A     317 YLCSRILTDTSRPND-PGSG-NCDAPIT-GGSP  346 
ABB19610/A     316 YICSGVFGDNPRPNDG-T--GSC-GPVS-SN-G  342 
ABB19747/A     313 YLCAGIPTDTPRIQDS-SFTGSCTNAVG-GS--  341 
ABB19914/A     315 YVCSGIFGDNPRPVDG-T--GSCNSPVNNGK-G  343 
ABB20088/A     315 YVCSGIFGDNPRPMDG-T--GSCNSPVNNGK-G  343 
ABB20104/A     315 YLCTGVLTDTSRPSD-KSIG-DCNNPIT-GS-P  343 
ABB20243/A     318 YICSPVLTDNPRPND-PTVG-KCNDPYP-GN-N  346 
ABB20375/A     317 YLCSKILTDTSRPND-PING-DCDAPIT-GGSP  346 
ABB20475/A     316 YVCSGLVGDTPRNDDS-SSSSNCRDPNN-ER-G  345 
ABB20501/A     314 YICTGVLTDTSRPKD-KAIG-ECFNPIT-GS-P  342 
ABB21754/A     318 YICSPVLTDNPRPND-PTVG-KCNDPYP-GN-N  346 
ABB87198/A     313 YLCAGIPTDTPRVQDS-SFTGSCTNAVG-GS--  341 
ABB87732/A     315 YVCSGIFGDNPRPVDG-T--GSCTSPVNNGK-G  343 
ABB87959/A     315 YLCTGVLTDTSRPSD-KSIG-DCNNPIT-GS-P  343 
ABB88058/A     316 YVCSKFHSDTPRPADP-STI-SCDSPSN-VN-G  344 
ABB88351/A     317 YLCSKVLTDTSRPND-PTNG-NCDAPIT-GGSP  346 
ABC02269/A     316 YVCSGLVGDTPRNNDR-SSSSYCRNPNN-EK-G  345 
ABF47958/A     300 YICSGVFGDNPRPKDG-T--GSC-GPVS-AD-G  326 
ABG37189/A     316 YVCSGLVGDTPRKNDS-SSSSHCLNPNN-EE-G  345 
ABH04381/A     316 YVCSKFHSDTPRPADP-STV-SCDSPSN-IN-G  344 
ABI30359/A     316 YVCSGLVGDTPRNDDS-SSSSNCRDPNN-ER-G  345 
ABI84399/A     316 YICSKFHSDTPRPSDP-STI-SCNSPSG-ID-G  344 
ABI84436/A     313 YLCAGIPTDTPRVQDS-SFTGSCTNAVG-GS--  341 
ABI84475/A     315 YVCSGVFGDNPRPVDG-T--GSCNSPVNNGK-G  343 
ABI84973/A     316 YVCSKFHSDTPRPADP-STV-SCDSPSN-VN-G  344 
ABI84982/A     316 YVCTGVLTDTSRPGDKPN-G-DCSNPIT-GS-P  344 
ABI92217/A     317 YLCSKILTDTSRPND-PGSG-NCDEPIT-GGSP  346 
ABJ09099/A     314 YLCAGLPSDTPRGEDS-QFTGSCTSPMG-NQ--  342 
ABK32099/A     315 YVCSGVFGDNPRPVDG-T--GSCSGPINNGK-G  343 
ABP49198/A     318 YICSPVLTDNPRPND-PTVG-KCNDPYP-GN-N  346 
ABP49253/A     318 YICSPVLTDNPRPND-PAVG-KCNDPYP-GN-N  346 
ABQ97208/A     316 YVCSGLVGDTPRKSDR-SSSSYCRNPNN-EK-G  345 
ABR28683/A     316 YICSGVFGDNPRSNDG-K--GNC-GPVL-SN-G  342 
ABR37723/A     318 YICSPVLTDNPRPND-PTVG-KCNDPYP-GN-N  346 
ABS50124/A     316 YICSGVFGDNPRSNDG-K--GNC-GPVL-SN-G  342 
ABS89357/A     316 YVCTGVLTDTSRPGDKPS-G-DCSNPIT-GS-P  344 
ABU95382/A     316 YICSGVFGDNPRPNDG-T--GSC-GPVS-SN-G  342 
ABV25648/A     316 YICSGVFGDNPRSNDG-K--GNC-GPVL-SN-G  342 
ABV29593/A     316 YICSGIFGDNPRPKDG-T--GSC-SPVS-SN-G  342 
ABV82576/A     316 YICSGVFGDNPRSNDG-K--GNC-GPVL-SN-G  342 
ABV82587/A     316 YICSGVFGDNPRSNDG-K--GNC-GPVL-SN-G  342 
ABW95951/A     296 YICSGVFGDNPRPNDG-T--GSC-GPMS-PN-G  322 
ABX88802/A     316 YVCSGLVGDTPRNDDS-SSSSNCRDPNN-ER-G  345 
ABY84687/A     316 YICSGVFGDNPRSNDG-K--GNC-GPVL-SN-G  342 
ACA04655/A     317 YLCSKILTDTSRPND-PTNG-NCDAPIT-GGSP  346 
ACA04738/A     316 YVCTGILTDTSRPGDKPS-G-DCSNPIT-GS-P  344 
ACA04739/A     314 YLCAGIPSDTPRGEDA-QFTGSCTSPMG-NQ--  342 
ACA04740/A     318 YICSPVLTDNPRPND-PTVG-KCNDPYP-GN-N  346 
ACA14299/A     316 YVCSGLVGDTPRNDDS-SSSSNCKDPNN-ER-G  345 
ACB70583/A     296 YICSGVFGDNPRPNDG-K--GSC-GPVS-SN-G  322 
ACD47117/A     316 YLCSGLVGDTPRNEDS-SSSSNCRDPNN-ER-G  345 
ACD65204/A     316 YICSGVFGDNPRSNDG-K--GNC-GPVP-SN-G  342 
ACD76864/A     316 YVCSGLVGDTPRNDDS-SSSSNCRDPNN-ER-G  345 
ACD85421/A     314 YLCAGIPTDTPRGEDS-QFTGSCTSPLG-SQ--  342 
ACE73320/A     315 YVCSGIFGDNPRPVDG-T--GSCNSPVNNGK-G  343 
ACE76594/A     316 YVCSGLVGDTPRNDDS-SSSSNCRDPNN-ER-G  345 
ACE78976/A     316 YLCSKILTDISRPND-PTSG-NCDAPVT-GGGP  345 
ACE78981/A     316 YLCSKILTDTSRPND-PTNG-NCDAPIT-GGSP  345 
ACF20219/A     315 YLCTGVLTDTSRPSD-KSIG-DCNNPIT-GS-P  343 
ACF25054/A     316 YVCSGLVGDTPRNDDS-SSSSNCRDPNN-ER-G  345 
ACF25354/A     315 YLCTGVLTDTSRPSD-KSTG-DCSNPIT-GS-P  343 
ACF33655/A     316 YVCSKFHSDTPRPADP-STM-SCDSPSN-IN-G  344 
ACF36500/A     316 YVCSGLVGDTPRKNDS-SSSSHCLDPNN-EE-G  345 
ACF93490/A     314 YLCAGIPTDTPRGEDS-QFTGSCTSPLG-NK--  342 
ACI41114/A     316 YVCSKFHSDTPRPADP-STM-SCDSPSN-VN-G  344 
ACI48786/A     314 YLCAGIPTDTPRGEDS-QFTGSCTSPLG-NK--  342 
ACN65661/A     316 YVCTGILTDTSRPGD-KSSG-DCSNPIT-GS-P  344 
ACO24985/A     315 YICSGIFGDNPRPNDG-T--GSC-GPVS-SN-G  341 
ACP50710/A     316 YVCSGLVGDTPRSDDS-SSSSNCRDPNN-ER-G  345 
ACQ73415/A     314 YICTGVLTDTSRPKD-KTIG-ECFNPIT-GS-P  342 
ACR66907/A     316 YVCSKFHSDTPRPADP-STI-SCDSPSN-VN-G  344 
ACR83968/A     316 YICSGIFGDNPRPNDK-T--GSC-GPVF-SN-G  342 
ACS68311/A     318 YICSPVLTDNPRPND-PAVG-KCNDPYP-GN-N  346 
ACS92897/A     316 YVCSGLVGDTPRKNDS-FSSSHCLDPNN-EE-G  345 
ACT67761/A     316 YVCSGLVGDTPRKNDS-SSSSHCLDPNN-EE-G  345 
ACT97062/A     314 YLCAGLPSDTPRGEDA-QFTGSCTSPMG-NQ--  342 
ACV41710/A     314 YLCAGLPSDTPRGEDS-QFTGSCTSPMG-NQ--  342 
ADA71169/A     316 YICSGVFGDNPRPKDG-E--GSC-NPVT-VD-G  342 
ADK70163/A     315 YVCSGVFGDNPRPVDG-T--GSCSGPINNGK-G  343 
ADU17627/A     318 YICSPVLTDNPRPND-PAVG-KCNDPYP-GN-N  346 
ADU20276/A     313 YLCAGIPTDTPRVQDS-SFSGSCTNAVG-GS--  341 
ADU20397/A     316 YVCSKFHSDTPRPADP-STM-SCDSPSN-VN-G  344 
ADU53220/A     315 YVCSGIFGDNPRPVDG-T--GSCNSPVNNGK-G  343 
AEB89856/A     318 YICSPVLTDNPRPND-PNIG-KCNDPYP-GN-N  346 
AEB89867/A     314 YLCAGLPSDTPRGEDA-QFTGSCTSPMG-NQ--  342 
AEK50939/A     317 YLCSKILTDTSRPND-PGSG-NCDAPIT-GGSP  346 
AEK50961/A     315 YLCTGVLTDTSRPSD-KSMG-DCNNPIT-GS-P  343 
BAA06132/A     316 YVCSGLVGDTPRNDDR-SSKSNCRNPNN-ER-G  345 
BAA06718/A     316 YICSGVFGDNPRPKDG-K--GSC-DPVT-VD-G  342 
BAF03532/A     312 YLCAGIPTDTPRVQDS-SFTGSCTNAVG-GS--  340 
BAF03569/A     312 YLCAGIPTDTPRVQDS-SFTGSCTNAVG-GS--  340 
BAF34378/A     316 YVCSGLVGDTPRNDDS-SSSSNCRDPNN-ER-G  345 
BAF34925/A     316 YVCSGLVGDTPRNDDS-SSSSNCRDPNN-ER-G  345 
BAF43417/A     313 YLCAGIPTDTPRVQDS-SFTGSCTNAVG-GS--  341 
BAF43457/A     314 YLCAGLPSDTPRGEDA-QFTGSCTSPMG-NQ--  342 
BAF43461/A     312 YLCAGIPTDTPRVQDS-SFTGSCTNAVG-GS--  340 
BAF46763/A     316 YVCSKFHSDTPRPADP-STV-SCDSPSN-VN-G  344 
BAF46905/A     317 YLCSKILTDTSRPND-PTNG-NCDAPIT-GGSP  346 
BAF48640/A     316 YVCSGLVGDTPRNDDS-SSSSNCKDPNN-ER-G  345 
BAF48644/A     316 YVCSKFHSDTPRPVDP-STV-SCDSPSN-VN-G  344 
BAF48646/A     316 YVCTGILTDTSRPGD-KSSG-DCSNPIT-GS-P  344 
BAF63047/A     316 YICSGVFGDNPRPKDG-E--GSC-NPVT-VD-G  342 
BAG66251/A     314 YLCAGLPSDTPRGEDN-QFTGSCTSPMG-NQ--  342 
BAG66259/A     316 YVCSKFHSDTPRPADPSTIS--CDSP---SN-V  342 
BAG66279/A     314 YLCAGLPSDTPRGEDA-QFTGSCTSPMG-NQ--  342 
BAH02172/A     316 YVCSGLVGDTPRNDDR-SSKSNCRNPNN-ER-G  345 
BAH70320/A     316 YVCTGILTDTSRPGD-KSSG-DCSNPIT-GS-P  344 
BAI39637/A     296 YICSGVFGDNPRPNDG-T--GSC-GPVS-SN-G  322 
BAI48894/A     316 YICSGVFGDNPRPNDG-T--GSC-GPVS-SN-G  342 
BAI50030/A     316 YVCSKFHSDTPRPADP-STV-SCDSPSN-IN-G  344 
BAJ07979/A     312 YLCAGIPTDTPRVQDS-SFTGSCTNAVG-GS--  340 
BAJ10562/A     317 YLCSKILTDTSRPND-PTNG-NCDAPIT-GGSP  346 
CAA36475/A     294 YICSGVFGDNPRPNDG-T--GSC-GPVS-SN-G  320 
CAC36997/A     316 YVCSGLVGDTPRNDDR-SSKSNCRNPNN-ER-G  345 
CAD57252/A     316 YICSGVFGDNPRPKDG-E--GSC-NPVT-VD-G  342 
CAO82694/A     327 YICSGIFGDNPRPNDR-T--GSC-GPVF-SN-G  353 
CAP69845/A     315 YVCSGVFGDNPRPVDG-T--GSCSGPINNGK-G  343 

cons           859  :*:    * .*  *       *            891 

AAA43735/B     345 L--G-G--IKGGFVHQRMASKIGRWYSRTMSKT  372 
AAA43743/B     345 L--G-G--IKGGFVHQRMASKIGRWYSRTMSKT  372 
AAA43749/B     345 L--G-G--IKGGFVHQRMASKIGRWYSRTMSKT  372

AAU94779/B     345 S--G-G--IKGGFVHQRMASKIGRWYSRTMSKT  372

ABL84344/B     345 S--G-G--IKGGFVHQRMASKIGRWYSRTMSKT  372 
ABO72374/B     345 S--G-G--IKGGFVHQRMASKIGRWYSRTMSKT  372 
ABQ81846/B     345 L--G-G--IKGGFVHQRMASKIGRWYSRTMSKT  372

ACU12759/B     345 S--G-G--IKGGFVHQRMASKIGRWYSRTMSKT  372

BAB32609/B     345 R--G-G--IKGGFVHQRMASKIGRWYSRTMSKT  372 
AAA43093/A     343 G--APG--IKGFGFLN----EDNTWLGRTISPR  367 
AAA43429/A     343 G---YG--IKGFGFRQ----GNDVWAGRTISRT  366 
AAA43449/A     343 A---DG--VKGFSYRY----GNGGWIGRTKSNS  366 
AAA43575/A     347 N--N-G--VKGFSYLD----GGNTWLGRTISIA  370 
AAD49005/A     346 N--P-G--VKGWAFDN----GNDVWMGRTISKD  369 
AAD49007/A     346 A--P-G--VKGWAFDN----GNDIWMGRTIKED  369 
AAF77036/A     343 A---NG--IKGFSFRY----DNGVWIGRTKSTS  366 
AAO46232/A     346 N--P-G--VKGWAFDN----GDDVWMGRTISKD  369 
AAO46822/A     343 A---YG--VKGFSFKY----GNGVWIGRTKSTN  366 
AAO62036/A     345 G--P-G--VKGFGFKS----GNDVWLGRTVSTS  368 
AAO62041/A     343 A---YG--VKGFSFKY----GNGVWIGRTKSTS  366 
AAO62044/A     344 R---YG--VKGFSFRY----GDGVWIGRTKSLE  367 
AAO62064/A     347 D--P-G--VKGFAFLD----GDNSWLGRTISKD  370 
AAT08005/A     343 G---YG--VKGFGFRQ----GNDVWMGRTISRT  366 
AAT37407/A     345 G--P-G--VKGFGFKT----GNDVWLGRTVSTS  368 
AAT37414/A     345 R--R-G--VKGFGFKV----QNDVWLGRTISYS  368 
AAT65406/A     347 D--P-G--VKGFAFLD----GGNSWLGRTISKD  370 
AAX78821/A     346 N--P-G--VKGWAFDN----GDDVWMGRTISKD  369 
AAY52584/A     346 A--P-G--VKGWAFDN----GNDIWMGRTIKKD  369 
AAY87411/A     346 N--P-G--VKGWAFDD----GSDVWMGRTISKD  369 
ABB17695/A     342 GTNNYG--VKGFGFRQ----GNSVWAGRTVSIS  368 
ABB19439/A     347 D--P-G--VKGFAFLD----GGNSWLGRTISKD  370 
ABB19610/A     343 A---YG--VKGFSFKY----GNGVWIGRTKSTS  366 
ABB19747/A     342 GTNNYG--VKGFGFRQ----GNSVWAGRTVSIS  368 
ABB19914/A     344 R---YG--VKGFSFRY----GDGVWIGRTKSLE  367 
ABB20088/A     344 R---YG--VKGFSFRY----GDGVWIGRTKSLE  367 
ABB20104/A     344 G--APG--VKGFGFLD----SSNTWLGRTISPR  368 
ABB20243/A     347 N--N-G--VKGFSYLD----GDNTWLGRTISTA  370 
ABB20375/A     347 D--P-G--VKGFAFLD----GDNSWLGRTISKD  370 
ABB20475/A     346 N--P-G--VKGWAFDD----GDDVWMGRTISKD  369 
ABB20501/A     343 G--APG--IKGFGFLN----ENNTWLGRTISPK  367 
ABB21754/A     347 N--N-G--VKGFSYLD----GDNTWLGRTISTA  370 
ABB87198/A     342 GTNNYG--VKGFGFRQ----GNSVWAGRTVSIS  368 
ABB87732/A     344 R---YG--VKGFSFRY----GDGVWIGRTKSLE  367 
ABB87959/A     344 G--APG--VKGFGFLD----SSNTWLGRTISPR  368 
ABB88058/A     345 G--P-G--VKGFGFKV----GNDVWLGRTVSTS  368 
ABB88351/A     347 D--P-G--VKGFAFLD----GENSWLGRTISKD  370 
ABC02269/A     346 T--H-G--VKGWAFDD----GNDVWMGRTISED  369 
ABF47958/A     327 A---NG--VKGFSYKY----GNGVWIGRTKSDS  350 
ABG37189/A     346 G--H-G--VKGWAFDD----GNDVWMGRTISEK  369 
ABH04381/A     345 G--P-G--VKGFGFKA----GNDVWLGRTVSTS  368 
ABI30359/A     346 N--P-G--VKGWAFDD----GNDVWMGRTISKD  369 
ABI84399/A     345 R--R-G--VKGFGFKV----QNDVWLGRTISYS  368 
ABI84436/A     342 GTNNYG--VKGFGFRQ----GNSVWAGRTVSIS  368 
ABI84475/A     344 R---YG--VKGFSFRY----GDGVWIGRTKSLE  367 
ABI84973/A     345 G--P-G--VKGFGFKT----GDDVWLGRTVSTN  368 
ABI84982/A     345 G--APG--VKGFGFLN----GDNTWLGRTISPR  369 
ABI92217/A     347 D--P-G--VKGFAFLD----GGNSWLGRTISKD  370 
ABJ09099/A     343 G---YG--VKGFGFRQ----GNDVWMGRTISRT  366 
ABK32099/A     344 R---YG--VKGFSFRY----GDGVWIGRTKSLE  367 
ABP49198/A     347 N--N-G--VKGFSYLD----GDNTWLGRTISTA  370 
ABP49253/A     347 N--N-G--VKGFSYLD----GGNTWLGRTISTA  370 
ABQ97208/A     346 N--H-G--VKGWAFDD----GDDVWMGRTIGEE  369 
ABR28683/A     343 A---NG--VKGFSFRY----GNGVWIGRTKSIS  366 
ABR37723/A     347 N--N-G--VKGFSYLD----GDNTWLGRTISTA  370 
ABS50124/A     343 A---NG--VKGFSFRY----GNGVWIGRTKSIS  366 
ABS89357/A     345 G--APG--VKGFGFLN----GDNTWLGRTISPR  369 
ABU95382/A     343 A---YG--VKGFSFRY----GKGVWIGRTKSTS  366 
ABV25648/A     343 A---NG--VKGFSFRY----GNGVWIGRTKSIS  366 
ABV29593/A     343 A---NG--VKGFSFKY----GNGVWIGRTKSTS  366 
ABV82576/A     343 A---NG--VKGFSFRY----GNGVWIGRTKSIS  366 
ABV82587/A     343 A---NG--VKGFSFRY----GNGVWIGRTKSIS  366 
ABW95951/A     323 A---YG--VKGFSFKY----GNGVWIGRTKSTN  346 
ABX88802/A     346 N--P-G--VKGWAFDN----GKDVWMGRTISKD  369 
ABY84687/A     343 A---NG--VKGFSFRY----GNGVWIGRTKSIS  366 
ACA04655/A     347 D--P-G--VKGFAFLD----GENSWLGRTISKD  370 
ACA04738/A     345 S--APG--VKGFGFLN----GDNTWLGRTFSPR  369 
ACA04739/A     343 G---YG--VKGFGFRQ----GTDVWMGRTISRT  366 
ACA04740/A     347 N--N-G--VKGFSYLD----GSNTWLGRTISTA  370 
ACA14299/A     346 N--P-G--VKGWAFDN----ENDVWMGRTISKD  369 
ACB70583/A     323 A---YG--VKGFSFKY----GNGVWIGRTKSTN  346 
ACD47117/A     346 G--P-G--VKGWAFDN----GNDVWMGRTIKQD  369 
ACD65204/A     343 A---NG--VKGFSFRY----GNGVWIGRTKSIS  366 
ACD76864/A     346 N--P-G--VKGWAFDN----GDDVWMGRTISKD  369 
ACD85421/A     343 G---YG--VKGFGFRQ----GNDVWAGRTISRT  366 
ACE73320/A     344 R---YG--VKGFSFRY----GDGVWIGRTKSLE  367 
ACE76594/A     346 N--P-G--VKGWAFDN----GNDVWMGRTISKD  369 
ACE78976/A     346 D--P-G--VKGFAFLD----GENSWLGRTISKD  369 
ACE78981/A     346 D--P-G--VKGFAFLD----GENSWLGRTISKD  369 
ACF20219/A     344 G--APG--VKGFGFLD----SGNTWLGRTISPH  368 
ACF25054/A     346 N--P-G--VKGWAFDN----GNDVWMGRTISKD  369 
ACF25354/A     344 G--APG--VKGFGFLD----SGNTWLGRTISPR  368 
ACF33655/A     345 G--P-G--VKGFGFKA----GNDVWLGRTVSTS  368 
ACF36500/A     346 G--H-G--VKGWAFDD----GNDVWMGRTISEK  369 
ACF93490/A     343 G---YG--VKGFGFRQ----GTDVWAGRTISRT  366 
ACI41114/A     345 G--P-G--VKGFGFKA----GNDVWLGRTVSTS  368 
ACI48786/A     343 G---YG--VKGFGFRQ----GTDVWAGRTISRT  366 
ACN65661/A     345 G--APG--VKGFGFLN----GDNTWLGRTISPR  369 
ACO24985/A     342 A---YG--VKGFSFKY----DNGVWIGRTKSTS  365 
ACP50710/A     346 G--P-G--VKGWAFDN----GNDVWMGRTIKKD  369 
ACQ73415/A     343 G--APG--IKGFGFLN----EDNTWLGRTISPK  367 
ACR66907/A     345 G--P-G--VKGFGFKT----GNDVWLGRTVSTS  368 
ACR83968/A     343 A---NG--VKGFSFKY----GNGVWIGRTKSTS  366 
ACS68311/A     347 N--N-G--VKGFSYLD----GGNTWLGRTISTA  370 
ACS92897/A     346 G--H-G--VKGWAFDD----GNDVWMGRTISEK  369 
ACT67761/A     346 G--H-G--VKGWAFDD----GNDVWMGRTISEK  369 
ACT97062/A     343 G---YG--VKGFGFRQ----GTDVWMGRTISRT  366 
ACV41710/A     343 G---YG--VKGFGFRQ----GNDVWMGRTISRT  366 
ADA71169/A     343 A---NG--VKGFSYKY----GNGVWIGRTKSNR  366 
ADK70163/A     344 R---YG--VKGFSFRY----GDGVWIGRTKSLE  367 
ADU17627/A     347 N--N-G--VKGFSYLD----GGNTWLGRTISTA  370 
ADU20276/A     342 GTNNYG--VKGFGFRQ----GNSVWAGRTVSIS  368 
ADU20397/A     345 G--P-G--VKGFGFKA----GNDVWLGRTVSTS  368 
ADU53220/A     344 R---YG--VKGFSFRY----GDGVWIGRTKSLE  367 
AEB89856/A     347 N--N-G--VKGFSYLD----GVNTWLGRTISTA  370 
AEB89867/A     343 G---YG--VKGFGFRQ----GTDVWMGRTISRT  366 
AEK50939/A     347 D--P-G--VKGFAFLD----GGNSWLGRTISKD  370 
AEK50961/A     344 G--APG--VKGFGFLD----SSNTWLGRTISPR  368 
BAA06132/A     346 N--H-G--VKGWAFDN----GDDVWMGRTISKD  369 
BAA06718/A     343 A---DG--VKGFSYRY----GNGVWIGRTKSNS  366 
BAF03532/A     341 GTNNYG--VKGFGFRQ----GNSVWAGRTISTS  367 
BAF03569/A     341 GTNNYG--VKGFGFRQ----GNSVWAGRTISTS  367 
BAF34378/A     346 N--P-G--VKGWAFDN----GNDVWMGRTISKD  369 
BAF34925/A     346 N--P-G--VKGWAFDN----GNDVWMGRTISKD  369 
BAF43417/A     342 GTNNYG--VKGFGFRQ----GNSVWAGRTVSIS  368 
BAF43457/A     343 G---YG--VKGFGFRQ----GTDVWMGRTISRT  366 
BAF43461/A     341 GTNNYG--VKGFGFRQ----GNSVWAGRTISTS  367 
BAF46763/A     345 G--P-G--VKGFGFKA----GNDVWLGRTVSTS  368 
BAF46905/A     347 D--P-G--VKGFAFLD----GENSWLGRTISKD  370 
BAF48640/A     346 N--P-G--VKGWAFDN----GNDVWMGRTISKD  369 
BAF48644/A     345 G--P-G--VKGFGFKA----GNDVWLGRTVSTS  368 
BAF48646/A     345 G--APG--VKGFGFLN----GDNTWLGRTISPR  369 
BAF63047/A     343 A---DG--VKGFSYKY----GNGVWIGRTKSNR  366 
BAG66251/A     343 G---YG--VKGFGFRQ----GNDVWMGRTISRT  366 
BAG66259/A     343 N--G-GPGVKGFGFKT----GNDVWLGRTVSTS  368 
BAG66279/A     343 G---YG--VKGFGFRQ----GTDVWMGQTISRT  366 
BAH02172/A     346 N--H-G--VKGWAFDN----GDDVWMGRTISKD  369 
BAH70320/A     345 G--APG--VKGFGFLN----GDNTWLGRTISPR  369 
BAI39637/A     323 A---DG--VKGFSFKY----GNGVWIGRTKSPN  346 
BAI48894/A     343 A---YG--VKGFSFKY----GNGVWIGRTKSTS  366 
BAI50030/A     345 G--P-G--VKGFGFKT----GDDVWLGRTVSTS  368 
BAJ07979/A     341 GTNNYG--VKGFGFRQ----GTSVWAGRTISIS  367 
BAJ10562/A     347 D--P-G--VKGFAFLD----RENSWLGRTISKD  370 
CAA36475/A     321 A---YG--IKGFSFKY----GNGVWIGRTKSTS  344 
CAC36997/A     346 N--H-G--VKGWAFDN----GNDVWMGRTISKD  369 
CAD57252/A      343 A---DG--VKGFSYKY----GNGVWIGRTKSNR  366 
CAO82694/A     354 A---NG--VKGFSFRY----GNGVWIGRTKSTS  377 
CAP69845/A     344 R---YG--VKGFSFRY----GDGVWIGRTKSLE  367 

cons           892      *  :**           . * .:*      924 


AAA43735/B     373 ERMGMELYVK-------YDGD-PWTDSDA----  393 
AAA43743/B     373 ERMGMELYVK-------YDGD-PWTDSEA----  393 
AAA43749/B     373 NRMGMELYVK-------YDGD-PWTDSDA----  393

AAU94779/B     373 KRMGMGLYVK-------YDGD-PWTDSDA----  393

ABL84344/B     373 KRMGMGLYVK-------YDGD-PWIDSDA----  393 
ABO72374/B     373 KRMGMGLYVK-------YDGD-PWSDSDA----  393

ABQ81846/B     373 ERMGMELYVK-------YDGD-PWTDSDA----  393  
ACU12759/B     373 ERMGMGLYVK-------YDGD-PWADSDA----  393

BAB32609/B     373 ERMGMELYVK-------YDGD-PWTDSDA----  393 
AAA43093/A     368 LRSGFEMLKI-------PNAG-TDPE-SK----  387 
AAA43429/A     367 SRSGFEIIKI-------RNGW-TQNS-KD----  386 
AAA43449/A     367 SRKGFEMIWD-------PNGW-TDPD-SN----  386 
AAA43575/A     371 SRSGYEMLKV-------PNAL-TDDR-SK----  390 
AAD49005/A     370 SRSGYETFRV-------IGGW-ATANSKL----  390 
AAD49007/A     370 SRSGYETFRV-------VGGW-TTANSKS----  390 
AAF77036/A     367 SRSGFEMIWD-------PNGW-TETD-SS----  386 
AAO46232/A     370 LRSGYETFKV-------IGGW-STPNSKS----  390 
AAO46822/A     367 SRSGFEMIWD-------PNGW-TGTD-SS----  386 
AAO62036/A     369 GRSGFEIIKV-------TEGWINSPS-H-A---  389 
AAO62041/A     367 SRSGFEMIWD-------PNGW-TETD-SS----  386 
AAO62044/A     368 SRSGFEMVWD-------ANGW-VSTD-KD----  387 
AAO62064/A     371 SRSGYEMLKV-------PNAE-TSTQ-SG----  390 
AAT08005/A     367 SRSGFEILKV-------RNGW-IQNS-KE----  386 
AAT37407/A     369 GRSGFEIIKV-------TEGWINSPN-H-A---  389 
AAT37414/A     369 SRSGFEVIKV-------SNGWIDSRN-QEKAAI  393 
AAT65406/A     371 SRSGYEMLKV-------PNAE-TDNQ-SG----  390 
AAX78821/A     370 SRSGYETFKV-------IGGW-STPNSKS----  390 
AAY52584/A     370 SRSGYETFRV-------IGGW-TTANSKS----  390 
AAY87411/A     370 SRSGYETFKV-------IGGW-STANSKS----  390 
ABB17695/A     369 SRSGFEILLI-------EDGW-IKTS-KT----  388 
ABB19439/A     371 SRSGYEMLKV-------PNAE-TDNQ-SG----  390 
ABB19610/A     367 SRSGFEMIWD-------PNGW-TETD-NS----  386 
ABB19747/A     369 SRSGFEILLI-------EDGW-TKTS-KT----  388 
ABB19914/A     368 SRSGFEMVWD-------ANGW-VSTD-KD----  387 
ABB20088/A     368 SRSGFEMVWD-------ANGW-VSTD-KD----  387 
ABB20104/A     369 SRSGFEVLKV-------PNAE-KDPK-SG----  388 
ABB20243/A     371 SRSGYEMLKV-------PNAL-TDDR-SK----  390 
ABB20375/A     371 YRSGYEMLKV-------PDAE-TSTQ-SG----  390 
ABB20475/A     370 SRSGYETFKV-------IGGW-STANSKS----  390 
ABB20501/A     368 LRSGFEMLKI-------PNAG-TDPD-SK----  387 
ABB21754/A     371 SRSGYEMLKV-------PNAL-TDDR-SK----  390 
ABB87198/A     369 SRSGFEILLI-------EDGW-TKTS-KT----  388 
ABB87732/A     368 SRSGFEMVWD-------ANGW-VSTD-KD----  387 
ABB87959/A     369 SRSGFEMLKI-------PNAG-TDPN-SR----  388 
ABB88058/A     369 GRSGFEIIKV-------TEGWINSPNHS-----  389 
ABB88351/A     371 SRSGYEMLKV-------PNAE-TDTQ-SG----  390 
ABC02269/A     370 SRSGYETFKV-------IGGW-STPNSKL----  390 
ABF47958/A     351 SRHGFEMIWD-------PNGW-TETD-SR----  370 
ABG37189/A     370 LRSGYETFKV-------IGGW-SKPNSKL----  390 
ABH04381/A     369 GRSGFEIIKV-------TDGWINSPN-H-A---  389 
ABI30359/A     370 SRSGYETFRV-------IGGW-ATASSKS----  390 
ABI84399/A     369 SRSGFEVIKV-------SNGW-I--N-S-NNQL  389 
ABI84436/A     369 SRSGFEILLI-------EDGW-IKTS-KT----  388 
ABI84475/A     368 SRSGFEMVWD-------ANGW-VSTD-KD----  387 
ABI84973/A     369 GRSGFEIIKV-------TEGWINSPN-H-A---  389 
ABI84982/A     370 SRSGFEMLKI-------PNAE-TDPN-SR----  389 
ABI92217/A     371 SRSGYEMLKV-------PNAE-TDNQ-SG----  390 
ABJ09099/A     367 SRSGFEILRV-------RNGW-VQNS-KE----  386 
ABK32099/A     368 SRSGFEMVWD-------ANGW-VSTD-KD----  387 
ABP49198/A     371 SRSGYEMLKV-------PNAL-TDDR-SK----  390 
ABP49253/A     371 SRSGYEMLKV-------PNAL-TDDR-SK----  390 
ABQ97208/A     370 LRSGYETFKV-------IGGW-STPNSKL----  390 
ABR28683/A     367 SRSGFEMIWD-------PNGW-TETD-SS----  386 
ABR37723/A     371 SRSGYEMLKV-------PNAL-TDDR-SK----  390 
ABS50124/A     367 SRSGFEMIWD-------PNGW-TETD-SS----  386 
ABS89357/A     370 SRSGFEMLKI-------PNAG-TDPN-SK----  389 
ABU95382/A     367 SRSGFEMIWD-------PNGW-TETD-SS----  386 
ABV25648/A     367 SRSGFEMIWD-------PNGW-TETD-SS----  386 
ABV29593/A     367 SRSGFEMIWD-------PNGW-TETD-SS----  386 
ABV82576/A     367 SRSGFEMIWD-------PNGW-TETD-SS----  386 
ABV82587/A     367 SRSGFEMIWD-------PNGW-TETD-SS----  386 
ABW95951/A     347 SRSGFEMIWD-------PNGW-TGTD-SS----  366 
ABX88802/A     370 SRSGYETFRV-------IGGW-TTANSKS----  390 
ABY84687/A     367 SRSGFEMIWD-------PNGW-TGTD-SS----  386 
ACA04655/A     371 SRSGYEMLKV-------PNAE-TDTQ-SG----  390 
ACA04738/A     370 SRSGFEMLKI-------PNAG-TDPN-SR----  389 
ACA04739/A     367 SRSGFEILRI-------KNGW-TQTS-KE----  386 
ACA04740/A     371 SRSGYEMLKV-------PNAL-TDDR-SK----  390 
ACA14299/A     370 SRSGYETFRV-------IGGW-TTANSKS----  390 
ACB70583/A     347 SRSGFEMIWD-------PNGW-TETD-SS----  366 
ACD47117/A     370 SRAGYETFRV-------VGGW-TTANSKS----  390 
ACD65204/A     367 SRSGFEMIWD-------PNGW-TETD-SS----  386 
ACD76864/A     370 SRSGYETFRV-------IGGW-ATANSKS----  390 
ACD85421/A     367 SRSGFEIIKI-------RNGW-TQNS-KD----  386 
ACE73320/A     368 SRSGFEMVWD-------ANGW-VSTD-KD----  387 
ACE76594/A     370 SRSGYETFRV-------LGGW-TTANSKS----  390 
ACE78976/A     370 SRSGYEMLKV-------PNAE-TSTQ-SG----  389 
ACE78981/A     370 SRSGYEMLKV-------PNAE-TDTQ-SG----  389 
ACF20219/A     369 SRSGFEMLKI-------PNAG-TDPN-SR----  388 
ACF25054/A     370 SRSGYETFRV-------IGGW-ATANSKS----  390 
ACF25354/A     369 SRSGFEMLKI-------PNAG-TDPN-SR----  388 
ACF33655/A     369 GRSGFEIIKV-------TEGWINSPN-H-A---  389 
ACF36500/A     370 LRSGYETFKV-------IEGW-SNPNSKL----  390 
ACF93490/A     367 SRSGFEIIKI-------RNGW-TQNS-KD----  386 
ACI41114/A     369 GRSGFEIIKV-------TEGWINSPN-H-A---  389 
ACI48786/A     367 SRSGFEIIKI-------RNGW-TQNS-KD----  386 
ACN65661/A     370 SRSGFEMLKI-------PNAG-TDPN-SR----  389 
ACO24985/A     366 SRSGFEMIWD-------PNGW-TETD-SS----  385 
ACP50710/A     370 SRAGYETFRV-------IGGW-TMANSKS----  390 
ACQ73415/A     368 LRSGFEMLKI-------PNAG-TDPE-SK----  387 
ACR66907/A     369 GRSGFEIIKV-------TEGWINSPN-H-A---  389 
ACR83968/A     367 SRRGFEMIWD-------PDGW-TRTD-DK----  386 
ACS68311/A     371 SRSGYEMLKV-------PNAL-TDDR-SK----  390 
ACS92897/A     370 SRSGYETFKV-------IEGW-SKPNSKL----  390 
ACT67761/A     370 SRLGYETFKV-------IEGW-SNPKSKL----  390 
ACT97062/A     367 SRSGFEILRI-------KNGW-TQTS-KG----  386 
ACV41710/A     367 SRSGFEILRV-------RNGW-VQNS-KE----  386 
ADA71169/A     367 IRKGFEMIWD-------PNGW-TNTD-SD----  386 
ADK70163/A     368 SRSGFEMVWD-------ANGW-VSTD-KD----  387 
ADU17627/A     371 SRSGYEVLRV-------PNAL-TDDR-SK----  390 
ADU20276/A     369 SRSGFEILLI-------EDGW-TKTS-KT----  388 
ADU20397/A     369 GRSGFEIIKV-------TEGWINSPN-H-V---  389 
ADU53220/A     368 SRSGFEMVWD-------ANGW-VSTD-KD----  387 
AEB89856/A     371 SRSGYEMLKV-------PNAL-TDDR-SK----  390 
AEB89867/A     367 SRSGFEILRI-------KNGW-TQTS-KE----  386 
AEK50939/A     371 SRSGYEMLKV-------PNAE-TDNQ-SG----  390 
AEK50961/A     369 SRSGFEMLKI-------PNAG-TDPN-SK----  388 
BAA06132/A     370 LRSGYETFKV-------IGGW-STPNSKT----  390 
BAA06718/A     367 SRRGFEMIWD-------PNGW-TDTD-SK----  386 
BAF03532/A     368 SRSGFEVLLI-------EDGW-IKPS-KT----  387 
BAF03569/A     368 SRSGFEVLLI-------EDGW-IRPS-KT----  387 
BAF34378/A     370 SRSGYETFRV-------IGGW-ATANSKS----  390 
BAF34925/A     370 SRSGYETFRV-------IGGW-ATANSKS----  390 
BAF43417/A     369 SRSGFEILLI-------EDGW-IRTS-KT----  388 
BAF43457/A     367 SRSGFEILRV-------KNGW-TQTS-KE----  386 
BAF43461/A     368 SRSGFEVLLI-------EDGW-IRPS-KT----  387 
BAF46763/A     369 GRSGFEIIKV-------TEGWINSPN-H-A---  389 
BAF46905/A     371 SRSGYEMLKV-------PNAE-TDTQ-SG----  390 
BAF48640/A     370 SRSGYETFRV-------IGGW-TTANSKS----  390 
BAF48644/A     369 GRSGFEIIKV-------TEGWINSPN-H-A---  389 
BAF48646/A     370 SRSGFEMLKI-------PNAG-TDPN-SK----  389 
BAF63047/A     367 LRKGFEMIWD-------PNGW-TDTD-SD----  386 
BAG66251/A     367 SRSGFEILKV-------RDGW-IQNS-KE----  386 
BAG66259/A     369 GRSGFEIIKVTEGWINSPN------H-A-----  389 
BAG66279/A     367 SRSGFEILRV-------KNGW-TQTS-KE----  386 
BAH02172/A     370 LRSGYETFKV-------IGGW-STPNSKS----  390 
BAH70320/A     370 SRSGFEMLKI-------PNAG-TDPN-SR----  389 
BAI39637/A     347 SRSGFEMIWD-------PNGW-SETD-SS----  366 
BAI48894/A     367 SRSGFEMIWD-------PNGW-TETD-SS----  386 
BAI50030/A     369 GRSGFEIIKV-------TEGWINSPN-H-A---  389 
BAJ07979/A     368 SRSGFEVLLI-------EDGW-IRPS-KT----  387 
BAJ10562/A     371 SRSGYEMLKV-------PNAE-TDTQ-SG----  390 
CAA36475/A     345 SRSGFEMIWD-------PNGW-TETD-SS----  364 
CAC36997/A     370 LRSGYETFKV-------IGGW-STPNSKS----  390 
CAD57252/A      367 LRKGFEMIWD-------PNGW-TDTD-SD----  386 
CAO82694/A     378 SRSGFEMIWD-------PNGW-TRTD-DN----  397 
CAP69845/A     368 SRSGFEMVWD-------ANGW-VSTD-KD----  387 

cons           925  * *                               957 

AAA43735/B     394 -L-APSGV-MVSIKEPGWYSFGFE-IK-----D  417 
AAA43743/B     394 -L-APSGV-MVSMKEPGWYSFGFE-IK-----D  417 
AAA43749/B     394 -L-TLSGV-MVSIEEPGWYSFGFE-IK-----D  417

AAU94779/B     394 -L-ALSGV-MVSMEEPGWYSFGFE-IK-----D  417

ABL84344/B     394 -L-TLSGV-MVSMEEPGWYSFGFE-IK-----D  417 
ABO72374/B     394 -L-ALSGV-MVSMEEPGWYSFGFE-IK-----D  417 
ABQ81846/B     394 -L-APSGV-MVSMKEPGWYSFGFE-IK-----D  417

ACU12759/B     394 -L-VFSGV-MVSMKEPGWYSFGFE-IK-----D  417 
BAB32609/B     394 -L-APSGV-MVSMKEPGWYSFGFE-IK-----D  417 
AAA43093/A     388 -I-KERQE-IVSNDNWSGYSGSFI-DYWN--DN  414 
AAA43429/A     387 -Q-IRKQV-IVDNLNWSGYSGSFT-LP-VELTK  414 
AAA43449/A     387 -F-LVKQD-IVAMTDWSGYSGRFV-QH-PELTG  414 
AAA43575/A     391 -P-TQGQT-IVLNTDWSGYSGSFM-DY-W--AE  416 
AAD49005/A     391 -Q-VNRQV-IVDNNNWSGYSGIFS-VE-----G  414 
AAD49007/A     391 -Q-INRQV-IVDSDNWSGYSGIFS-VE-----G  414 
AAF77036/A     387 -F-SVRQD-IVAITDWSGYSGSFV-QH-PELTG  414 
AAO46232/A     391 -Q-INRQV-IVDSNNWSGYSGIFS-VE-----G  414 
AAO46822/A     387 -F-SVKQD-IVAITDWSGYSGSFV-QH-PELTG  414 
AAO62036/A     390 KS-VT-QT-LVSNNDWSGYSGSFI-VE-----N  413 
AAO62041/A     387 -F-SVKQD-IVAITDWSGYSGSFV-QH-PELTG  414 
AAO62044/A     388 -S-NGVQD-IIDNDNWSGYSGSFS-IR-GETTG  415 
AAO62064/A     391 -P-VSHQV-IVNNQNWSGYSGAFI-DY-W--SN  416 
AAT08005/A     387 -Q-IKRQV-VVDNLNWSGYSGSFT-LP-VELTR  414 
AAT37407/A     390 KS-VT-QT-LVSNNDWSGYSGSFI-VE-----N  413 
AAT37414/A     394 ------QT-LVSNNDWSGYSGSFV-IE-----N  413 
AAT65406/A     391 -P-VAHQV-IVNNQNWSGYSGAFI-DY-W--AD  416 
AAX78821/A     391 -Q-VNRQV-IVDNNNRSGYSGIFS-VE-----G  414 
AAY52584/A     391 -Q-INRQV-IVDSDNSSGYSGIFS-VE-----G  414 
AAY87411/A     391 -Q-VNRQV-IVDNNNWSGYSGIFS-VE-----G  414 
ABB17695/A     389 -I-VKKVE-VLNNKNWSGYSGAFT-IP-ITMTG  416 
ABB19439/A     391 -P-VAHQV-IVNNQNWSGYSGAFI-DY-W--AD  416 
ABB19610/A     387 -F-SMKQD-IVAITDWSGYSGSFV-QH-PELTG  414 
ABB19747/A     389 -V-VKKVE-VLNNKNWSGYSGAFT-IP-VTMTG  416 
ABB19914/A     388 -S-NGVQD-IIDNDNWSGYSGSFS-IR-GETTG  415 
ABB20088/A     388 -S-NGVQD-IIDNDNWSGYSGSFS-IR-GETTG  415 
ABB20104/A     389 -I-IERQE-IVDNNNWSGYSGSFI-DY-W--DE  414 
ABB20243/A     391 -P-TQGQT-IVLNTDWSGYSGSFI-DY-W--AK  416 
ABB20375/A     391 -P-VSHQV-IVNNQNWSGYSGSFI-DY-W--SN  416 
ABB20475/A     391 -Q-INRQV-IVDNNNWSGYSGIFS-VE-----G  414 
ABB20501/A     388 -I-KERQE-IVGNDNWSGYSGSFI-DYWN--DN  414 
ABB21754/A     391 -P-TQGQT-IVLNTDWSGYSGSFI-DY-W--AK  416 
ABB87198/A     389 -V-VKKVE-VLNNKNWSGYSGAFT-IP-ITMTS  416 
ABB87732/A     388 -S-NGVQD-IIDNDNWSGYSGSFS-IR-GETTG  415 
ABB87959/A     389 -I-TERQE-IVDNNNWSGYSGSFI-DY-W--DE  414 
ABB88058/A     390 KS-IT-QT-LVSNNDWSGYSGSFI-VK-----T  413 
ABB88351/A     391 -P-ISHQV-IVNNQNWSGYSGAFI-DY-W--AN  416 
ABC02269/A     391 -Q-INRQV-IVDSDNRSGYSGIFS-VE-----G  414 
ABF47958/A     371 -F-SMRQD-VVAMTDRSGYSGSFV-QH-PELTG  398 
ABG37189/A     391 -Q-INRQV-IVDRGNRSGYSGIFS-VE-----G  414 
ABH04381/A     390 KS-VT-QT-LVSNNDWSGYSGSFI-VK-----T  413 
ABI30359/A     391 -Q-TNRQV-IVDNNNWSGYSGIFS-VE-----N  414 
ABI84399/A     390 KV-FN-QT-LVSNNDWSGYSGSFV-IE-----N  413 
ABI84436/A     389 ---IAKKVEVLNNKNWSGYSGAFT-IP-TTMTG  416 
ABI84475/A     388 -S-NGVQD-IIDNDNWSGYSGSFS-IR-GETTG  415 
ABI84973/A     390 KS-VT-QT-LVSNNDWSGYSGSFI-VE-----N  413 
ABI84982/A     390 -I-IERQE-IVDXSNRSGYSGSFI-DY-W--DE  415 
ABI92217/A     391 -P-VAHQV-IVNNQNWSGYSGAFI-DY-W--AD  416 
ABJ09099/A     387 -Q-IKRQV-VVDNLNWSGYSGSFT-LP-VKLTK  414 
ABK32099/A     388 -S-NGVQD-IIDNDNWSGYSGSFS-IR-GETTG  415 
ABP49198/A     391 -P-TQGQT-IVLNTDWSGYSGSFM-DY-W--AE  416 
ABP49253/A     391 -P-TQGQT-IVLNTDWSGYSGSFM-DY-W--AE  416 
ABQ97208/A     391 -Q-INRQV-IVDSGDRSGYSGIFS-VE-----G  414 
ABR28683/A     387 -F-SIKQD-IIALTDWSGYSGSFV-QH-PELTG  414 
ABR37723/A     391 -P-TQGQT-IVLTTDWSGYSGSFI-DY-W--AK  416 
ABS50124/A     387 -F-SIKQD-IIALTDWSGYSGSFV-QH-PELTG  414 
ABS89357/A     390 -I-VERQE-IVDNNNWSGYSGSFI-DY-W--DD  415 
ABU95382/A     387 -F-SVKQD-IVAITDWSGYSGSFV-QH-PELTG  414 
ABV25648/A     387 -F-SIKQD-IIALTDWSGYSGSFV-QH-PELTG  414 
ABV29593/A     387 -F-SVKQD-IVGITDWSGYSGSFV-QH-PELTG  414 
ABV82576/A     387 -F-SVKQD-IIALTDWSGYSGSFV-QH-PELTG  414 
ABV82587/A     387 -F-SMKQD-IIASTDWSGYSGSFV-QH-PELTG  414 
ABW95951/A     367 -F-SVKQD-IVAITDWSGYSGSFV-QH-PELTG  394 
ABX88802/A     391 -Q-VNRQV-IVDNNNWSGYSGIFS-VE-----G  414 
ABY84687/A     387 -F-SIKQD-IIALTDWSGYSGSFV-QH-PELTG  414 
ACA04655/A     391 -P-ISHQV-IVNNQNWSGYSGAFI-DY-W--AN  416 
ACA04738/A     390 -I-AERQE-IVDNNNWSGYSGSFI-DYWD--DD  416 
ACA04739/A     387 -Q-VRKQV-VVDNLNWSGYSGSFT-LP-VELSG  414 
ACA04740/A     391 -P-TQGQT-IVLNTDWSGYSGSFM-DY-W--AE  416 
ACA14299/A     391 -Q-VNRQV-IVDNNNWSGYSGIFS-VE-----S  414 
ACB70583/A     367 -F-SVKQD-IVAITDWSGYSGSFV-QH-PELTG  394 
ACD47117/A     391 -Q-INRQV-IVDSDNLSGYSGIFS-VE-----G  414 
ACD65204/A     387 -F-SIKQD-IIALTDWSGYSGSFV-QH-PELTG  414 
ACD76864/A     391 -Q-TNRQV-IVDNNNWSGYSGIFS-VE-----N  414 
ACD85421/A     387 -Q-IRKQV-IVDNLNWSGYSGSFT-LP-VELTK  414 
ACE73320/A     388 -S-NGVQD-IIDNDNWSGYSGSFS-IR-GETTG  415 
ACE76594/A     391 -Q-INRQV-IVDNNNWSGYSGIFS-VE-----S  414 
ACE78976/A     390 -P-IAHQV-IVNNQNWSGYSGAFI-DY-W--AS  415 
ACE78981/A     390 -P-ISHQV-IVNNQNWSGYSGAFI-DY-W--AN  415 
ACF20219/A     389 -I-TERQE-IVDNNNWSGYSGSFI-DY-W--DE  414 
ACF25054/A     391 -Q-TNRQV-IVGNNNWSGYSGIFS-VE-----G  414 
ACF25354/A     389 -I-TERQE-IVDNNNWSGYSGSFI-DY-W--DE  414 
ACF33655/A     390 KS-IT-QT-LVSNNDWSGYSGSFI-VK-----T  413 
ACF36500/A     391 -Q-INRQV-IVDRGNRSGYSGIFS-VE-----G  414 
ACF93490/A     387 -Q-IIRQV-IINNPNWSGYSGSFT-LP-VELTK  414 
ACI41114/A     390 KS-IT-QT-LVSNNDWSGYSGSFI-VK-----T  413 
ACI48786/A     387 -Q-IRRQV-IIDNPNWSGYSGSFT-LP-VELTK  414 
ACN65661/A     390 -I-AERQE-IVDNNNWSGYSGSFI-DYWN--DN  416 
ACO24985/A     386 -F-SVKQD-IVAITDWSGYSGSFV-QH-PELTG  413 
ACP50710/A     391 -Q-INRQV-IVDSDNRSGYSGIFS-VE-----G  414 
ACQ73415/A     388 -I-KERQE-IVSNDNWSGYSGSFI-DYWN--DN  414 
ACR66907/A     390 KS-VT-QT-LVSNNDWSGYSGSFI-VE-----N  413 
ACR83968/A     387 -F-SVKQD-IIGITDWSGYSGSFV-QH-PELTG  414 
ACS68311/A     391 -P-IQGQT-IVLNTDWSGYSGSFM-DY-W--AE  416 
ACS92897/A     391 -Q-TNRQV-IVERGKRSGYSGIFS-VE-----G  414 
ACT67761/A     391 -Q-INRQV-IVDRGNRSGYSGIFS-VE-----G  414 
ACT97062/A     387 -Q-VRKQI-VVDNLNWSGYSGSFT-LP-VELTG  414 
ACV41710/A     387 -Q-IKRQV-VVDNLNWSGYSGSFT-LP-VELTK  414 
ADA71169/A     387 -F-SVKQD-IVAITDWSGYSGSFV-QH-PELTG  414 
ADK70163/A     388 -S-NGVQD-IIDNDNWSGYSGSFS-IR-GETTG  415 
ADU17627/A     391 -P-TQGQT-IVLNTDWSGYSGSFM-DY-W--AE  416 
ADU20276/A     389 -V-VKKVE-VLNNKNWSGYSGAFT-IP-ITMTN  416 
ADU20397/A     390 KS-IT-QT-LVSNNDWSGYSGSFI-VK-----A  413 
ADU53220/A     388 -S-NGVQD-IIDNDNWSGYSGSFS-IR-GETTG  415 
AEB89856/A     391 -P-IQGQT-IVLNTDWSGYSGSFM-DY-W--AE  416 
AEB89867/A     387 -Q-VRKQI-VVDNLNWSGYSGSFT-LP-VELTG  414 
AEK50939/A     391 -P-VAHQV-IVNNQNWSGYSGAFI-DY-W--AD  416 
AEK50961/A     389 -I-TERQE-IVDNNNWSGYSGSFI-DY-W--DE  414 
BAA06132/A     391 -Q-TNRQV-IIDSDNWSGYSGIFS-VE-----G  414 
BAA06718/A     387 -F-LVKQD-VVAMTDWSGYSGSFV-QH-PELTG  414 
BAF03532/A     388 ---ISKKVEVLNNKNWSGYSGSFT-IP-TAMTS  415 
BAF03569/A     388 ---ISKKVEVLNNKNWSGYSGSFT-IP-TAMTS  415 
BAF34378/A     391 -Q-VDRQV-IVDNNNWSGYSGIFS-VE-----G  414 
BAF34925/A     391 -Q-VNRQV-IVDNNNWSGYSGIFS-VE-----G  414 
BAF43417/A     389 -I-VKKVE-VLNNKNWSGYSGAFT-IP-ITMTS  416 
BAF43457/A     387 -Q-IRKQV-VVDNLNWSGYSGSFT-LP-VELSG  414 
BAF43461/A     388 ---ISKKVEVLNNKNWSGYSGSFT-IP-TAMTS  415 
BAF46763/A     390 KS-VT-QT-LVSNNDWSGYSGSFI-IE-----S  413 
BAF46905/A     391 -P-ISHQV-IVNNQNWSGYSGAFI-DY-W--AN  416 
BAF48640/A     391 -Q-VNRQV-IVDNNNWSGYSGIFS-VE-----G  414 
BAF48644/A     390 KS-VT-QT-LVSNNDWSGYSGSFI-IE-----N  413 
BAF48646/A     390 -I-AERQE-IVDNNNWSGYSGSFI-DYWD--DN  416 
BAF63047/A     387 -F-SVKQD-VVAITDWSGYSGSFV-QH-PELTG  414 
BAG66251/A     387 -Q-IKRQV-VVDNLNWSGYSGSFT-LP-VELTR  414 
BAG66259/A     390 KSITQ--T-LVSNNDWSGYSGSFIVKA-----K  414 
BAG66279/A     387 -Q-VRKQV-VVDNLNWSGYSGSFT-LP-VELSG  414 
BAH02172/A     391 -Q-TNRQV-IVDSNNWSGYSGIFS-VE-----G  414 
BAH70320/A     390 -I-AERQE-IVDNNNWSGYSGSFI-DYWN--DN  416 
BAI39637/A     367 -F-SVKQD-IVAITDWSGYSGSFV-QH-PELTG  394 
BAI48894/A     387 -F-SVKQD-IVAITDWSGYSGSFV-QH-PELTG  414 
BAI50030/A     390 KS-VT-QT-LVSNNDWSGYSGSFI-VE-----N  413 
BAJ07979/A     388 ---IRKKVEVLNNKNWSGYSGSFT-IP-TAMTS  415 
BAJ10562/A     391 -P-VSHQV-IVNNQNWSGYSGAFI-DY-W--AN  416 
CAA36475/A     365 -F-SVKQD-IVAITDWSGYSGSFV-QH-PELTG  392 
CAC36997/A     391 -Q-INRQV-IVDSDNRSGYSGIFS-VE-----G  414 
CAD57252/A      387 -F-SVKQD-VVAITDWSGYSGSFV-QH-PELTG  414 
CAO82694/A     398 -F-SVKQD-IIGMTDWSGYSGSFV-QH-PELTG  425 
CAP69845/A     388 -S-NGVQD-IIDNDNWSGYSGSFS-IR-GETTG  415 

cons                958          ::   . . **  *            990 


AAA43735/B     418 K-KCDVPCIGIEMVHDG-----G-K-E--TWHS  440 
AAA43743/B     418 K-KCDVPCIGIEMVHDG-----G-K-E--TWHS  440 
AAA43749/B     418 K-KCDVPCIGIEMVHDG-----G-K-D--TWHS  440

AAU94779/B     418 K-KCDVPCIGIEMVHDG-----G-K-K--TWHS  440

ABL84344/B     418 K-KCDVPCIGIEMVHDG-----G-K-E--TWHS  440 
ABO72374/B     418 K-KCDVPCIGIEMVHDG-----G-K-E--TWHS  440 
ABQ81846/B     418 K-KCDVPCIGIEMVHDG-----G-K-E--TWHS  440 
ACU12759/B     418 K-KCDVPCIGIEMVHDG-----G-K-E--TWHS  440 
BAB32609/B     418 K-KCDVPCIGIEMVHDG-----G-K-K--TWHS  440   
AAA43093/A     415 S-ECYNPCFYVELIRGR--PEEA-KYV--EWTS  441 
AAA43429/A     415 K-GCLVPCFWVEMIRGK--PEEI---T--IWTS  439 
AAA43449/A     415 L-DCMRPCFWVELIRGR--PREK---TT-IWTS  440 
AAA43575/A     417 G-ECYRACFYVELIRGR--PKED-K-V--WWTS  442 
AAD49005/A     415 K-SCVNRCFYVELIRGR--PQET-R-V--WWTS  440 
AAD49007/A     415 K-SCINRCFYVELIRGR--PQET-R-V--WWTS  440 
AAF77036/A     415 L-DCMRPCFWVELIRGQ--PKEN---T--IWTS  439 
AAO46232/A     415 K-SCINRCFYVELIRGR--QQET-R-V--WWTS  440 
AAO46822/A     415 L-DCIRPCFWVELIRGR--PKES---T--IWTS  439 
AAO62036/A     414 N-GCFQPCFYIELIRGRPNKND--D-V--SWTS  440 
AAO62041/A     415 L-DCMRPCFWVELIRGR--PKEN---T--IWTS  439 
AAO62044/A     416 R-NCTVPCFWVEMIRGQ--PKEK---T--IWTS  440 
AAO62064/A     417 K-ECFNPCFYVELIRGR--PKES-S-V--LWTS  442 
AAT08005/A     415 R-NCLVPCFWVEMIRGK--PEEK---T--IWTS  439 
AAT37407/A     414 N-GCFQPCFYIELIRGRPNKND--D-V--SWTS  440 
AAT37414/A     414 N-GCFQPCFYVELTRGVPNKNE--D-V--SWTS  440 
AAT65406/A     417 R-ECFNPCFYVELIRGR--PKES-S-V--LWTS  442 
AAX78821/A     415 K-SCINRCFYVELIRGR--PQET-R-V--WWTS  440 
AAY52584/A     415 K-SCINRCFYVELIRGR--PKET-R-V--WWTS  440 
AAY87411/A     415 K-SCINRCFYVELIRGR--PQET-R-V--WWTS  440 
ABB17695/A     417 K-QCLVPCFWLEMIRGK--PEER---T-SIWTS  442 
ABB19439/A     417 K-ECFNPCFYVELIRGR--PKES-S-V--LWTS  442 
ABB19610/A     415 L-DCMRPCFWVELIRGR--PKEN---T--IWTS  439 
ABB19747/A     417 K-QCLVPCFWLEMIRGK--PEER---T-SIWTS  442 
ABB19914/A     416 R-NCTVPCFWVEMIRGQ--PKEK---T--IWTS  440 
ABB20088/A     416 R-NCTVPCFWVEMIRGQ--PKEK---T--IWTS  440 
ABB20104/A     415 SNECYNPCFYVELIRGR--PEEA-KYV--WWTS  442 
ABB20243/A     417 G-DCYRACFYVELIRGR--PKED-K-V--WWTS  442 
ABB20375/A     417 K-ECFNPCFYVELIRGR--PKES-S-V--LWTS  442 
ABB20475/A     415 K-GCINRCFYVELIRGR--PQET-R-V--WWTS  440 
ABB20501/A     415 S-ECYNPCFYVELIRGR--PEEA-KYV--EWTS  441 
ABB21754/A     417 E-ECYRACFYVELIRGR--PKED-K-V--WWTS  442 
ABB87198/A     417 K-QCLVPCFWLEMIRGR--PEER---T-SIWTS  442 
ABB87732/A     416 R-NCTVPCFWVEMIRGQ--PKEK---T--IWTS  440 
ABB87959/A     415 SSECYNPCFYVELIRGR--PEEA-KYV--WWTS  442 
ABB88058/A     414 K-DCFQPCFYVELIRGRPNKND--D-V--SWTS  440 
ABB88351/A     417 K-ECFNPCFYVELIRGR--PKES-S-V--LWTS  442 
ABC02269/A     415 K-SCINRCFYVELIRGR--EQET-R-V--WWTS  440 
ABF47958/A     399 L-DCMRPCFWVELIRGL--PEED---A--IWTS  423 
ABG37189/A     415 K-SCINRCFYVELIRGR--KQET-E-V--WWTS  440 
ABH04381/A     414 K-GCFQPCFYVELIRGRPNKND--D-V--SWTS  440 
ABI30359/A     415 K-SCINRCFYVELIRGR--PQET-R-V--WWTS  440 
ABI84399/A     414 N-GCFQPCFYVELTRGVPNKNE--D-V--SWTS  440 
ABI84436/A     417 K-QCLVPCFWLEMIRGK--PEER---T-SIWTS  442 
ABI84475/A     416 R-NCTVPCFWVEMIRGQ--PKEK---T--IWTS  440 
ABI84973/A     414 N-GCFQPCFYIELIRGRTNKND--D-V--SWTS  440 
ABI84982/A     416 ANECYNPCFYVELIRGR--PEEA-KYV--WWTS  443 
ABI92217/A     417 R-ECFNPCFYVELIRGR--PKES-S-V--LWTS  442 
ABJ09099/A     415 R-DCLVPCFWVEMIRGK--PEEK---T--IWTS  439 
ABK32099/A     416 R-NCTVPCFWVEMIRGQ--PKEK---T--IWTS  440 
ABP49198/A     417 G-ECYRACFYVELIRGR--PKED-K-V--WWTS  442 
ABP49253/A     417 G-ECYRACFYVELIRGR--PKED-K-V--WWTS  442 
ABQ97208/A     415 K-SCINRCFYVELIRGR--KQET-R-V--WWTS  440 
ABR28683/A     415 M-DCIRPCFWVELIRGQ--PKES---T--IWTS  439 
ABR37723/A     417 E-ECYRACFYVELIRGR--PKED-K-V--WWTS  442 
ABS50124/A     415 M-NCIRPCFWVELIRGQ--PKES---T--IWTS  439 
ABS89357/A     416 GNECYNPCFYVELIRGR--PEEA-KYV--WWTS  443 
ABU95382/A     415 L-DCMRPCFWVELIRGR--PKEN---T--IWTS  439 
ABV25648/A     415 M-NCIKPCFWVELIRGQ--PKES---T--IWTS  439 
ABV29593/A     415 L-DCMRPCFWVELIRGR--PKEN---T--IWTS  439 
ABV82576/A     415 M-DCIRPCFWVELIRGQ--PKEN---T--IWTS  439 
ABV82587/A     415 M-DCIRPCFWVELIRGQ--PKES---T--IWTS  439 
ABW95951/A     395 L-DCIRPCFWVELIRGR--PKES---T--IWTS  419 
ABX88802/A     415 K-SCINRCFYVELIRGR--PQET-R-V--WWTS  440 
ABY84687/A     415 M-NCIKPCFWVELIRGQ--PKES---T--IWTS  439 
ACA04655/A     417 K-ECFNPCFYVELIRGR--PKES-S-V--LWTS  442 
ACA04738/A     417 N-VCYNPCFYVELIRGR--PEEA-KYV--WWTS  443 
ACA04739/A     415 K-DCLVPCFWVEMIRGK--PEEK---T--IWTS  439 
ACA04740/A     417 G-ECYRACFYVELIRGR--PKED-K-V--WWTS  442 
ACA14299/A     415 K-SCVNRCFYVELIRGR--PQET-R-V--WWTS  440 
ACB70583/A     395 L-DCIRPCFWVELIRGR--PKEG---T--IWTS  419 
ACD47117/A     415 K-SCINRCFYVELIRGR--PQET-R-V--WWTS  440 
ACD65204/A     415 M-NCIKPCFWVELIRGQ--PKES---T--IWTS  439 
ACD76864/A     415 K-SCINRCFYVELIRGR--PQEA-R-V--WWTS  440 
ACD85421/A     415 K-GCLVPCFWVEMIRGK--PEEI---T--IWTS  439 
ACE73320/A     416 R-NCTVPCFWVEMIRGQ--PKEK---T--IWTS  440 
ACE76594/A     415 K-SCINRCFYVELIRGR--PQET-R-V--WWTS  440 
ACE78976/A     416 K-ECFNPCFYVELIRGR--PKES-S-V--LWTS  441 
ACE78981/A     416 K-ECFNPCFYVELIRGR--PKES-S-V--LWTS  441 
ACF20219/A     415 SSECYNPCFYVELIRGR--PEEA-KYV--WWTS  442 
ACF25054/A     415 K-SCINRCFYVELIRGR--PQET-R-V--WWTS  440 
ACF25354/A     415 SNECYNPCFYVELIRGR--PEEA-KYV--WWTS  442 
ACF33655/A     414 K-DCFQPCFYVELIRGRPNKND--D-V--SWTS  440 
ACF36500/A     415 K-SCINRCFYVELIRGR--NQET-E-V--LWTS  440 
ACF93490/A     415 K-GCLVPCFWVEMIRGK--PEET---T--IWTS  439 
ACI41114/A     414 K-DCFQPCFYVELIRGRPNKND--D-V--SWTS  440 
ACI48786/A     415 K-GCLVPCFWVEMIRGK--PEET---T--IWTS  439 
ACN65661/A     417 S-ECYNPCFYVELIRGR--PEEA-KYV--WWTS  443 
ACO24985/A     414 L-DCMRPCFWVELIRGR--PKEN---T--IWTS  438 
ACP50710/A     415 K-TCINRCFYVELIRGR--PQET-R-V--WWTS  440 
ACQ73415/A     415 S-ECYNPCFYVELIRGR--PEEA-KYV--EWTS  441 
ACR66907/A     414 N-GCFQPCFYIELIRGRPNKND--D-V--SWTS  440 
ACR83968/A     415 L-DCMRPCFWVELIRGR--PKEN---T--VWTS  439 
ACS68311/A     417 G-ECYRACFYVELIRGR--PKED-K-V--WWTS  442 
ACS92897/A     415 K-SCINRCFYVELIRGR--KEET-K-V--WWTS  440 
ACT67761/A     415 K-SCINRCFYVELIRGR--KEEN-E-V--LWTS  440 
ACT97062/A     415 K-DCLVPCFWVEMIRGK--PEEK---T--IWTS  439 
ACV41710/A     415 R-NCLVPCFWVEMIRGK--PEEK---T--IWTS  439 
ADA71169/A     415 L-DCIRPCFWVELVRGL--PREN----TTIWTS  440 
ADK70163/A     416 R-NCTVPCFWVEMIRGQ--PKEK---T--IWTS  440 
ADU17627/A     417 G-ECYRACFYVELIRGR--PKED-K-V--WWTS  442 
ADU20276/A     417 K-QCLVPCFWLEMIRGK--PEER---T-SIWTS  442 
ADU20397/A     414 K-DCFQPCFYVELIRGRPNKND--D-V--SWTS  440 
ADU53220/A     416 R-NCTVPCFWVEMIRGQ--PKEK---T--IWTS  440 
AEB89856/A     417 G-DCYRACFYVELIRGR--PKED-K-V--WWTS  442 
AEB89867/A     415 K-DCLVPCFWVEMIRGK--PEEK---T--IWTS  439 
AEK50939/A     417 R-ECFNPCFYVELIRGR--PKES-S-V--LWTS  442 
AEK50961/A     415 SSECYNPCFYVELIRGR--PEEA-KYV--WWTS  442 
BAA06132/A     415 K-SCINRCFYVELIRGR--KQET-R-V--WWTS  440 
BAA06718/A     415 L-DCMRPCFWVELVRGR--PREN----TTIWTS  440 
BAF03532/A     416 K-SCLVPCFWLEMIRGK--PEER---T-SIWTS  441 
BAF03569/A     416 K-SCLVPCFWLEMIRGK--PEER---T-SIWTS  441 
BAF34378/A     415 K-SCVNRCFYVELIRGR--PQET-R-V--WWTS  440 
BAF34925/A     415 K-SCINRCFYVELIRGR--PQET-R-V--WWTS  440 
BAF43417/A     417 K-QCLVPCFWLEMIRGK--PEER---T-SIWTS  442 
BAF43457/A     415 K-DCLVPCFWVEMIRGK--PEEK---T--IWTS  439 
BAF43461/A     416 K-SCLVPCFWLEMIRGK--PEER---T-SIWTS  441 
BAF46763/A     414 N-GCFQPCFYIELIRGR--PNENND-V--SWTS  440 
BAF46905/A     417 K-ECFNPCFYVELIRGR--PKES-S-V--LWTS  442 
BAF48640/A     415 K-SCINRCFYVELIRGR--PQET-R-V--WWTS  440 
BAF48644/A     414 N-GCFQPCFYIELIRGRPNKND--D-V--SWTS  440 
BAF48646/A     417 S-ECYNPCFYVELIRGR--PEEA-KYV--WWTS  443 
BAF63047/A     415 L-DCIRPCFWVELVRGL--PREN----TTIWTS  440 
BAG66251/A     415 R-NCLVPCFWVEMIRGK--PEEK---T--MWTS  439 
BAG66259/A     415 --DCFQPCFYVELIRGR-PNKND-D-V--SWTS  440 
BAG66279/A     415 K-DCLVPCFWVEMIRGK--PEEK---T--IWTS  439 
BAH02172/A     415 K-SCINRCFYVELIRGR--KQET-R-V--WWTS  440 
BAH70320/A     417 S-ECYNPCFYVELIRGR--PEEA-KYV--WWTS  443 
BAI39637/A     395 L-DCIRPCFWVELIRGR--PKES---T--IWTS  419 
BAI48894/A     415 L-DCMRPCFWVELIRGR--PKEN---T--IWTS  439 
BAI50030/A     414 N-GCFQPCFYIELIRGRPNKND--D-V--SWTS  440 
BAJ07979/A     416 K-GCLVPCFWLEMIRGK--PEER---T-SIWTS  441 
BAJ10562/A     417 K-ECLNPCFYVELIRGK--PKES-S-V--LWTS  442 
CAA36475/A     393 L-DCMRPCFWVELIRGR--PNHN---T--IWTS  417 
CAC36997/A     415 K-SCINRCFYVELIRGR--EQET-R-V--WWTS  440 
CAD57252/A     415 L-DCIRPCFWVELVRGL--PREN----TTIWTS  440 
CAO82694/A     426 L-DCMRPCFWVELIRGR--PKEN---T--IWTS  450 
CAP69845/A     416 R-NCTVPCFWVEMIRGQ--PKEK---T--IWTS  440 

cons           991    *   *: :*: :.              * * 1023 


AAA43735/B     441 AATAIYCL-MGSGQLLWDTVTGVDMAL-----  466 
AAA43743/B     441 AATAIYCL-MGSGQLLWDTVTGVDMAL-----  466 
AAA43749/B     441 AATAIYCL-MGSGQLLWDTVTGVDMAL-----  466

AAU94779/B     441 AATAIYCL-MGSGQLLWDTATGVDMAL-----  466 
ABL84344/B     441 AATAIYCL-MGSGQLLWDTVTGVDMAL-----  466 
ABO72374/B     441 AATAIYCL-MGSGQLLWDTVTGVDMAL-----  466

ABQ81846/B     441 AATAIYCL-MGSGQLLWDTVTGVDMAL-----  466

ACU12759/B     441 AATAIYCL-MGSGQLLWDTVTGVNMAL-----  466

BAB32609/B     441 AATAIYCL-MGSGQLLWDTVTGVDMAL-----  466

AAA43093/A     442 NSLIALCG-SPISVGSGSFPDGAQIKYFS---  469 
AAA43429/A     440 SSSIVMCG-VDHKVASWSWHDGAILPFDIDKM  470 
AAA43449/A     441 GSSISFCG-VNSDTVNWSWPDDAELPLTIDK-  470 
AAA43575/A     443 NSIVSMCS-STEFLGQWNWPDGAKIEYFL---  470 
AAD49005/A     441 NSIVAFCG-TSGTYGTGSWPDGANINFMAI--  469 
AAD49007/A     441 NSIIVFCG-TSGTYGTGSWPDGANINFMAI--  469 
AAF77036/A     440 GSSISFCG-VNSDTVGWSWPDGAELPFSIDK-  469 
AAO46232/A     441 NSIVVFCG-TSGTYGTGSWPDGANINFMPI--  469 
AAO46822/A     440 GSSISFCG-VNSDTVGWSWPDGAELPFTIDK-  469 
AAO62036/A     441 NSIVTFCG-LDNEPGSGNWPDGSNIGFMPK--  469 
AAO62041/A     440 GSSISFCG-VNSDTVGWSWPDGAELPFTIDK-  469 
AAO62044/A     441 GSSIAFCG-VNSDTTGWSWPDGALLPFDIDK-  470 
AAO62064/A     443 NSIVALCG-SKERLGSWSWHDGAEIIYFK---  470 
AAT08005/A     440 SSSIVMCG-VDHEIADWSWHDGAILPFDIDKM  470 
AAT37407/A     441 NSIVTFCG-LDNEPGSGNWPDGSNIGFMPK--  469 
AAT37414/A     441 NSIVTFCG-LDNEPGSGNWPDGANIGFMPK--  469 
AAT65406/A     443 NSIVALCG-SKERLGSWSWHDGAEIIYFK---  470 
AAX78821/A     441 NSIVVFCG-TSGTYGTGSWPDGANINFMPI--  469 
AAY52584/A     441 NSIIVFCG-TSGTYGTGSWPDGANINFMPI--  469 
AAY87411/A     441 NSIVVFCG-TSGTYGTGSWPDGANINFMPI--  469 
ABB17695/A     443 SSSTVFCG-VSSEVPGWSWDDGAILPFDIDKM  473 
ABB19439/A     443 NSIVALCG-SKERLGSWSWHDGAEIIYFK---  470 
ABB19610/A     440 GSSISFCG-VNSDTVGWSWPDGAELPFTIDK-  469 
ABB19747/A     443 SSSTVFCG-VSSEVPGWSWDDGAILPFDIDKM  473 
ABB19914/A     441 GSSIAFCG-VNSDTTGWSWPDGALLPFDIDK-  470 
ABB20088/A     441 GSSIAFCG-VNSDTTGWSWPDGALLPFDIDK-  470 
ABB20104/A     443 NSLVALCG-SPVPVGSGSFPDGAQIQYFS---  470 
ABB20243/A     443 NSIVSMCS-STEFLGQWNWPDGAKIEYFL---  470 
ABB20375/A     443 NSIVALCG-SKERLGSWSWHDGAEIIYFK---  470 
ABB20475/A     441 NSIVVFCG-TSGTYGTGSWPDGANINFMPT--  469 
ABB20501/A     442 NSLIALCG-SPIPVGSGSFPDGAQIKYFS---  469 
ABB21754/A     443 NSIVSMCS-STEFLGQWNWPDGAKIEYFL---  470 
ABB87198/A     443 SSSTVFCG-VSSEVPGWSWDDGAILPFDIDKM  473 
ABB87732/A     441 GSSIAFCG-VNSDTTGWSWPDGALLPFDIDK-  470 
ABB87959/A     443 NSLVALCG-SPVPVGSGSFPDGAQIQYFS---  470 
ABB88058/A     441 NSVVTFCG-LDNEPGSGNWPDGSNIGFMPK--  469 
ABB88351/A     443 NSIVALCG-SKERLGSWSWHDGAEIIYFK---  470 
ABC02269/A     441 NSIVVFCG-TSGTYGTGSWPDGADINLMPI--  469 
ABF47958/A     424 GSIISFCG-VNSDTVDWSWPDGAELPFTIDK-  453 
ABG37189/A     441 NSIVVFCG-TSGTYGTGSWPDGADINLMPI--  469 
ABH04381/A     441 NSIVTFCG-LDNEPGSGNWPDGSNIGFMPK--  469 
ABI30359/A     441 NSIVVFCG-TSGTYGTGSWPDGANINFMPI--  469 
ABI84399/A     441 NSIVTFCG-LDNEPGSGNWPDGANIGFMPK--  469 
ABI84436/A     443 SSSTVFCG-VSSEVPGWSWDDGAILPFDIDKM  473 
ABI84475/A     441 GSSIAFCG-VNSDTTSWSWPDGALLPFDIDK-  470 
ABI84973/A     441 NSIVTFCG-LDNEPGSGNWPDGSNIGFMPK--  469 
ABI84982/A     444 NSLIALCG-SPVSVGSGSFPDGAQIQYFS---  471 
ABI92217/A     443 NSIVALCG-SKERLGSWSWHDGAEIIYFK---  470 
ABJ09099/A     440 SSSIVMCG-VDHEIADWSWHDGAILPFDIDKM  470 
ABK32099/A     441 GSSIAFCG-VDSDTTGWSWPDGALLPFDIDK-  470 
ABP49198/A     443 NSIVSMCS-STEFLGQWNWPDGAKIEYFL---  470 
ABP49253/A     443 NSIVSMCS-STEFLGQWNWPDGAKIEYFL---  470 
ABQ97208/A     441 NSIVVFCG-TSGTYGTGSWPDGADIDLMPI--  469 
ABR28683/A     440 GSSISFCG-VNSGTASWSWPDGADLPFTIDK-  469 
ABR37723/A     443 NSIVSMCS-STEFLGQWNWPDGAKIEYFL---  470 
ABS50124/A     440 GSSISFCG-VDSETASWSWPDGADLPFTIDK-  469 
ABS89357/A     444 NSLIALCG-SPFPVGSGSFPDGAQIQYFS---  471 
ABU95382/A     440 GSSISFCG-VSSDTVGWSWPDGAELPFTIDK-  469 
ABV25648/A     440 GSSISFCG-VDSETASWSWPDGADLPFTIDK-  469 
ABV29593/A     440 GSSISFCG-VNSETVGWSWPDGAELPFTIDK-  469 
ABV82576/A     440 GSSISFCG-VNSGTVNWSWPDGADLPFTIDK-  469 
ABV82587/A     440 GSSISFCG-VNSGTANWSWPDGADLPFTIDK-  469 
ABW95951/A     420 GSSISFCG-VNSDTVGWSWPDGAELPFTIDK-  449 
ABX88802/A     441 NSIVVFCG-TSGTYGTGSWPDGANINFMPI--  469 
ABY84687/A     440 GSSISFCG-VDSETASWSWPDGADLPFTIDK-  469 
ACA04655/A     443 NSIVALCG-SKERLGSWSWHDGAEIIYFK---  470 
ACA04738/A     444 NSLIALCG-SPFPVGSGSFPDGAQIQYFS---  471 
ACA04739/A     440 SSSIVMCG-VDYEIADWSWHDGAILPFDIDKM  470 
ACA04740/A     443 NSIVSMCS-STEFLGQWNWPDGAKIEYFL---  470 
ACA14299/A     441 NSIVVFCG-TSGTYGTGSWPDGANINFMPI--  469 
ACB70583/A     420 GSSISFCG-VNSDTVGWSWPDGAELPFTIDK-  449 
ACD47117/A     441 NSIIVFCG-TSGTYGTGSWPDGANIDFMSI--  469 
ACD65204/A     440 GSSISFCG-VDSETVSWSWPDGADLPFTIDK-  469 
ACD76864/A     441 NSIVVFCG-TSGTYGTGSWPDGANINFMPI--  469 
ACD85421/A     440 SSSIVMCG-VDHKIASWSWHDGAILPFDIDKM  470 
ACE73320/A     441 GSSIAFCG-VNSDTTGWSWPDGALLPFDIDK-  470 
ACE76594/A     441 NSIVVFCG-TSGTYGTGSWPDGANINFMPI--  469 
ACE78976/A     442 NSIVALCG-SRERLGSWSWHDGAEIIYFK---  469 
ACE78981/A     442 NSIVALCG-SKERLGSWSWHDGAEIIYFK---  469 
ACF20219/A     443 NSLVALCG-SPVPVGSGSFPDGAQIQYFS---  470 
ACF25054/A     441 NSIVVFCG-TSGTYGTGSWPDGANINFMPI--  469 
ACF25354/A     443 NSLVALCG-SPVSVGSGSFPDGAQIQYFS---  470 
ACF33655/A     441 NSIVTFCG-LDNEPGSGNWPDGSNIGFMPK--  469 
ACF36500/A     441 NSIVVFCG-TSGTYGTGSWPDGADINLMPI--  469 
ACF93490/A     440 SSSIVMCG-VDHKIASWSWHDGAILPFDIDKM  470 
ACI41114/A     441 NSIVTFCG-LDNEPGSGNWPDGSNIGFMPK--  469 
ACI48786/A     440 SSSIVMCG-VDHKIASWSWHDGAILPFDIDKM  470 
ACN65661/A     444 NSLIALCG-SPFPVGSGSFPDGAQIQYFS---  471 
ACO24985/A     439 GSSISFCG-VNSDTVGWSWPDGAELPFTIDK-  468 
ACP50710/A     441 NSIIVFCG-TSGTYGTGSWPDGANINFMAI--  469 
ACQ73415/A     442 NSLIALCG-SPISVGSGSFPDGAQIKYFS---  469 
ACR66907/A     441 NSIVTFCG-LDNEPGSGNWPDGSNIGFMPK--  469 
ACR83968/A     440 GSSISFCG-VNSDTVGWSWPDGAELPFTIDK-  469 
ACS68311/A     443 NSIVSMCS-STEFLGQWNWPDGAKIEYFL---  470 
ACS92897/A     441 NSIVVFCG-TSGTYGTGSWPDGADINLMPI--  469 
ACT67761/A     441 NSIVVFCG-TSGTYGTGSWPDGADINLMPI--  469 
ACT97062/A     440 SSSIVMCG-VDYEVADWSWHDGAIPPFDIDKM  470 
ACV41710/A     440 SSSIVMCG-VDHEIADWSWHDGAILPFDIDKM  470 
ADA71169/A     441 GSSISFCG-VNGDIANWSWPDGAELPFTIDK-  470 
ADK70163/A     441 GSSIAFCG-VDSDTTGWSWPDGALLPFDIDK-  470 
ADU17627/A     443 NSIVSMCS-STEFLGQWNWPDGAKIEYFL---  470 
ADU20276/A     443 SSSTVFCG-VSSEVPGWSWDDGAILPFDIDKM  473 
ADU20397/A     441 NSIVTFCG-LDNEPGSGNWPDGSNIGFMPK--  469 
ADU53220/A     441 GSSIAFCG-VNSDTTGWSWPDGALLPFDIDK-  470 
AEB89856/A     443 NSIVSMCS-STEFLGQWNWPDGAKIEYFL---  470 
AEB89867/A     440 SSSIVMCG-VDYEVADWSWHDGAILPFDIDKM  470 
AEK50939/A     443 NSIVALCG-SKERLGSWSWHDGAEIIYFK---  470 
AEK50961/A     443 NSLVALCG-SPISVGSGSFPDGAQIQYFS---  470 
BAA06132/A     441 NSIVVFCG-TSGTYGTGSWPDGANISFMPI--  469 
BAA06718/A     441 GSSISFCG-VNGDTANWSWPDGAELPFTIDK-  470 
BAF03532/A     442 SSSTVFCG-VSSEVPGWSWDDGAILPFDIDKM  472 
BAF03569/A     442 SSSTVFCG-VSSEVPGWSWDDGAILPFDIDKM  472 
BAF34378/A     441 NSIVVFCG-TSGTYGTGSWPDGANINFMPI--  469 
BAF34925/A     441 NSIVVFCG-TSGTYGTGSWPDGANINFMPI--  469 
BAF43417/A     443 SSSTVFCG-VSSEVPGWSWDDGAILPFDIDKM  473 
BAF43457/A     440 SSSIVMCG-VDYEVADWSWHDGAILPFDIDKM  470 
BAF43461/A     442 SSSTVFCG-VSSEVPGWSWDDGAILPFDIDKM  472 
BAF46763/A     441 NSIVTFCG-LDNEPGSGNWPDGSNIGFMPK--  469 
BAF46905/A     443 NSIVALCG-SKERLGSWSWHDGAEIIYFK---  470 
BAF48640/A     441 NSIVVFCG-TSGTYGTGSWPDGANINFMPI--  469 
BAF48644/A     441 NSIVTFCG-LDNEPGSGNWPDGSNIGFMPK--  469 
BAF48646/A     444 NSLIALCG-SPFPVGSGSFPDGAQIQYFS---  471 
BAF63047/A     441 GSSISFCG-VNSDTADWSWPDGAELPFTIDK-  470 
BAG66251/A     440 SSSIVMCG-VDHEIADWSWHDGAILPFDIDKM  470 
BAG66259/A     441 NSIVTFCGLDNE-PGSGNWPDGSNIG-FMPK-  469 
BAG66279/A     440 SSSIVMCG-VDYEVADWSWHDGAILPFDIDKM  470 
BAH02172/A     441 NSIVVFCG-TSGTYGTGSWPDGANINFMPI--  469 
BAH70320/A     444 NSLIALCG-SPFSVGSGSFPDGAQIQYFS---  471 
BAI39637/A     420 GSSISFCG-VNSDTVSWSWPDGAELPFTIDK-  449 
BAI48894/A     440 GSSISFCG-VDSDTVGWSWPDGAELPFTIDK-  469 
BAI50030/A     441 NSIVTFCG-LDNEPGSGNWPDGSNIGFMPK--  469 
BAJ07979/A     442 SSSTVFCG-VSSEVPGWSWDDGAILPFDIDKM  472 
BAJ10562/A     443 NSIVALCG-SKERLGSWSWHDGAEIIYFK---  470 
CAA36475/A     418 GSSISFCG-VNSDTVGWSWPDGAELPFTIDK-  447 
CAC36997/A     441 NSIVVFCG-TSGTYGTGSWPDGANINFMPI--  469 
CAD57252/A     441 GSSISFCG-VNSDTANWSWPDGAELPFTIDK-  470 
CAO82694/A     451 GSSISFCG-VNSGTVGWSWPDGAELPFTIDK-  480 
CAP69845/A     441 GSSIAFCG-VDSDTTGWSWPDGALLPFDIDK-  470 

cons          1024  :    *          .   .           1055 
